# Systematic Scoring Differences in Large Language Model–Based Clinical Benchmarking Reveal Evaluator-Dependent Divergence: Controlled Evaluation Study

**DOI:** 10.2196/103962

**Published:** 2026-10-26

**Authors:** Rasit Dinc, Nurittin Ardic

**Affiliations:** 1 INVAMED Institute for Medical Innovation, One World Trade Centre, 85th Floor, 285 Fulton Street, New York, NY, United States, 1 (669) 248-1675; 2 Med-International UK Health Agency Ltd, Warwickshire, Nuneaton, United Kingdom

**Keywords:** large language models, clinical benchmarking, LLM-as-judge, evaluation bias, endovascular surgery, PARM framework, interrater reliability, clinical decision support system, criterion interpretation, AI, planning action reflection memory

## Abstract

**Background:**

Large language model (LLM) benchmarking in clinical medicine is increasingly reliant on LLM-based assessment frameworks; in these systems, one AI scores the outputs of another. This practice relies on the assumptions of evaluator neutrality and rubric consistency. These assumptions have not been empirically tested in clinical fields.

**Objective:**

In this study, we aim to directly examine these assumptions by using the PARM (Planning-Action-Reflection-Memory) framework as a theoretical lens to characterize endovascular device selection and clinical reflective reasoning as a clinical field.

**Methods:**

We developed a scoring rubric (C1-C6) with 40 clinical vignettes and 6 criteria across 5 endovascular fields (peripheral artery disease, abdominal aortic aneurysm, chronic venous insufficiency, coronary in-stent restenosis [ISR], and cerebrovascular/neuroendovascular; 8 vignettes per field). Four LLMs were evaluated as clinical decision support systems: GPT-4o (M1), Claude Sonnet 4.6 (M2), Gemini 1.5 Pro (M3), and Llama 3.3 70B (M4). Two LLMs served as independent raters with the same evaluation criteria: Claude Sonnet 4.6 (rater 1) and GPT-4o (rater 2). The rater identity was intentionally disclosed; the primary research question concerned rater scoring behavior rather than the absolute performance of the model. Human assessment was intentionally excluded because its inclusion would have shifted the study question from rater disagreement to rater accuracy, a distinct and secondary question. Interrater reliability was measured using intraclass correlation coefficient (ICC 2,1) two-way random effects modeling.

**Results:**

The combined ICC(2,1) was 0.153 (95% CI 0.007‐0.295). In this exploratory study, the low ICC was not distributed randomly: the magnitude of score differences varied across models (M2 Δ=+5, M4 Δ=+31, M1 Δ=−46, and M3 Δ=−158 points). Scoring behavior analysis identified 2 intrinsically consistent but mutually inconsistent criterion-interpretation models. Rater 1’s scores were most consistent with the reasoning quality approach (partial credit to clinical competence independent of vignette context). Rater 2’s scores were most consistent with the context fidelity approach (near-zero scores for wrong vignette responses independent of reasoning quality). Contrary to the self-preference hypothesis, both raters placed the other higher-performing model above their own. Under rater 1, C6 (PARM reflection) was the lowest-scoring criterion across all 4 models, whereas rater 2 showed greater model-dependent variation in C6 scoring.

**Conclusions:**

AI raters used in LLM-based clinical benchmarking do not behave as interchangeable scoring tools. Their scoring behavior is most consistent with model-specific criterion interpretation patterns that systematically produce different outcomes. The observed low interrater reliability requires interpretation rather than simply being ignored: it reflects structured, direction-specific disagreement concentrated in models with context-tracking failures, not random inconsistency. PARM reflection showed a notable yet evaluator-dependent scoring pattern that warranted further investigation, rather than being interpreted as an evaluator-independent deficit.

## Introduction

The evaluation of large language models (LLMs) in clinical medicine is a critical and rapidly evolving methodological challenge. As LLMs are proposed for clinical decision support systems, guideline interpretation, and device selection, robust evaluation frameworks are essential to determine which systems are safe and effective [1-4]. However, a fundamental practical problem limits human evaluation on a large scale: the volume of content generated by modern LLMs.

A single model evaluated across 40 clinical vignettes with structured multisegment responses can generate 400‐500 pages of content. Evaluating 4 models, as in the present study, would generate approximately 1600‐2000 pages, requiring concurrent expertise in peripheral vascular surgery, interventional cardiology, and neuroendovascular medicine. This limitation has accelerated the adoption of LLM-as-judge evaluation frameworks. LLMs have demonstrated strong clinical knowledge across standard medical benchmarks [5-8], yet the validity of using one such model to evaluate another has received limited scrutiny, despite the proliferation of AI-graded clinical benchmarks [9].

The LLM-as-judge approach is now widespread in the AI evaluation literature [10,11]. However, its adoption has largely preceded the examination of validity assumptions. Just like a trained human expert, implicit in every LLM-as-judge study is the assumption that the evaluation model behaves like an unbiased and consistent scoring instrument [12]. This assumption has not been systematically tested in clinical settings, and several theoretical concerns suggest it may not hold true [11,13]. First, LLMs may exhibit self-preference patterns by generating higher scores for their own outcomes than their competitors. Second, models at the same ability level may exhibit mutually high-scoring behavior regardless of response quality. Third, and most importantly, different LLMs may systematically interpret the same criteria in different ways, generating a structured disagreement that cannot be attributed solely to criterion ambiguity.

This study was designed to empirically examine these phenomena. We constructed a controlled evaluation experiment in which 4 LLMs were evaluated as clinical decision support systems in endovascular medicine, and 2 LLMs acted as independent raters using a predetermined 6-criterion scale. The identities of the raters were deliberately disclosed: 1 rater (Claude Sonnet 4.6) was also rated as M2, while the other (GPT-4o) was rated as M1. This design allowed for the systematic examination of self-assessment patterns, mutually high-scoring behavior, and criterion interpretation differences.

Our primary research question was not which LLM performed best in endovascular device selection (as was also characterized), but whether LLM-based raters applied systematically divergent scoring approaches to identical clinical responses. We present evidence that they did, that the resulting disagreement is structured and clinically interpretable, and that the low interrater reliability under this design requires contextual interpretation rather than being dismissed as a methodological failure. A secondary and exploratory observation was a model- and evaluator-dependent pattern in PARM Reflection scoring that warranted further investigation. This observation requires independent replication before clinical significance can be attributed. Figure 1 illustrates the central methodological hypothesis of the study: an identical scoring rubric may systematically produce different outcomes when interpreted through different evaluator-dependent scoring frameworks.

The primary objective of this exploratory study was to determine whether 2 LLM-based raters applying the same rating criteria produced systematically different scores when evaluating LLM clinical decision support outputs, and if so, to characterize the structure of this divergence. The secondary objective was to assess whether any scoring patterns emerged consistently across both evaluators, independent of their divergent criterion-application approaches.

**Figure 1.**
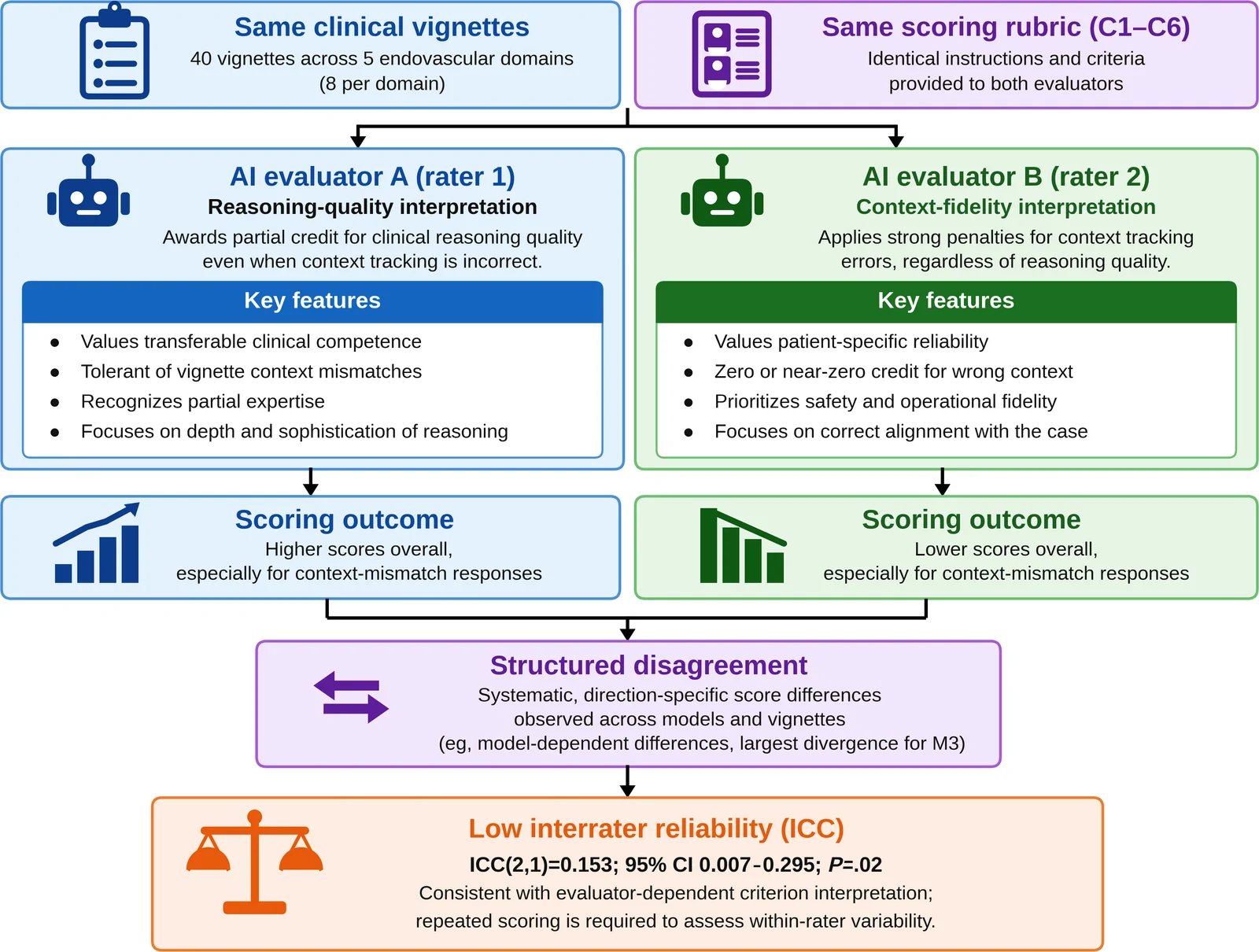
Methodological framework for evaluating interrater agreement and evaluator-dependent scoring divergences. Four large language models (LLMs) generated responses to 40 clinical vignettes and were independently scored by 2 LLM-based raters using the same 6-criterion rubric. Agreement was assessed using intraclass correlation coefficient (ICC 2,1), with criterion-level and model-level analysis used to characterize differences in scoring patterns. The reasoning-quality and context-fidelity interpretations shown are post hoc behavioral characterizations of the observed scoring patterns rather than prespecified evaluator categories. C1–C6: rubric criteria.

## Methods

### Study Design

This was a controlled evaluation study with a 4×2 factorial design: 4 LLMs were evaluated as clinical decision support systems, and 2 LLMs served as independent raters. The primary research question concerned the scoring behavior of the AI rater. Clinical LLM performance was a secondary characterization. All 40 vignettes were prespecified before any model was accessed. The scoring rubric was finalized before any response was scored. The statistical analysis pipeline was documented prior to data extraction.

This is an exploratory proof-of-concept study. All findings should be interpreted as preliminary observations and hypothesis-generating results, rather than confirmatory results. The distinction between the “reasoning-quality” and “context-fidelity” scoring paradigms described in *Structured Scoring Differences: Criterion Interpretation Analysis* section was identified post hoc from the pattern of scoring differences, not preregistered as a hypothesis before scoring began. This taxonomy is proposed here as a framework for future prospective testing.

### Clinical Vignette Development

Forty clinical vignettes were developed across 5 domains: peripheral artery disease (PAD), abdominal aortic aneurysm (AAA), chronic venous insufficiency (CVI), coronary in-stent-restenosis (ISR), and cerebrovascular/neuroendovascular medicine (NEURO; 8 vignettes per domain). The domains were selected to encompass the peripheral vascular, cardiovascular, venous, and neurovascular subspecialties. This allowed for the evaluation of the effects of domain-specific training data on the evaluated model performance and rater scoring behavior.

Within each domain, vignettes were stratified according to clinical demand: vignettes conforming to standard guidelines (n=3 per domain), anatomically or pharmacologically complex cases (n=3), cases requiring explicit contraindication recognition (n=1), and cases requiring conservative or noninvasive management (n=1). Each vignette specified patient demographic information, presenting symptoms, investigational results, and anatomical imaging findings sufficient to guide a specific device selection decision.

A sentinel vignette (V-NEURO-05: fusiform middle cerebral artery aneurysm 3 wk after intracerebral hemorrhage) was selected beforehand as an internal validity anchor. This vignette was chosen because it contained an objectively verifiable contraindication: Dual antiplatelet therapy required by flow-directing devices is contraindicated in a patient at week 3 postintracerebral hemorrhage (ICH), and the correct recognition of this contraindication does not depend on clinical opinion. The correct administration of V-NEURO-05 requires not only the recognition of the contraindication (C3) but also the formal evaluation of an alternative strategy (C6 reflection). This makes it the most demanding vignette in the dataset and a direct stress test for reflective clinical reasoning. Gold-standard consensus expert responses for all 40 vignettes are provided in Multimedia Appendix 1. The complete set of 40 clinical vignettes submitted to the evaluated models is provided in Multimedia Appendix 2.

### Scoring Rubric

A 6-criterion scoring system was developed and finalized before any model was evaluated. Each criterion was scored as 0 (none), 1 (partial), or 2 (complete), resulting in a maximum vignette score of 12 and a maximum model score of 480 (40×12). The 6 criteria were as follows:

1. C1 (Guideline concordance): Concordance of device selection with existing European Society of Cardiology, European Society for Vascular Surgery, (American College of Cardiology/American Heart Association, or domain-specific association guidelines
2. C2 (Anatomical suitability): Specificity and accuracy of device-anatomy matching, including sizing, access route, and biomechanical considerations
3. C3 (Contraindication recognition): Identification of pharmacological, anatomical, or clinical contraindications to the primary device recommendation
4. C4 (Reasoning quality): Coherence, structure, and internal consistency of the clinical decision chain
5. C5 (Hallucination/error rate): Accuracy of factual claims, including device names, clinical trial citations, and anatomical terminology
6. C6 (PARM reflection alignment): Operationalization of the reflection dimension of the PARM framework. C6=2 requires all three of (a) explicit acknowledgement of clinical uncertainty specific to this case, (b) formal articulation of at least one alternative management strategy if the primary strategy is contraindicated, and (c) acknowledgement of evidence limitations stated separately from the recommendation. C6=1 requires at least one element. C6=0 indicates no reflection present. Full scoring anchors are provided in Table S1.1 in Multimedia Appendix 3.

### Evaluated Models (M1-M4)

Four LLMs were evaluated as clinical decision support systems. Each received all 40 vignettes in a single session via the standard chat interface of the respective platform. No system prompting, few-shot examples, or data retrieval augmentation was applied. The models were accessed in standard configuration to reflect real-world clinical use cases.

1. M1: GPT-4o (OpenAI, accessed via chatgpt.com, May 2026) [14].
2. M2: Claude Sonnet 4.6 (Anthropic, accessed via claude.ai, May 2026) [15].
3. M3: Gemini 1.5 Pro (Google DeepMind, accessed via gemini.google.com, May 2026) [16].
4. M4: Llama-3.3-70B-Instruct-Turbo (Meta AI) was assessed via the Together AI Playground in May 2026. Llama-3.1-70B had been prespecified in the study protocol, but Llama 3.3 70B was substituted as the closest available version at the time of evaluation. This constituted a protocol deviation due to model availability [17].

### AI Rater Design and Intentional Disclosure

Two LLMs served as independent raters. Rater 1 was Claude Sonnet 4.6; Rater 2 was GPT-4o. Both raters received the same assessment guidelines, including full criterion definitions, predetermined C6 scoring anchors (Table S1.1 in Multimedia Appendix 3), and the complete vignette text accompanying each model’s response. Raters independently scored each criterion for each vignette-response pair without access to the other rater’s scores. Rater 1 (Claude Sonnet 4.6) was also assessed as M2; rater 2 (GPT-4o) was also assessed as M1. This design feature was intentional and constituted the primary experimental manipulation of the study. It enabled examination of three related questions: (1) whether each rater’s scoring behavior when scoring their own responses differed from their behavior when scoring the responses of other systems (self-scoring patterns); (2) whether high-performing models systematically assigned high scores to one another (mutually high-scoring patterns); and (3) whether the observed scoring behavior reflected criterion-application approaches despite identical assessment guidelines. Complete criterion-level and vignette-level scoring reports for M1, M2, M3, and M4 are provided in Multimedia Appendices 4–7, respectively.

### Intentional Omission of Human Adjudication

This study did not include human expert scoring. This was a conscious design decision: adding human assessment would have shifted the study question from rater disagreement to rater accuracy. In this context, AI rater scores constitute the data, which are the phenomenon under study, rather than measurements that need to be validated against human reality. This design precludes claims of absolute clinical performance, and all comparisons are made against the implemented AI rater framework. Absolute clinical performance validation requires human expert assessment and is a stated priority for future studies.

### Internal Validity Framework

Three internal validity analyses were conducted. First, ordinal rank agreement: whether raters agree on the model rank direction despite cardinal score differences, assessed using Spearman rank correlation on total scores. Second, intercriterion correlation: whether scores within each rater logically vary across criteria (eg, C1 and C2 should show positive correlation; high C5 should be seen concurrently with high C1 in the absence of illusory guidelines). Third, sentinel vignette analysis: interrater agreement on V-NEURO-05 scoring across all four models, where widely recognized high-risk contraindications provide a rubric accuracy test independent of clinical opinion.

### Statistical Analysis

Interrater reliability was quantified using ICC(2,1) (two-way random effects model, absolute agreement, single measures [18,19], with vignette total scores (0‐12) as the unit of assessment). ICC interpretation followed Koo and Li [20]: <0.50=weak, 0.50‐0.75=moderate, 0.75‐0.90=good, and ≥0.90=excellent agreement. Spearman rank correlation was used to assess ordinal agreement in model rankings. All analyses were performed using *SciPy 1.13* and *NumPy 1.26* in Python 3.12. The observed low ICC requires careful contextual interpretation. Low agreement between 2 AI raters applying different criterion-application approaches is not necessarily equivalent to random scoring inconsistency. Instead, the disagreement pattern may reflect systematic differences in how the identical scoring criteria are operationalized among evaluators. The complete statistical analysis pipeline is provided in Multimedia Appendix 8.

### Ethical Considerations

This study used deidentified and anonymized clinical scenarios constructed by the authors based on published case archetypes. No patient data, personal health information, or identifiable clinical records were accessed or used at any stage. All vignettes were fictitious and prepared solely for the purpose of model evaluation. Under these circumstances, a formal ethics committee review was not required in accordance with institutional and JMIR AI editorial policies. This assessment reflects the authors’ own determination based on the nature of the study; no formal Institutional Review Board (IRB) exemption letter was obtained.

## Results

### Model Scores by AI Rater

Table 1 presents the scores assigned by each rater to each evaluated model. Rater 1’s scoring ranked the models as follows: M1 GPT-4o (455/480, 95%)>M2 Claude (441/480, 91%)>M3 Gemini (431/480, 89%)>M4 Llama (270/480, 56%). Rater 2’s scoring ranked the models as follows: M2 Claude (446/480, 93%)>M1 GPT-4o (409/480, 85%)>M4 Llama (301/480, 63%)>M3 Gemini (273/480, 57%). Both raters agreed on a tier separation between M1/M2 (both ≥85%) and M3/M4 (both ≤63%). The full 4-model Spearman rank correlation was ρ=0.60 (*P*=.40), reflecting agreement on tier separation but differing within-tier ordering.

**Table 1.** Scores assigned by each AI rater to each evaluated model (maximum 480).

Models	Rater 1^a^ (Claude), score (%)	Rater 2^b^ (GPT-4o), score (%)	Δ^c^ (R2−R1)
M1 (GPT-4o)	455 (95)	409 (85)	−46^d^
M2 (Claude Sonnet 4.6)	441 (91)	446 (93)	+5
M3 (Gemini 1.5 Pro)	431 (89)	273 (57)	−158^d^
M4 (Llama 3.3 70B)	270 (56)	301 (63)	+31

a Rater 1=Claude Sonnet 4.6 (also evaluated as M2).

b Rater 2=GPT-4o (also evaluated as M1).

c Δ=signed difference (rater 2 score minus rater 1 score).

d Negative values indicate that rater 1 scored higher.

### Interrater Reliability

The combined ICC(2,1) value for all 4 models was 0.153 (95% CI 0.007‐0.295; *F*_159,159_=1.39; *P*=.02; Table 2), which is classified as poor according to Koo and Li [20]. The ICC(2,1) values per model ranged from −0.149 (M4) to +0.048 (M2), all of which are poor. The low reliability between the 2 AI raters whose scoring patterns are most consistent with inconsistent criterion-interpretation approaches is not surprising but an expected result: it shows that the scoring results are sensitive to which rater’s approach is applied. This does not mean that low ICC in general is acceptable; it means that this particular low ICC model should be interpreted in conjunction with the structured disagreement analysis in *Structured Scoring Differences: Criterion Interpretation Analysis* section, not in isolation.

Negative ICC(2,1) point estimates (M1: −0.123 and M4: −0.149) arise when the estimated between-vignette variance component is smaller than expected under the two-way random-effects model, specifically, when within-vignette rater disagreement exceeds any consistent vignette-level signal. This occurs here because both raters placed M1 and M2 at ceiling (≥85%), leaving minimal variance for the ICC to detect. It does not indicate agreement “worse than chance,” but rather that consistent vignette-level discrimination was undetectable at this performance level.

**Table 2.** Interrater reliability of intraclass correlation coefficient (ICC 2,1) by model^a^.

Models	ICC^b^(2,1), (95% CI)	*P* value	Interpretation
M1 (GPT-4o)	−0.123 (−0.331 to 0.132)	.84	Poor
M2 (Claude Sonnet 4.6)	0.048 (−0.267 to 0.352)	.39	Poor
M3 (Gemini 1.5 Pro)	0.029 (−0.139 to 0.240)	.38	Poor
M4 (Llama 3.3 70B)	−0.149 (−0.436 to 0.166)	.83	Poor
Pooled (all models)	0.153 (0.007 to 0.295)	.02	Poor

a ICC(2,1): two-way random effects, absolute agreement, single measures [18]. Poor ICC is classified per Koo and Li [20] and reported without modification; contextual interpretation is provided in the *Structured Scoring Differences: Criterion Interpretation Analysis* and *Main Finding: Criterion-Interpretation Differences Explain the Low ICC* sections.

b ICC: intraclass correlation coefficient.

### Structured Scoring Differences: Criterion Interpretation Analysis

Analysis of the scoring matrices revealed 2 internally consistent, but mutually inconsistent criterion interpretation approaches applied by the raters. These models are defined in terms of the observed scoring outcomes; they are not intended to characterize internal model states or decision processes. Table 3 presents the criterion-level scores for all 4 evaluated models under both raters. This confirms that scoring divergence between the raters was concentrated on criteria C1, C2, and C5 for models with context-tracking errors (M3 and M4 under rater 2), whereas the magnitude and direction of divergence varied across the remaining criteria.

Rater 1’s scoring was most consistent with the reasoning quality approach: responses received higher scores when they contained clinically advanced reasoning, and partial points (C1=1, C2=1, and C4=1) were given for responses demonstrating advanced clinical knowledge, even when applied to the wrong vignette. Under this approach, M3 Gemini scored 431 out of 480 (89%) despite 15 confirmed vignette-context errors across AAA, CVI, ISR, and NEURO domains (Table S1.5 in Multimedia Appendix 3).

Rater 2’s scoring was most consistent with the context fidelity approach: Responses received near-zero scores (C1=0, C2=0, and C5=0) when they addressed the wrong vignette, regardless of the quality of reasoning they contained. Under this approach, M3 Gemini scored 273 out of 480 (57%), a difference of 158 points from rater 1’s scores for the same responses. This difference is the primary reason for the low pooled ICC.

Both criteria application models offer consistent answers to a real ambiguity in clinical AI assessment: what is the appropriate unit of clinical quality? The reasoning-quality approach assesses transferable clinical expertise and acknowledges partial competence. The context-fidelity approach assesses patient-specific reliability and operational safety. Neither is wrong in principle; they answer different questions about the utility of clinical AI, and researchers using LLM-reviewed methods should consider which question their study is designed to answer.

**Table 3.** Criterion-level scores for all 4 evaluated models under both AI raters (maximum 80 per criterion; 40 vignettes × maximum 2 points). Maximum per criterion=80 (40 vignettes × 2).

Criterion	M1 GPT-4o		M2 Claude Sonnet 4.6		M3 Gemini 1.5 Pro		M4 Llama 3.3 70B		Key pattern
	Rater 1 score (%)	Rater 2^a^ score (%)	Rater 1^b^ score (%)	Rater 2 score (%)	Rater 1 score (%)	Rater 2 score (%)	Rater 1 score (%)	Rater 2 score (%)	
C1: Guideline concordance	80 (100)	72 (90)	80 (100)	75 (94)	78 (98)	42 (53)	52 (65)	50 (63)	Both raters agree: M1 consistently guideline-concordant across all domains. M3/M4 diverge substantially under rater 2 (context-fidelity penalization of misassigned responses).
C2: Anatomical appropriateness	79 (99)	71 (89)	80 (100)	77 (96)	79 (99)	41 (51)	42 (53)	48 (60)	M1 and M2 show near-perfect anatomical specificity under rater 1. Rater 2 penalizes M3/M4 misassigned vignettes with C2=0.
C3: Contraindication recognition	77 (96)	66 (83)	80 (100)	77 (96)	78 (98)	45 (56)	46 (58)	53 (66)	M2 achieves maximum C3 under rater 1. M4 Llama: C3=0 on V-NEURO-05 under both raters, the only objectively verifiable zero in the dataset.
C4: Reasoning quality	80 (100)	77 (96)	80 (100)	80 (100)	78 (98)	64 (80)	50 (63)	70 (88)	Both raters agree on reasoning quality for M1 and M2. M4 rater 2 scores higher than rater 1 due to reasoning-quality credit in misassigned PAD^c^ responses.
C5: Hallucination/error rate	79 (99)	63 (79)	80 (100)	62 (78)	79 (99)	44 (55)	80 (100)	43 (54)	C5 near-perfect under Rater 1 for M1, M2, M3, and M4; no hallucinations detected. Rater 2 applies C5=0 to misassigned vignettes regardless of factual content.
C6: PARM^d^ reflection^e^	60 (75)	60 (75)	41 (51)	75 (94)	39 (49)	37 (46)	0 (0)	37 (46)	Evaluator-dependent pattern: 1. For evaluator 1, criterion C6 received the lowest score across all 4 models. For evaluator 2, however, the score for criterion C6 varied by model; the greatest deviation was observed in model M2 (41/80 vs 75/80).
Total (% of maximum 480)	455 (95)	409 (85)	441 (91)	446 (93)	431 (89)	273 (57)	270 (56)	301 (63)	

a Rater 2: GPT-4o (also evaluated as M1).

b Rater 1: Claude Sonnet 4.6 (also evaluated as M2).

c PAD: peripheral artery disease.

d PARM: Planning-Action-Reflection-Memory.

e C6 showed marked evaluator-dependent variation, with divergence observed for M2 (R1=41/80, R2=75/80). Under Rater 1, C6 consistently received the lowest score across M1, M2, M3, and M4. Under Rater 2, C6 showed substantial variation specifically in M2, while remaining notably low for M3 and M4 (37/80 for each).

### Self-Scoring Patterns and High-Tier Concordance

Contrary to the self-preference hypothesis, no rater’s scoring supported their own responses. Rater 1’s scoring placed GPT-4o (M1) in the highest position at 94.8%, which was higher than the score assigned to its own response (M2, 91.9%). Rater 2’s scoring placed Claude (M2) at 92.9%, higher than their own response (M1, 85.2%). This antiself-preference pattern challenges simple self-preference explanations and instead suggests a high-tier concordance pattern: both raters assigned scores ≥85% to the highest-performing models (M1 and M2) and ≤63% to the lower-performing models (M3 and M4; Table 4).

**Table 4.** Scoring matrix: AI rater × evaluated model (% of maximum 480).

AI Rater	M1 (GPT-4o), rater 2’s own, %	M2 (Claude), rater 1’s own, %	M3 (Gemini 1.5 Pro), %	M4 (Llama 3.3 70B), %
Rater 1 (Claude Sonnet 4.6)	94.8^a^	91.9^a^^,b^	89.8	56.3
Rater 2 (GPT-4o)	85.2^a^^,b^	92.9^a^	56.9	62.7

a Cells have 85% (top-tier mutual concordance zone). Both raters’ scoring placed the other top model above their own responses (antiself-preference pattern, n=2 raters; suggestive, requires replication).

b Self-assessment score.

We note that with only 2 raters, the high-tier concordance is suggestive rather than definitive. For this to be considered a robust fact, a consistent finding across a larger group of AI raters from different model families would be needed. Here, we report it as an observation requiring further investigation.

### Llama Scoring Asymmetry

The only scoring model inconsistent with simple high-tier concordance was the M4 Llama asymmetry: rater 2’s scores placed Llama higher than rater 1’s scores (301/480, 63% vs 270/480, 56%). Criterion-level analysis shows that rater 2’s context fidelity approach heavily penalized Gemini’s vignette context errors while being more tolerant of Llama’s responses in the PAD domain (handling incorrect context but being artificially consistent). Rater 1’s reasoning quality approach heavily penalized Llama’s complete lack of reflection (C6=0/80 across all 40 vignettes) more than context errors. This asymmetry is the clearest evidence that the 2 criterion application models penalize different error modes: the context fidelity approach penalizes context loss while tolerating lack of reflection; the reasoning quality approach penalizes lack of reflection but tolerates partial context errors.

### PARM Reflection Scoring Pattern

C6 (PARM reflection) showed a notable but evaluator-dependent scoring pattern (Table 5). Under rater 1, C6 was the criterion that received the lowest score across all 4 models. Under rater 2, however, C6 scores varied substantially across the models, including marked divergence for M2 (C6=75/80 for rater 2, whereas C6=41/80 for rater 1). Several criterion-level observations were nevertheless noteworthy: (1) rater 1 assigned a C6 score of 0 to M4 across all 40 vignettes; (2) V-NEURO-05 was the only vignette to receive a C6=2 from both raters; and (3) C6 scores were similar between raters for M1 and M3.

**Table 5.** C6 planning-action-reflection-memory (PARM) reflection scores by model and rater (out of 80 maximum).

Models	C6 rater 1 (Claude), %	C6 rater 2 (GPT-4o), %	Mean C6 percentage (%)	Notable pattern
M1 (GPT-4o)	60 (75)	60 (75)	75.0	Strongest C6 performance; closest rater C6 agreement. Both raters awarded partial C6 scores (C6=1) on the majority of the vignettes; C6=2 was not achieved on any M1 vignette.
M2 (Claude Sonnet 4.6)^a^	41 (51)	75 (94)	72.5[Table-fn T5_FN1]	Largest C6 rater divergence: Reflect different C6=2 thresholds. Both raters agreed C6=2 exclusively on V-NEURO-05.
M3 (Gemini 1.5 Pro)	39 (49)	37 (46)	47.5	Second closest rater: C6 agreement. Similar low C6 totals across raters.
M4 (Llama 3.3 70B)	0 (0)	37 (46)	23.0	Rater 1 assigned C6=0 across ALL 40 vignettes; rater 2 assigned nonzero C6 scores to a subset of responses.

a For M2, both raters used the same prespecified C6 scoring anchors. The observed difference reflects rater-specific interpretation and application of those anchors when assigned partial or complete credit; no vignette-context misassignment was identified for M2.

These findings do not establish an evaluator-independent reflection deficit. Rather, they show that C6 scoring is particularly influenced by evaluator-specific criterion application, while selected observations, including similar M1 and M3 C6 totals and the V-NEURO-05 sentinel finding, were more consistent across raters. PARM reflection should therefore be considered a criterion requiring further investigation.

### Sentinel Vignette Analysis: V-NEURO-05

V-NEURO-05 (12 mm fusiform middle cerebral artery aneurysm, phases score 8, 3 wk after intracerebral hemorrhage) was the only vignette in which both raters independently assigned C3=0 to the same model, M4 (Llama 3.3 70B), on an objectively verifiable contraindication. This is the only criterion score of 0 on an objectively verifiable item in the entire dataset, as the model’s response failed to identify the need for dual antiplatelet therapy as a contraindication in a patient 3 weeks post-ICH. This scenario represented a high-risk contraindication with relatively limited scoring ambiguity.

M2’s response was the only one in the full dataset that reached C6=2 by both raters: it identified the dual antiplatelet therapy contraindication, formally presented 2 alternative strategies (surgical extracranial-intracranial bypass with aneurysm trapping; delayed flow redirection with phased antiplatelet readministration after 3‐6 mo), specified an antiplatelet scheduling protocol, required multidisciplinary team documentation, and calculated the phases 8 rupture risk as the basis for strategy decision.

The distinctive value of V-NEURO-05 lies in what it requires. The standard information-based response (flow redirection for fusiform morphology) is factually correct, but it becomes clinically inappropriate for this patient. Correct management requires acknowledgment that the standard response is contraindicated, followed by formal evaluation of alternatives—precisely the reflection dimension of the PARM framework. A model that correctly responds to the general situation but fails to recognize the patient-specific contraindication exhibits a clinically concerning capability profile: a high level of knowledge, low contextual safety judgment.

Figure 2 illustrates the model and vignette level distribution of signed interrater score differences, revealing significant heterogeneity in both the magnitude and direction of the evaluator disagreement.

**Figure 2.**
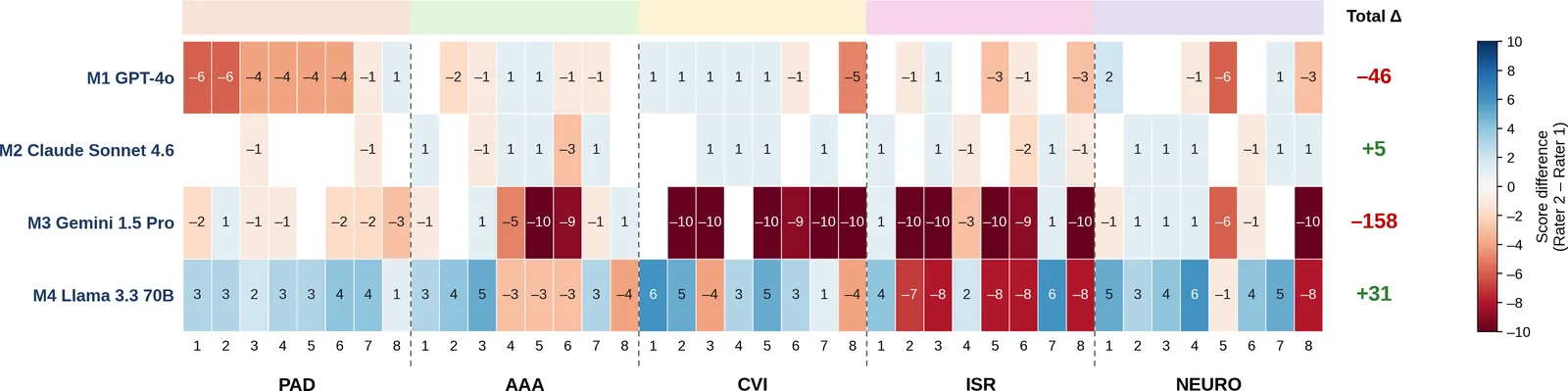
Heatmap of evaluator-dependent scoring differences across models and clinical vignette domains. Values represent the vignette-level signed score differences (rater 2 minus rater 1; maximum score, 12 per vignette). Positive values indicate higher scores assigned by rater 2 (GPT-4o), while negative values indicate higher scores assigned by rater 1 (Claude Sonnet 4.6). Zero differences are shown as empty cells. Total signed differences were −46 for M1 (GPT-4o), +5 for M2 (Claude Sonnet 4.6), –158 for M3 (Gemini 1.5 pro), and +31 for M4 (Llama 3.3 70B). V-NEURO-05 is highlighted as the prespecified sentinel vignette. For M1 and M4, vignette-level values for rater 2 were reconstructed from verified aggregate criterion totals because individual vignette records were not retained. Values for M2 and M3 were derived from retained vignette-level scoring records, as detailed in Multimedia Appendices 5 and 6, respectively. AAA: abdominal aortic aneurysm; CVI: chronic venous insufficiency; ISR: in-stent restenosis; NEURO: cerebrovascular/neuroendovascular domain; PAD: peripheral artery disease.

## Discussion

### Main Finding: Criterion-Interpretation Differences Explain the Low ICC

C6 (PARM reflection) showed a scoring pattern that was dependent on the rater but distinct. Under rater 1, C6 was the lowest-scoring criterion for each of the 4 models evaluated, whereas rater 2 showed greater evaluator-dependent variation, particularly for M2. Nevertheless, cross-rater C6 scores were similar for M1 and M3, while sentinel-vignette analysis identified a specific case where reflective reasoning was clinically consequential. These findings support further investigation into spontaneous reflective output but do not establish an evaluator-independent reflection deficit.

The 0.153 ICC reported in this study should not be read as a simple methodological failure. However, it is indeed low and requires explanation rather than being ignored. The explanation offered here is that 2 AI raters applying inconsistent criterion interpretation approaches to the same evaluation criterion will, by definition, produce a low agreement when scoring the responses that trigger these differences. The more important observation is that the disagreement is not random. It is largest in models with confirmed vignette context errors (M3 Gemini: Δ=−158; M4 Llama: Δ=+31), though M1 also shows a substantial divergence (Δ=−46) attributable primarily to rater-specific scoring thresholds across C1-C5 rather than context-tracking failures, a distinction discussed below.

The M1 divergence (Δ=−46) warrants a separate explanation. Although GPT-4o (M1) had no vignette context errors, rater 1 scored it 46 points higher than rater 2. Criterion-level analysis reveals that this gap is distributed across C1-5. Rater 1 assigned GPT-4o a mean C6 of 75% (60/80), while rater 2 assigned 75% (60/80), near-identical C6 totals. The M1 gap is therefore driven primarily by threshold differences across C1-C5 between the raters on high-quality responses. This reflects the same criterion interpretation divergence documented for other models but without a context-tracking-failure component. This further supports the interpretation that the 2 raters’ scoring approaches represent distinct criterion application philosophies, rather than simply quality detection.

Structured, directional disagreement can be interpreted in a way that random disagreement cannot be interpreted. This situation is consistent with the known instability of LLM under different evaluation framings [21,22]. In these studies, the scoring outcomes systematically vary according to question structure and option order. The important point is that a low ICC value remains evidence of weak absolute agreement among evaluators, even when the disagreement pattern itself is informative. We do not argue that low reliability strengthens evaluator validity. Rather, we argue that the observed disagreement pattern provides insight into the evaluator-dependent criterion application.

Agreement on broad tier separation (both raters’ scores place M1/M2 above M3/M4), together with large numerical differences (158 points on the Gemini scale), reveal that the 2 scoring approaches share a rough model hierarchy but apply different criteria to translate this hierarchy into numbers. The Spearman correlation (ρ=0.60; *P*=.40) is reported here as a descriptive summary of the ordinal agreement across the 4 models. The small sample precludes inferential interpretation at the model ranking level. Therefore, the choice of an AI rater determines not only the magnitude but also the basis of the benchmark results, which is a key finding of the study.

The 2 scoring models identified (reasoning quality and context fidelity) are described as behavioral characterizations of the observed scoring outcomes, not as statements about the intrinsic properties of the model. Throughout this discussion, we deliberately and consistently use phrases such as “most consistent” and “the scoring was most consistent.” Alternative explanations for the observed models cannot be ruled out, and future studies using controlled stimulus designs may reveal them more definitively.

### Self-Scoring Patterns and High-Tier Concordance

An antiself-preference pattern emerged; both raters assigned higher scores to the other high-performing model than to their own responses. First, both AI raters may apply more tolerant scoring to recognized high-quality clinical reasoning, regardless of its source (epistemic generosity). Second, GPT-4o and Claude share large portions of training datasets (medical literature and clinical guidelines), which can be expressed as overlapping training data. This can produce convergent reasoning patterns that each rater’s scoring recognizes and rewards.

The observation of high-tier concordance, whereby both raters consistently assigned scores of 409 out of 480 (85%) or higher to the highest-performing models, may have practical implications for reinforcement learning from AI feedback (RLAIF) pipelines in which the same model family functions as both the evaluator and target system. If high-performing raters systematically favor similarly high-performing models, preference learning processes may generate inflated performance estimates and amplify apparent capability differences that do not fully correspond to meaningful differences in clinical quality. This hypothesis warrants evaluation in future multirater studies.

### PARM Reflection: An Evaluator-Dependent Scoring Pattern

C6 (PARM reflection) demonstrated an evaluator-dependent rather than uniformly convergent scoring pattern. Under rater 1, C6 was the lowest-scoring criterion for each of the 4 models evaluated. Under rater 2, this pattern was not uniformly replicated, with the clearest divergence observed for M2. Nevertheless, cross-rater scores for C6 were similar for M1 and M3, and the sentinel-vignette analysis identified a specific instance where reflective reasoning was clinically consequential.

A clinical decision support system that offers sound advice but fails to address uncertainty and offers no alternatives where the standard approach might be problematic. It ignores limitations of evidence and can be dangerous even when it seems most confident [4]. The PARM Reflection gap is not a minor assessment deficit. It operationalizes the gap between high clinical knowledge and safe clinical reasoning under uncertainty. This finding is consistent with the PARM framework’s emphasis on reflective clinical reasoning as a distinct component of the safe clinical decision-making process [23]: the reflection dimension is what separates clinical intelligence from active clinical reasoning. The clinical relevance of reflection remains plausible. However, the evaluator-dependent C6 scoring observed here precludes interpreting the pattern as a stable characteristic of the evaluated models. Independent replication with additional AI raters and validation by human experts is required.

### Implications of LLM-as-Judge Methodology in Clinical AI

This study contains 4 specific implications for clinical AI researchers using LLM-based evaluation approaches. First, single-evaluator benchmark tests can produce results dependent on criterion interpretation. A study using GPT-4o as a single reviewer will produce the most consistent results with a context fidelity approach; a study using Claude will produce the most consistent results with a reasoning quality approach. Without knowing which approach is valid, benchmark scores cannot be compared between studies using different AI reviewers.

Second, the identity of the AI reviewer should be disclosed, justified, and recorded in advance along with the evaluation criteria. This is similar to the requirement to disclose the identity and training of human reviewers in traditional reliability studies. This is not currently standard practice in clinical AI benchmarking.

Third, the high-tier agreement model, if replicated, suggests that using the same model as both subject and judge, as in RLAIF pipeline [24], can generate systematically enhanced preference predictions for highly skilled models. This is a particular prediction that RLAIF methodology studies should directly examine. More broadly, undetected AI evaluator divergence risks permeating clinical AI training processes [25]. This makes the characterization of systematic evaluator divergence not only a methodological issue but also a concern from a patient safety perspective.

Fourth, the C6 findings illustrate the importance of reporting criterion-level agreement rather than relying solely on aggregate benchmark scores. Criteria showing substantial evaluator-dependent variation should be distinguished from observations that replicate across evaluators, and multievaluator studies should report these patterns separately.

A note on the C6 finding as a rubric-conditional: the models were not explicitly prompted to acknowledge uncertainty, propose alternatives, or state limitations of the evidence. The observed C6 scoring pattern therefore reflects spontaneous reflective output in the absence of an explicit reflection instruction. Whether an explicit system prompt would elicit C6=2-level reflection is a testable question deferred to future work (*Future Directions* section). The C6 finding should be interpreted as a characterization of default model behavior under standard prompting conditions, rather than a statement regarding the maximum achievable reflection performance.

### Limitations

This study has 6 limitations. First, human expert scoring was not performed, which prevents absolute clinical performance claims. All comparisons were made according to the AI rater scoring approach applied. Second, only 2 AI raters were used; the findings regarding self-scoring models and high-tier concordance are suggestive rather than conclusive and need to be replicated with additional raters from different model families. Third, the vignettes were not validated by an independent clinical panel prior to use. Fourth, model access was provided via standard chat interfaces without fixed temperature or seed parameters, leading to potential response variability. Fifth, the extracted criterion-interpretation patterns, including reasoning quality and context fidelity, are not direct measures of model processing but rather behavioral characterizations derived from scoring outcome patterns. Sixth, the scope of this study’s claims is intentionally narrow. With 4 evaluated models and 2 AI raters, no findings can be generalized to LLM evaluator behavior in general. All results are observations from one study configuration and should be interpreted as preliminary evidence warranting prospective investigation. The reasoning-quality and context-fidelity paradigm taxonomy proposed in *Structured Scoring Differences: Criterion Interpretation Analysis* section is a post hoc hypothesis generated from this data, not a preregistered theoretical framework. Future studies should test whether this taxonomy replicates across different model families, clinical domains, and scoring rubrics. In addition, each model response was scored once by each rater; intrarater reliability was not measured. Under stochastic decoding conditions, a single scoring pass cannot fully separate stable criterion interpretation differences from intrarater response variability. Quantifying intrarater reliability through independent rescoring sessions is a priority for future work and would strengthen the claim that the 2 observed scoring patterns are internally consistent. The PARM Reflection findings should be interpreted as preliminary observations requiring independent validation rather than confirmatory evidence for the framework. Additionally, rater 1 (Claude Sonnet 4.6) was also evaluated as M2, and rater 2 (GPT-4o) was also evaluated as M1. This design feature is intentionally disclosed. The structural overlap between rater identity and evaluated model identity is a study limitation that cannot be resolved by disclosure alone and requires replication with independent evaluators.

### Future Directions

Four research directions emerge from this study. First, scoring pattern characterization should be repeated with additional AI raters to determine whether the distinction between reasoning quality and context fidelity is stable across model families or specific to this pair of raters. Second, PARM Reflection performance should be investigated under explicit prompting conditions to determine whether the observed pattern reflects a capability limitation or a default behavior that can be modified through prompt engineering. Third, the high-tier concordance pattern should be investigated in RLAIF pipelines. Fourth, a “sentinel vignette methodology,” consisting of a small set of short histories that are widely recognized, in high demand, and allow for interrater validity testing without the need for full human assessment, should be developed as a standard component of clinical LLM assessment.

### Conclusions

In this exploratory study, 2 AI raters applied to LLM-based clinical benchmarking did not behave as interchangeable scoring instruments. Their scoring is most consistent with model-specific criterion interpretation approaches that systematically produce different results. The low interrater reliability (ICC=0.153) observed in this study reflects a structured, directional disagreement concentrated in models with vignette context errors, not random inconsistency. This requires contextual interpretation. It should not be dismissed as a methodological failure but interpreted as evidence of structured, model-dependent evaluator disagreement.

The antiself-preference pattern and high-tier concordance in the scoring of both raters call into question simplistic accounts of AI rater scoring behavior and suggest that the relationship between the AI rater and the model being evaluated is more complex than characterized by the current LLM-peer-reviewed literature.

Under rater 1, C6 was the lowest-scoring criterion for each of four evaluated models, whereas this pattern was not uniformly replicated under rater 2, where C6 showed substantial model-dependent variation. These findings therefore identify PARM Reflection as a criterion warranting further investigation rather than an evaluator-independent deficit. The V-NEURO-05 case study illustrates the potential clinical relevance of reflective reasoning, with only 1 of the 4 evaluated models receiving a perfect C6 score from both raters. More broadly, the identity of the AI rater and its criterion-application approach are not methodological footnotes when LLMs are used as judges. They can materially influence benchmarking results and should therefore be explicitly reported, prespecified where feasible, and critically examined.

## Supplementary material

10.2196/103962 Multimedia Appendix 1 Gold-standard consensus expert responses for all 40 clinical vignettes.

10.2196/103962 Multimedia Appendix 2 Clinical vignettes submitted to the evaluated models.

10.2196/103962 Multimedia Appendix 3 Prespecified C6 (PARM Reflection) scoring anchors and sentinel vignette documentation.

10.2196/103962 Multimedia Appendix 4 Criterion-level and vignette-level scoring records for M1 (GPT-4o).

10.2196/103962 Multimedia Appendix 5 Criterion-level and vignette-level scoring records for M2 (Claude Sonnet 4.6).

10.2196/103962 Multimedia Appendix 6 Criterion-level and vignette-level scoring records for M3 (Gemini 1.5 Pro).

10.2196/103962 Multimedia Appendix 7 Criterion-level and vignette-level scoring records for M4 (Llama 3.3 70B).

10.2196/103962 Multimedia Appendix 8 Python statistical analysis pipeline.

## Abbreviations

AAA: abdominal aortic aneurysm
C1–C6: scoring criteria 1–6
CVI: chronic venous insufficiency
ICC: intraclass correlation coefficient
ISR: in-stent restenosis
LLM: large language model
NEURO: cerebrovascular/neuroendovascular domain
PAD: peripheral artery disease
PARM: Planning-Action-Reflection-Memory
RLAIF: reinforcement learning from AI feedback
