# Supplementary material for: Systematic Scoring Differences in Large Language Model–Based Clinical Benchmarking Reveal Evaluator-Dependent Divergence: Controlled Evaluation Study
Source: JMIR AI. 2026 Oct 26;5:e103962. doi: 10.2196/103962 (PMC13705216; doi:10.2196/103962)
Supplement: Supplementary file 1 [file ai-v5-e103962-s008.pdf]

Supplementary File S4

Statistical Analysis Pipeline

When AI Grades AI: Systematic Scoring Differences in LLM-Based Clinical Benchmarking Reveal Evaluator-Dependent Divergence

Dinc R, Ardic N | INVAMED / Med-International UK | May 2026

**Authors**	Dinc R, Ardic N
**Affiliation**	INVAMED Institute for Medical Innovation / Med-International UK
**Requirements**	Python 3.12, scipy >= 1.13, numpy >= 1.26
**Install**	pip install scipy numpy
**Purpose**	Reproduces Tables 1-4 and Section 3.1 Spearman correlation from the manuscript

Notes on Score Arrays

**(1)**	All four Rater 1 (Claude Sonnet 4.6) per-vignette criterion-level arrays are original retained records from the Phase 2B scoring session. The full 40-vignette x 6-criterion score matrices with written adjudication rationales are reproduced in Supplementary Files S3a-S3d respectively, which constitute the auditable source for these arrays. Grand totals: M1=455, M2=441, M3=431, M4=270.
**(2)**	RATER2_M2 and RATER2_M3 are ORIGINAL RETAINED RECORDS, documented in: RATER2_M2: For_Rater_RD_Claud_4_6.docx (446/480) RATER2_M3: For_Rater_RD_Gemini.docx (273/480)
**(3)**	RATER2_M1 and RATER2_M4 are RECONSTRUCTED arrays. The per-vignette criterion breakdown for Rater 2 (GPT-4o) scoring M1 and M4 was not retained at individual vignette level in the original scoring session. These arrays were reconstructed to match Rater 2's verified criterion-level aggregate totals from the original scoring records (Supplementary Files S3a, S3d): M1 R2: C1=72 C2=71 C3=66 C4=77 C5=63 C6=60 = 409/480 M4 R2: C1=50 C2=48 C3=53 C4=70 C5=43 C6=37 = 301/480 RATER2_M1 and RATER2_M4 criterion-level vignette arrays were reconstructed to satisfy these verified aggregate criterion totals. All analyses using these arrays should therefore be interpreted as dependent on the stated reconstruction. The ICC computation derives vignette-level totals by summing the reconstructed criterion columns; results for M1 and M4 inter-rater reliability are accordingly reconstruction-dependent.
**(4)**	V-NEURO-05 sentinel vignette (index 36) scores reflect the original adjudicated per-vignette records: M1 R1: [2,2,2,2,2,2] = 12/12 Rationale (from scoring report): "None - full marks. Antiplatelet/post-ICH conflict correctly identified as central issue." M1 R2: [1,1,1,1,1,1] = 6/12 M2 R1: [2,2,2,2,2,2] = 12/12 M2 R2: [2,2,2,2,2,2] = 12/12 (only C6=2 in dataset) M3 R1: [2,2,1,2,2,1] = 10/12 M3 R2: [1,0,0,1,1,1] = 4/12 (per For_Rater_RD_Gemini.docx, original retained record) M4 R1: [1,1,0,1,2,0] = 5/12 M4 R2: [1,1,0,1,1,0] = 4/12

Script Output — All Manuscript Statistics

The tables below reproduce all statistical values reported in the main manuscript.

Table 1: Grand Total Scores

Model	Rater 1 Total	Rater 2 Total	Delta (R2–R1)
M1 GPT-4o†	455/480 (95%)	409/480 (85%)	–46
M2 Claude Sonnet 4.6	441/480 (91%)	446/480 (93%)	+5
M3 Gemini 1.5 Pro	431/480 (89%)	273/480 (57%)	–158

M4 Llama 3.3 70B†	270/480 (56%)	301/480 (63%)	+31

† M1 and M4 Rater 2 vignette-level values are reconstructed from verified aggregate criterion totals. M1 and M4 inter-rater reliability results are accordingly reconstruction-dependent (see Note 3).

Table 2: ICC(2,1) Results

Model	ICC(2,1)	95% CI	F	p-value	Interp.
M1 GPT-4o	–0.123	[–0.331, 0.132]	0.729	0.837	Poor
M2 Claude Sonnet 4.6	0.048	[–0.267, 0.352]	1.099	0.385	Poor
M3 Gemini 1.5 Pro	0.029	[–0.138, 0.241]	1.101	0.379	Poor
M4 Llama 3.3 70B	–0.149	[–0.436, 0.166]	0.740	0.825	Poor
**Pooled (n=160)**	**0.153**	**[0.007, 0.295]**	**1.386**	**0.020**	**Poor**

M1 and M4 ICC values are derived from reconstructed Rater 2 arrays and should be interpreted as reconstruction-dependent (see Note 3).

Table 3: Criterion-Level Totals (out of 80 per criterion)

Criterion	M1 R1	M1 R2	M2 R1	M2 R2	M3 R1	M3 R2	M4 R1	M4 R2
C1 Guideline concordance	80/80 (100%)	72/80 (90%)	80/80 (100%)	75/80 (93%)	78/80 (97%)	42/80 (52%)	52/80 (65%)	50/80 (62%)
C2 Anatomical appropriateness	79/80 (98%)	71/80 (88%)	80/80 (100%)	77/80 (96%)	79/80 (98%)	41/80 (51%)	42/80 (52%)	48/80 (60%)
C3 Contraindication recognition	77/80 (96%)	66/80 (82%)	80/80 (100%)	77/80 (96%)	78/80 (97%)	45/80 (56%)	46/80 (57%)	53/80 (66%)
C4 Reasoning quality	80/80 (100%)	77/80 (96%)	80/80 (100%)	80/80 (100%)	78/80 (97%)	64/80 (80%)	50/80 (62%)	70/80 (87%)
C5 Hallucination/error rate	79/80 (98%)	63/80 (78%)	80/80 (100%)	62/80 (77%)	79/80 (98%)	44/80 (55%)	80/80 (100%)	43/80 (53%)
**C6 PARM Reflection**	60/80 (75%)	60/80 (75%)	41/80 (51%)	75/80 (93%)	39/80 (48%)	37/80 (46%)	0/80 (0%)	37/80 (46%)
**TOTAL**	**455/480**	**409/480**	**441/480**	**446/480**	**431/480**	**273/480**	**270/480**	**301/480**

C6 showed marked evaluator-dependent variation, with the largest divergence observed for M2 (R1=41/80, R2=75/80). Under Rater 1, C6 was consistently the lowest-scoring criterion across M1, M2, M3, and M4. Under Rater 2, C6 diverged substantially for M2 and remained substantially lower for M3 and M4 (37/80 for each). † M1 R1 and M4 R1 criterion totals are from original retained records; M1 R2 and M4 R2 are from reconstructed arrays (see Note 3).

V-NEURO-05 Sentinel Vignette (index 36)

Model	R1 scores (C1–C6)	R1 total	R2 scores (C1–C6)	R2 total	Δ
M1 GPT-4o	[2,2,2,2,2,2]	12/12	[1,1,1,1,1,1]	6/12	–6
M2 Claude Sonnet 4.6	[2,2,2,2,2,2]	12/12	[2,2,2,2,2,2]	12/12	0
M3 Gemini 1.5 Pro	[2,2,1,2,2,1]	10/12	[1,0,0,1,1,1]	4/12	–6
M4 Llama 3.3 70B	[1,1,0,1,2,0]	5/12	[1,1,0,1,1,0]	4/12	–1

M1 R1 = 12/12 per original scoring report: 'None — full marks. Antiplatelet/post-ICH conflict correctly identified as central issue.' M2 (green) is the only model achieving C6=2 under both raters. M3 R2 V-NEURO-05 = [1,0,0,1,1,1]=4/12 per For_Rater_RD_Gemini.docx (original retained

record). Rater 1 matrices reproduced in Supplementary Files S3a–S3d for audit.

Spearman Rank Correlation (Section 3.1)

ρ = 0.6000, p = 0.4000. Descriptive only — n=4 models; not inferential.

Python Source Code

Complete annotated source. Run with Python 3.12+ after installing scipy and numpy.

```
"""
============================================================
Supplementary File S4 — Statistical Analysis Pipeline
============================================================
Study:   When AI Grades AI: Systematic Scoring Differences in
         LLM-Based Clinical Benchmarking Reveal
         Evaluator-Dependent Divergence
Authors: Dinc R, Ardic N
Affil.:  INVAMED Institute for Medical Innovation /
         Med-International UK
Date:    May 2026

Reproduces all statistical results in the main manuscript.
Running this script prints values matching Tables 1-4 and
the Spearman rank correlation in Section 3.1.

Requirements: Python 3.12, scipy >= 1.13, numpy >= 1.26
Install:      pip install scipy numpy

NOTES ON SCORE ARRAYS:
------------------------------------------------------------
(1) All four Rater 1 (Claude Sonnet 4.6) per-vignette
    criterion-level arrays are original retained records from
    the Phase 2B scoring session. The full 40-vignette x
    6-criterion score matrices with written adjudication
    rationales are reproduced in Supplementary Files S3a-S3d
    respectively, which constitute the auditable source for
    these arrays. Grand totals: M1=455, M2=441, M3=431,
    M4=270.

(2) RATER2_M2 and RATER2_M3 are ORIGINAL RETAINED RECORDS,
    documented in:
      RATER2_M2: For_Rater_RD_Claud_4_6.docx (446/480)
      RATER2_M3: For_Rater_RD_Gemini.docx    (273/480)

(3) RATER2_M1 and RATER2_M4 are RECONSTRUCTED arrays.
    The per-vignette criterion breakdown for Rater 2 (GPT-4o)
    scoring M1 and M4 was not retained at individual vignette
    level in the original scoring session. These arrays were
    reconstructed to match Rater 2's verified criterion-level
    aggregate totals from the original scoring records
    (Supplementary Files S3a, S3d):
      M1 R2: C1=72 C2=71 C3=66 C4=77 C5=63 C6=60 = 409/480
      M4 R2: C1=50 C2=48 C3=53 C4=70 C5=43 C6=37 = 301/480
    RATER2_M1 and RATER2_M4 criterion-level vignette arrays
    were reconstructed to satisfy these verified aggregate
    criterion totals. All analyses using these arrays should
    therefore be interpreted as dependent on the stated
    reconstruction. The ICC computation derives vignette-level
    totals by summing the reconstructed criterion columns;
    results for M1 and M4 inter-rater reliability are
    accordingly reconstruction-dependent.

(4) V-NEURO-05 sentinel vignette (index 36) scores reflect
    the original adjudicated per-vignette records:
      M1 R1: [2,2,2,2,2,2] = 12/12
```

```
             Rationale (from scoring report): "None - full
             marks. Antiplatelet/post-ICH conflict correctly
             identified as central issue."
      M1 R2: [1,1,1,1,1,1] = 6/12
      M2 R1: [2,2,2,2,2,2] = 12/12
      M2 R2: [2,2,2,2,2,2] = 12/12 (only C6=2 in dataset)
      M3 R1: [2,2,1,2,2,1] = 10/12
      M3 R2: [1,0,0,1,1,1] = 4/12 (per For_Rater_RD_Gemini.docx, original retained record)
      M4 R1: [1,1,0,1,2,0] = 5/12
      M4 R2: [1,1,0,1,1,0] = 4/12
------------------------------------------------------------
"""

import numpy as np
from scipy import stats

# ============================================================
# SCORE ARRAYS
# 40 rows = V-PAD-01..08, V-AAA-01..08, V-CVI-01..08,
#           V-ISR-01..08, V-NEURO-01..08
# 6 cols  = C1, C2, C3, C4, C5, C6  (scores 0-2)
# Rater 1 = Claude Sonnet 4.6  |  Rater 2 = GPT-4o
# ============================================================

# M1 GPT-4o - Rater 1 (ORIGINAL RETAINED; 455/480)
# Criterion totals: C1=80 C2=79 C3=77 C4=80 C5=79 C6=60
# V-NEURO-05 (row 36): [2,2,2,2,2,2]=12 per original scoring report
RATER1_M1 = np.array([
    [2,2,2,2,2,2],[2,2,2,2,2,2],[2,2,2,2,2,1],[2,2,2,2,2,1],
    [2,2,2,2,2,2],[2,2,2,2,2,2],[2,2,2,2,2,1],[2,2,2,2,1,1],
    [2,2,2,2,2,1],[2,2,2,2,2,2],[2,2,2,2,2,2],[2,2,2,2,2,1],
    [2,2,2,2,2,1],[2,2,2,2,2,2],[2,2,2,2,2,2],[2,2,1,2,2,1],
    [2,2,2,2,2,1],[2,2,2,2,2,1],[2,2,2,2,2,1],[2,2,2,2,2,1],
    [2,2,2,2,2,1],[2,2,2,2,2,1],[2,1,2,2,2,1],[2,2,2,2,2,2],
    [2,2,2,2,2,2],[2,2,2,2,2,1],[2,2,2,2,2,1],[2,2,2,2,2,2],
    [2,2,2,2,2,2],[2,2,2,2,2,2],[2,2,2,2,2,2],[2,2,2,2,2,2],
    [2,2,1,2,2,1],[2,2,2,2,2,2],[2,2,2,2,2,2],[2,2,2,2,2,2],
    [2,2,2,2,2,2],[2,2,1,2,2,1],[2,2,2,2,2,1],[2,2,2,2,2,2],
])

# M1 GPT-4o - Rater 2 (RECONSTRUCTED; 409/480)
# Criterion totals: C1=72 C2=71 C3=66 C4=77 C5=63 C6=60
# V-NEURO-05 (row 36): [1,1,1,1,1,1]=6
RATER2_M1 = np.array([
    [1,1,1,1,1,1],[1,1,1,1,1,1],[1,1,1,2,1,1],[1,1,1,2,1,1],
    [1,1,2,2,1,1],[1,1,2,2,1,1],[2,2,2,2,1,1],[2,2,2,2,1,2],
    [2,2,2,2,1,2],[2,2,2,2,1,1],[2,2,2,2,1,2],[2,2,2,2,2,2],
    [2,2,2,2,2,2],[2,2,2,2,2,1],[2,2,1,2,2,2],[2,2,1,2,2,1],
    [2,2,2,2,2,2],[2,2,2,2,2,2],[2,2,2,2,2,2],[2,2,2,2,2,2],
    [2,2,2,2,2,2],[2,1,2,2,2,1],[2,2,2,2,1,1],[1,1,1,2,1,1],
    [2,2,2,2,2,2],[2,2,1,2,2,1],[2,2,2,2,2,2],[2,2,2,2,2,2],
    [2,2,1,2,1,1],[2,2,2,2,2,1],[2,2,2,2,2,2],[2,2,1,2,1,1],
    [2,2,2,2,2,2],[2,2,2,2,2,2],[2,2,2,2,2,2],[2,2,1,2,2,2],
    [1,1,1,1,1,1],[2,2,1,2,2,1],[2,2,2,2,2,2],[2,2,1,2,1,1],
])

# M2 Claude Sonnet 4.6 - Rater 1 (ORIGINAL RETAINED; 441/480)
# Criterion totals: C1=80 C2=80 C3=80 C4=80 C5=80 C6=41
# V-NEURO-05 (row 36): [2,2,2,2,2,2]=12
RATER1_M2 = np.array([
    [2,2,2,2,2,1],[2,2,2,2,2,1],[2,2,2,2,2,1],[2,2,2,2,2,1],
    [2,2,2,2,2,1],[2,2,2,2,2,1],[2,2,2,2,2,1],[2,2,2,2,2,1],
    [2,2,2,2,2,1],[2,2,2,2,2,1],[2,2,2,2,2,1],[2,2,2,2,2,1],
    [2,2,2,2,2,1],[2,2,2,2,2,1],[2,2,2,2,2,1],[2,2,2,2,2,1],
    [2,2,2,2,2,1],[2,2,2,2,2,1],[2,2,2,2,2,1],[2,2,2,2,2,1],
    [2,2,2,2,2,1],[2,2,2,2,2,1],[2,2,2,2,2,1],[2,2,2,2,2,1],
    [2,2,2,2,2,1],[2,2,2,2,2,1],[2,2,2,2,2,1],[2,2,2,2,2,1],
    [2,2,2,2,2,1],[2,2,2,2,2,1],[2,2,2,2,2,1],[2,2,2,2,2,1],
```

```
    [2,2,2,2,2,1],[2,2,2,2,2,1],[2,2,2,2,2,1],[2,2,2,2,2,1],
    [2,2,2,2,2,2],[2,2,2,2,2,1],[2,2,2,2,2,1],[2,2,2,2,2,1],
])

# M2 Claude Sonnet 4.6 - Rater 2 (ORIGINAL RETAINED; 446/480)
# Criterion totals: C1=75 C2=77 C3=77 C4=80 C5=62 C6=75
# V-NEURO-05 (row 36): [2,2,2,2,2,2]=12
RATER2_M2 = np.array([
    [2,2,2,2,1,2],[2,2,2,2,1,2],[1,2,2,2,1,2],[2,2,2,2,1,2],
    [2,2,2,2,1,2],[2,2,2,2,1,2],[1,2,2,2,2,1],[2,2,2,2,1,2],
    [2,2,2,2,2,2],[2,2,2,2,1,2],[2,1,2,2,1,2],[2,2,2,2,2,2],
    [2,2,2,2,2,2],[1,1,2,2,1,1],[2,2,2,2,2,2],[2,2,2,2,1,2],
    [2,1,2,2,2,2],[2,2,2,2,2,1],[2,2,2,2,2,2],[2,2,2,2,2,2],
    [2,2,2,2,2,2],[2,2,2,2,1,2],[2,2,2,2,2,2],[2,2,2,2,1,2],
    [2,2,2,2,2,2],[2,2,2,2,2,1],[2,2,2,2,2,2],[2,2,1,2,1,2],
    [2,2,2,2,1,2],[1,2,1,2,1,2],[2,2,2,2,2,2],[2,2,1,2,1,2],
    [2,2,2,2,1,2],[2,2,2,2,2,2],[2,2,2,2,2,2],[2,2,2,2,2,2],
    [2,2,2,2,2,2],[1,2,2,2,2,1],[2,2,2,2,2,2],[2,2,2,2,2,2],
])

# M3 Gemini 1.5 Pro - Rater 1 (ORIGINAL RETAINED; 431/480)
# Criterion totals: C1=78 C2=79 C3=78 C4=78 C5=79 C6=39
# V-NEURO-05 (row 36): [2,2,1,2,2,1]=10
RATER1_M3 = np.array([
    [1,2,2,2,2,1],[2,2,2,2,2,1],[2,2,2,2,2,1],[2,2,2,2,2,1],
    [2,2,2,2,2,1],[2,2,2,2,2,1],[2,2,2,2,2,1],[2,2,2,2,2,1],
    [2,2,2,2,2,1],[2,2,2,2,1,1],[2,2,2,2,2,1],[1,1,2,1,2,0],
    [2,2,2,2,2,1],[2,2,2,2,2,1],[2,2,2,2,2,1],[2,2,2,2,2,1],
    [2,2,2,2,2,1],[2,2,2,2,2,1],[2,2,2,2,2,1],[2,2,2,2,2,1],
    [2,2,2,2,2,1],[2,2,2,1,2,1],[2,2,2,2,2,1],[2,2,2,2,2,1],
    [2,2,2,2,2,1],[2,2,2,2,2,1],[2,2,2,2,2,1],[2,2,2,2,2,1],
    [2,2,2,2,2,1],[2,2,2,2,2,1],[2,2,2,2,2,1],[2,2,2,2,2,1],
    [2,2,1,2,2,1],[2,2,2,2,2,1],[2,2,2,2,2,1],[2,2,2,2,2,1],
    [2,2,1,2,2,1],[2,2,2,2,2,1],[2,2,2,2,2,1],[2,2,2,2,2,1],
])

# M3 Gemini 1.5 Pro - Rater 2 (ORIGINAL RETAINED; 273/480)
# Total: 273/480 | C1=42 C2=41 C3=45 C4=64 C5=44 C6=37
# V-NEURO-05 (row 36): [1,0,0,1,1,1]=4 per For_Rater_RD_Gemini.docx (original retained record)
RATER2_M3 = np.array([
    [1,1,1,2,2,1],[2,2,2,2,2,2],[2,2,1,2,2,1],[2,2,2,2,1,1],
    [2,2,2,2,1,2],[1,1,2,2,1,2],[1,1,2,2,2,1],[1,1,1,2,2,1],
    [2,2,2,2,1,1],[1,1,2,2,2,2],[2,2,2,2,2,2],[0,0,1,1,0,0],
    [0,0,0,1,0,0],[0,0,1,1,0,0],[1,1,2,2,2,2],[2,2,2,2,2,2],
    [2,2,2,2,2,1],[0,0,0,1,0,0],[0,0,0,1,0,0],[2,2,2,2,2,1],
    [0,0,0,1,0,0],[0,0,0,1,0,0],[0,0,0,1,0,0],[0,0,0,1,0,0],
    [2,2,2,2,2,2],[0,0,0,1,0,0],[0,0,0,1,0,0],[1,1,1,2,2,1],
    [0,0,0,1,0,0],[0,0,1,1,0,0],[2,2,2,2,2,2],[0,0,0,1,0,0],
    [2,2,1,2,1,1],[2,2,2,2,2,2],[2,2,2,2,2,2],[2,2,2,2,2,2],
    [1,0,0,1,1,1],[2,2,1,2,2,1],[2,2,2,2,2,1],[0,0,0,1,0,0],
])

# M4 Llama 3.3 70B - Rater 1 (ORIGINAL RETAINED; 270/480)
# Criterion totals: C1=52 C2=42 C3=46 C4=50 C5=80 C6=0
# V-NEURO-05 (row 36): [1,1,0,1,2,0]=5
RATER1_M4 = np.array([
    [1,1,1,1,2,0],[1,1,1,1,2,0],[1,1,1,1,2,0],[1,1,2,1,2,0],
    [1,1,1,1,2,0],[1,1,1,1,2,0],[1,1,1,1,2,0],[1,1,1,1,2,0],
    [2,1,1,1,2,0],[1,1,1,1,2,0],[1,1,1,1,2,0],[1,1,1,1,2,0],
    [1,1,1,1,2,0],[1,1,1,1,2,0],[2,2,1,2,2,0],[1,1,1,1,2,0],
    [1,1,1,1,2,0],[1,1,1,1,2,0],[1,1,1,1,2,0],[2,1,2,1,2,0],
    [1,1,1,1,2,0],[1,1,1,1,2,0],[1,1,1,1,2,0],[1,1,1,1,2,0],
    [2,1,1,2,2,0],[2,1,1,2,2,0],[2,1,2,2,2,0],[2,1,1,2,2,0],
    [2,1,2,2,2,0],[2,2,1,2,2,0],[1,1,1,1,2,0],[2,1,2,2,2,0],
    [1,1,1,1,2,0],[2,1,2,2,2,0],[1,1,1,1,2,0],[1,1,1,1,2,0],
    [1,1,0,1,2,0],[1,1,1,1,2,0],[1,1,1,1,2,0],[2,1,2,2,2,0],
])
```

```
# M4 Llama 3.3 70B - Rater 2 (RECONSTRUCTED; 301/480)
# Criterion totals: C1=50 C2=48 C3=53 C4=70 C5=43 C6=37
# V-NEURO-05 (row 36): [1,1,0,1,1,0]=4
RATER2_M4 = np.array([
    [1,1,2,2,1,2],[1,1,2,2,1,2],[1,1,2,2,1,1],[2,1,2,2,1,2],
    [2,2,1,2,1,1],[2,2,2,2,1,1],[2,2,2,2,1,1],[1,1,1,2,1,1],
    [2,2,2,2,1,1],[2,2,2,2,1,1],[2,2,2,2,1,2],[0,0,1,2,0,0],
    [0,0,1,2,0,0],[0,0,1,2,0,0],[2,2,2,2,2,2],[0,0,1,1,0,0],
    [2,2,2,2,2,2],[2,2,2,2,2,1],[0,0,1,1,0,0],[2,2,2,2,2,1],
    [2,2,2,2,2,1],[2,1,1,2,2,1],[1,1,1,2,1,1],[0,0,1,1,0,0],
    [2,2,2,2,2,2],[0,0,0,1,0,0],[0,0,0,1,0,0],[2,2,1,2,2,1],
    [0,0,0,1,0,0],[0,0,0,1,0,0],[2,2,2,2,2,2],[0,0,0,1,0,0],
    [2,2,2,2,2,1],[2,2,2,2,2,2],[2,2,1,2,2,1],[2,2,2,2,2,2],
    [1,1,0,1,1,0],[2,2,1,2,2,1],[2,2,2,2,2,1],[0,0,0,1,0,0],
])


# ============================================================
# ICC(2,1) — Two-way random, absolute agreement, single measures
# Shrout & Fleiss (1979); McGraw & Wong (1996)
# ============================================================

def icc_21(data):
    n, k = data.shape
    gm = np.mean(data)
    SSt = np.sum((data - gm) ** 2)
    SSr = k * np.sum((np.mean(data, axis=1) - gm) ** 2)
    SSc = n * np.sum((np.mean(data, axis=0) - gm) ** 2)
    SSe = SSt - SSr - SSc
    dfr = n - 1;  dfe = (n - 1) * (k - 1)
    MSr = SSr / dfr;  MSc = SSc / (k - 1);  MSe = SSe / dfe
    icc = (MSr - MSe) / (MSr + (k-1)*MSe + k*(MSc-MSe)/n)
    F   = MSr / MSe
    p   = 1 - stats.f.cdf(F, dfr, dfe)
    a   = 0.05
    Flo = F / stats.f.ppf(1-a/2, dfr, dfe)
    Fhi = F * stats.f.ppf(1-a/2, dfe, dfr)
    dlo = Flo + (k-1) + k*(MSc-MSe)/(n*MSe)
    dhi = Fhi + (k-1) + k*(MSc-MSe)/(n*MSe)
    return icc, F, dfr, dfe, p, (Flo-1)/dlo, (Fhi-1)/dhi


def vt(arr): return arr.sum(axis=1)

R1_M1=vt(RATER1_M1); R2_M1=vt(RATER2_M1)
R1_M2=vt(RATER1_M2); R2_M2=vt(RATER2_M2)
R1_M3=vt(RATER1_M3); R2_M3=vt(RATER2_M3)
R1_M4=vt(RATER1_M4); R2_M4=vt(RATER2_M4)

model_data = {
    "M1 GPT-4o":            (R1_M1, R2_M1),
    "M2 Claude Sonnet 4.6": (R1_M2, R2_M2),
    "M3 Gemini 1.5 Pro":    (R1_M3, R2_M3),
    "M4 Llama 3.3 70B":     (R1_M4, R2_M4),
}

def interp(v):
    for t,l in [(0.50,"Poor"),(0.75,"Moderate"),
                (0.90,"Good"),(float('inf'),"Excellent")]:
        if v < t: return l
    return "Excellent"


# ============================================================
# OUTPUT
# ============================================================

print("=" * 65)
```

```
print("Supplementary File S4 - Statistical Analysis Pipeline")
print("When AI Grades AI | Dinc R, Ardic N | May 2026")
print("=" * 65)

locked = {"M1 GPT-4o":(455,409),"M2 Claude Sonnet 4.6":(441,446),
          "M3 Gemini 1.5 Pro":(431,273),"M4 Llama 3.3 70B":(270,301)}

print("\n--- Table 1: Grand Total Scores ---")
print(f"  {'Model':<25} {'R1 Total':>10} {'R2 Total':>10} {'Delta':>7}")
print("  " + "-"*55)
r1_tots=[]; r2_tots=[]
for name,(r1,r2) in model_data.items():
    t1,t2=int(r1.sum()),int(r2.sum())
    r1_tots.append(t1); r2_tots.append(t2)
    e1,e2=locked[name]
    m1="OK" if t1==e1 else f"ERR(exp {e1})"
    m2="OK" if t2==e2 else f"ERR(exp {e2})"
    print(f"  {name:<25} {t1:>6}/480 {m1}  {t2:>6}/480 {m2}  {t2-t1:>+7}")

print("\n--- Table 3: Criterion-Level Totals ---")
all_arrs=[RATER1_M1,RATER2_M1,RATER1_M2,RATER2_M2,
          RATER1_M3,RATER2_M3,RATER1_M4,RATER2_M4]
col_lbl=["M1R1","M1R2","M2R1","M2R2","M3R1","M3R2","M4R1","M4R2"]
print(f"  {'Crit':<5} " + " ".join(f"{l:>6}" for l in col_lbl))
print("  " + "-"*56)
for ci,c in enumerate(["C1","C2","C3","C4","C5","C6"]):
    vals=[int(a[:,ci].sum()) for a in all_arrs]
    print(f"  {c:<5} " + " ".join(f"{v:>6}" for v in vals))

print("\n--- Table 2: ICC(2,1) Results ---")
print(f"  {'Model':<25} {'ICC':>8} {'95% CI':>22} {'F':>8} {'p':>8}  Interp")
print("  " + "-"*78)
for name,(r1,r2) in model_data.items():
    icc,F,_,_,p,lo,hi=icc_21(np.column_stack([r1,r2]))
    print(f"  {name:<25} {icc:>8.4f}  [{lo:.3f}, {hi:.3f}]  {F:>8.3f} {p:>8.4f}  {interp(icc)}")
all_r1=np.concatenate([r for r,_ in model_data.values()])
all_r2=np.concatenate([r for _,r in model_data.values()])
icc_p,F_p,_,_,p_p,lo_p,hi_p=icc_21(np.column_stack([all_r1,all_r2]))
print("  " + "-"*78)
print(f"  {'POOLED (n=160)':<25} {icc_p:>8.4f}  [{lo_p:.3f}, {hi_p:.3f}]  {F_p:>8.3f} {p_p:>8.4f}  {
interp(icc_p)}")

print("\n--- Spearman Rank Correlation (Section 3.1) ---")
rho,rho_p=stats.spearmanr(np.array(r1_tots),np.array(r2_tots))
print(f"  rho = {rho:.4f}, p = {rho_p:.4f}")
print(f"  Descriptive only - n=4 models; not inferential")

print("\n--- Table 4: C6 PARM Reflection ---")
print(f"  {'Model':<25} {'R1 C6/80':>10} {'R2 C6/80':>10}  {'R1%':>5} {'R2%':>5}")
print("  " + "-"*58)
for name,(r1a,r2a) in [
    ("M1 GPT-4o",(RATER1_M1,RATER2_M1)),
    ("M2 Claude Sonnet 4.6",(RATER1_M2,RATER2_M2)),
    ("M3 Gemini 1.5 Pro",(RATER1_M3,RATER2_M3)),
    ("M4 Llama 3.3 70B",(RATER1_M4,RATER2_M4)),
]:
    c6r1=int(r1a[:,5].sum()); c6r2=int(r2a[:,5].sum())
    print(f"  {name:<25} {c6r1:>8}/80 {c6r2:>8}/80  {100*c6r1//80:>4}% {100*c6r2//80:>4}%")

print("\n--- V-NEURO-05 Sentinel Vignette (index 36) ---")
for name,(r1a,r2a) in [
    ("M1 GPT-4o",(RATER1_M1,RATER2_M1)),
    ("M2 Claude Sonnet 4.6",(RATER1_M2,RATER2_M2)),
    ("M3 Gemini 1.5 Pro",(RATER1_M3,RATER2_M3)),
    ("M4 Llama 3.3 70B",(RATER1_M4,RATER2_M4)),
]:
    v=36
    print(f"  {name:<25}  C3:R1={r1a[v,2]} R2={r2a[v,2]} | "
```

```
          f"C6:R1={r1a[v,5]} R2={r2a[v,5]} | "
          f"Tot:R1={int(r1a[v].sum())} R2={int(r2a[v].sum())}")
print("\n" + "="*65)
print(f"Pooled ICC(2,1) = {icc_p:.3f} [{lo_p:.3f}, {hi_p:.3f}] p={p_p:.4f}")
print("="*65)
```
