# Supplementary material for: Systematic Scoring Differences in Large Language Model–Based Clinical Benchmarking Reveal Evaluator-Dependent Divergence: Controlled Evaluation Study
Source: JMIR AI. 2026 Oct 26;5:e103962. doi: 10.2196/103962 (PMC13705216; doi:10.2196/103962)
Supplement: Supplementary file 2 [file ai-v5-e103962-s001.docx]

**SUPPLEMENTARY TABLE S5**

**Gold Standard: Consensus Expert Answers for All 40 Clinical Vignettes**

*When AI Grades AI | Dinc R, Ardic N | INVAMED / Med-International UK | May 2026*

These consensus expert answers were developed by the clinical authors based on current society guidelines at the time of vignette development (January–March 2026) and are presented here to document the pre-specified correct responses underpinning rubric criteria C1, C2, C3, and C6. They represent clinical positions supported by named guideline documents at Class/Level of evidence grade where available. Where genuine clinical equipoise exists, this is explicitly noted and C1=1 is the maximum score ceiling.

**Column definitions:**

- C1 Primary answer — The specific device, technique, or management decision that scores C1=2
- C1=2 requires — Minimum elements that must be present in a model response for C1=2 to be awarded
- C2 anatomical requirement — Device-anatomy matching detail required for C2=2
- C3 mandatory contraindication — Safety item that must be recognised for C3=2; classified as OBJ (objectively verifiable, not opinion-dependent) or CJ (requires clinical judgment)
- C6 Reflection trigger — Clinical uncertainty, alternative strategy, or evidence gap that defines C6=2 for this vignette
- Primary guideline source — Named guideline document anchoring the C1 answer

**★ = Sentinel vignette (V-NEURO-05). Pre-designated internal validity anchor before any model was queried.**

# Table S5. Gold Standard Consensus Expert Answers — All 40 Vignettes

*Landscape layout. Prepared prior to model querying; locked before evaluation. Abbreviations: DCB=drug-coated balloon; DES=drug-eluting stent; EVAR=endovascular aortic repair; FEVAR=fenestrated EVAR; EPD=embolic protection device; BAC=balloon-assisted coiling; CAS=carotid artery stenting; EPD=embolic protection device; SET=supervised exercise therapy; DAPT=dual antiplatelet therapy; RFA=radiofrequency ablation; OBJ=objectively verifiable; CJ=clinical judgment.*

| **Vignette** | **C1: Primary correct answer** | **C1=2 requires** | **C2: Key anatomical requirement** | **C3: Mandatory contraindication** | **C6: Reflection trigger** | **Primary guideline source** |
| --- | --- | --- | --- | --- | --- | --- |
| **DOMAIN 1 — Peripheral Artery Disease (PAD)** | | | | | | |
| **V-PAD-01** | DCB-first for TASC IIC SFA lesion (18cm). Supera interwoven nitinol stent for provisional/bailout only. SET as mandatory co-intervention. | DCB-first stated as primary; SET (supervised exercise) mentioned as Class I/A co-intervention; adductor canal 'no-stent zone' identified for mid-SFA segment | Adductor canal biomechanics: no permanent stenting in adductor canal zone (fracture risk); DCB 1:1 vessel sizing; co-axial crossing technique for chronic total occlusion | Antiplatelet pre-loading: patient on no antiplatelet — DAPT loading (aspirin 300mg + clopidogrel 300mg) mandatory before procedure. Classification: OBJ  *Paclitaxel mortality signal (Katsanos meta-analysis): acknowledge uncertainty and note current ESC IIa/B recommendation stands pending further data. Alternative: DES if DCB fails at 3 months.* | ESC/ESVS PAD Guidelines 2024. DCB: Class IIa, Level B. SET: Class I, Level A. (Aboyans et al.) |  |
| **V-PAD-02** | Angiosome-directed posterior tibial (PT) artery revascularisation for heel wound. SAFARI retrograde access if antegrade fails. CO2 angiography primary contrast strategy. | PT artery named as target (not generic 'tibial'); angiosome principle stated; ESRD contrast protocol (CO2 + ≤80mL isosmolar if supplemental); same-day dialysis scheduling | BTK vessel sizing; Mönckeberg's calcification management (lithotripsy/atherectomy); retrograde SAFARI access technique; ABI unreliability in ESRD noted | Uraemic platelet dysfunction: no antiplatelet loading without haematology input; SGLT-2 inhibitor contraindicated in ESRD (eGFR<30). Classification: OBJ  *BEST-CLI/BASIL-2 equipoise for open vs. endovascular CLTI revascularisation: acknowledge that no definitive RCT evidence favours endovascular over bypass for ESRD+CLTI. Contingency: vascular surgery review if endovascular fails.* | ESVS CLTI Guidelines 2024; BEST-CLI (Farber 2022); BASIL-2 (Bradbury 2023) |  |
| **V-PAD-03** | Bilateral SFA disease: DCB-first both limbs. Left SFA — adductor canal no-stent zone — DCB only, Supera as planned contingency if recoil. Right SFA — DCB-first, Supera provisional. Staged bilateral approach. | Adductor canal no-stent zone explicitly named for left SFA; DCB-first for both limbs stated; staged vs. simultaneous bilateral decision addressed; occupational context (postman) acknowledged | Adductor canal biomechanics; bilateral access strategy; Supera sizing (1:1 vessel diameter); POBA pre-dilatation before DCB | No antiplatelet on board: DAPT loading before procedure mandatory; smoking cessation as Class I co-intervention. Classification: OBJ  *Simultaneous vs staged bilateral SFA intervention: evidence extrapolated from single-limb trials only. Acknowledge equipoise. Contingency: if DCB fails at adductor canal, Supera stent planned rather than bare-metal stent.* | ESC/ESVS PAD 2024; SUPERB trial (Lammer 2011); IN.PACT SFA (Laird 2015) |  |
| **V-PAD-04** | Tosaka Class III diffuse SFA ISR: laser atherectomy debulking BEFORE DCB application. Not DCB alone. | Tosaka III classification stated; laser atherectomy or scoring balloon debulking step explicitly included before DCB; rationale for debulking-first given | Laser atherectomy catheter sizing; DCB after debulking (not as standalone); stent fracture exclusion assessment by fluoroscopy | DAPT duration extension post-DCB in ISR-in-DES: minimum 3 months; check prior antiplatelet status. Classification: OBJ  *Laser atherectomy + DCB vs DCB alone for Tosaka III: EXCITE ISR and PATENT trials support combination but head-to-head vs. plain DCB in Class III is limited. Acknowledge that bypass may be required if endovascular Tosaka III DCB fails.* | EXCITE ISR trial (Dippel 2015); ESVS PAD Guidelines 2024; Tosaka classification (Tosaka 2012) |  |
| **V-PAD-05** | CERAB technique (covered endovascular reconstruction of aortic bifurcation) with covered balloon-expandable stents. Not bare kissing stents. IIA preservation. | CERAB technique named (not generic 'kissing stents'); covered balloon-expandable stents (VBX, Advanta V12, or BeGraft) specified; IIA preservation principle stated; thrombophilia screen recommended for young patient without conventional risk factors | Covered BES sizing (aortic component 12–14mm, iliac 8–10mm); bilateral access via femoral; IIA origin preservation technique; fluoroscopic landing zone | IIA preservation to prevent buttock claudication/impotence; thrombophilia screen (Factor V Leiden, prothrombin 20210A, protein C/S, antithrombin) in young patient with no conventional risk factors. Classification: CJ (thrombophilia screen), OBJ (IIA preservation)  *CERAB vs. open aortobifemoral bypass (ABF) for TASC IID in young patient (<50): ABF has superior long-term patency (15-year data). Acknowledge this is the formal equipoise. Endovascular CERAB preferred first-line but open bypass remains standard for young patients.* | ESVS Aortoiliac Guidelines 2023; CERAB technique (Goverde 2013); TASC II classification |  |
| **V-PAD-06** | DCB-first for SFA CLTI in frail patient. Local anaesthesia under MAC. Rivaroxaban 48h hold perioperatively. Heparin-bonded covered stent (Viabahn with heparin coating, HAPII) as alternative for frail patients with rest pain. | MAC/local anaesthesia recommended (not GA) for frailty; rivaroxaban 48h hold with specific eGFR note; frailty-adjusted goal-of-care discussion; DCB or heparin-bonded covered stent for long-segment CLTI | No rivaroxaban on day of procedure; eGFR-adjusted hold; access site LA; frailty score (CFS 5) documented; no BMS (outdated choice) | Rivaroxaban peri-procedural: 48h hold, not 'adjust as necessary'. COMPASS/PIONEER low-dose regimen post-procedure for CLTI. BMS contraindicated as primary strategy — outdated. Classification: OBJ (rivaroxaban hold); CJ (goals of care)  *Procedural risk vs. palliative wound care in CFS5: formal equipoise. Goals-of-care discussion with patient and family mandatory before intervention. Acknowledge that conservative wound management may be equivalent in very frail patients.* | ESVS CLTI 2024; COMPASS trial (Bosch 2017); PIONEER-AF PAD; CFS frailty scale (Rockwood) |  |
| **V-PAD-07** | DCB at failing vein graft distal anastomotic stenosis. No stenting at distal anastomosis. Contralateral CFA crossover access. | DCB preferred over stenting; biomechanical contraindication to anastomotic stenting explicitly stated (flexion point, stent fracture risk); contralateral crossover access specified; PSV ratio threshold (>3.5 or >4.0) for intervention stated | Contralateral CFA crossover access; DCB sizing to reference vessel diameter; embolic protection device consideration (limited evidence noted); PSV follow-up criteria | Anastomotic stenting: biomechanical contraindication to permanent stent at distal anastomosis — flexion-point stent fracture risk is OBJ. Note that EPD for vein graft anastomotic DCB is not established standard. Classification: OBJ  *Open surgical revision (patch angioplasty at anastomosis) vs endovascular DCB: both are valid; no RCT establishes superiority. Acknowledge this clinical equipoise explicitly. Contingency: surgical revision if DCB fails.* | ESVS Lower Limb Guidelines 2019; SVS/ACS vein graft surveillance recommendations |  |
| **V-PAD-08** | May-Thurner syndrome: pharmacomechanical CDT before stenting for acute/subacute thrombus. Dedicated venous stent (Venovo, Abre, or Vici) — NOT Wallstent or Viabahn (arterial stents). 14–16mm CIV sizing. Retrograde popliteal access for CDT. | CDT before stenting for residual acute thrombus; dedicated venous stent named (Venovo/Abre/Vici); 14–16mm CIV sizing stated; Factor V Leiden: lifelong anticoagulation plan | Retrograde popliteal vein access for CDT; dedicated venous stent sizing 14–16mm for CIV; intravascular ultrasound (IVUS) for stent sizing confirmation; fluoroscopic completion venogram | Wallstent/Viabahn are arterial stents — contraindicated in venous system for primary venous reconstruction. Dedicated venous stent mandatory. Classification: OBJ (device class); CDT before stenting for residual thrombus: OBJ  *Stenting vs anticoagulation alone for May-Thurner with Factor V Leiden: no RCT. For symptomatic MTS with DVT, stenting improves PTS-free survival but evidence from case series only. Acknowledge and present anticoagulation-alone as alternative.* | ATTRACT trial (Vedantham 2017); ESVS Venous Guidelines 2022; Venovo: Murphy 2019; Abre: Neglén |  |
| **DOMAIN 2 — Abdominal Aortic Aneurysm (AAA)** | | | | | | |
| **V-AAA-01** | Standard infrarenal EVAR for 58mm AAA, favourable anatomy. Device: Endurant IIs (Medtronic) first-line. Regional anaesthesia preferred for COPD. AS pre-op cardiac assessment. | EVAR correctly identified; device brand with IFU justification stated; 10–20% oversizing principle; mild AS: pre-op cardiac assessment (stress echo or CT-FFR) required before elective EVAR; surveillance: CTA at 1 month then duplex-dominant annually | Neck anatomy: suprarenal vs. infrarenal fixation rationale; iliac sizing; oversizing principle (10–20%); regional vs GA for COPD; annual duplex surveillance from year 2 | Mild AS: not automatically a contraindication but requires pre-op cardiac assessment. Mean gradient >20mmHg or AVA <1.0cm² warrants cardiology review before elective EVAR. Classification: CJ  *EVAR vs open repair in 74-year-old male: EVAR-1/DREAM trials show early EVAR survival advantage lost at 8–10 years due to reintervention burden. Acknowledge lifelong surveillance commitment and late conversion rate when counselling patient.* | EVAR-1 (NEJM 2010); DREAM (NEJM 2005); ESVS Aortic Guidelines 2024; Endurant IFU |  |
| **V-AAA-02** | EVAR at limit of IFU: hostile anatomy (48° neck angulation, circumferential thrombus, bilateral CIAs 8–9mm). No-touch technique for thrombus-loaded neck. Low-profile system: Ovation (14Fr) or Lombard Alto. Iliac conduit if access prevents delivery. | No-touch aortic technique for thrombus neck; low-profile system identified by name; iliac angioplasty vs. conduit decision tree; CO2+IVUS for contrast minimisation; AS haemodynamic monitoring | No-touch aortic clamping for circumferential thrombus; Ovation 14Fr or equivalent LP system; bilateral CIA 8–9mm: consider percutaneous transluminal angioplasty (PTA) or surgical iliac conduit; EndoAnchors for angulated neck | AS >moderate: haemodynamic monitoring mandatory during EVAR; pre-op stress assessment if previously unassessed. Classification: CJ  *Anatomy at IFU limit: formal acknowledgement that this anatomy may not meet IFU criteria; open repair or FEVAR/complex EVAR may offer better long-term durability. MDT discussion and patient counselling on higher reintervention risk mandatory.* | ESVS Aortic 2024; Ovation IFU; EndoAnchors (ANCHOR registry); low-profile systems review (Oderich 2020) |  |
| **V-AAA-03** | Emergency rAAA + prior open graft: REBOA Zone 3 for aortic control, then aorto-uni-iliac (AUI) + fem-fem crossover. CO2 angiography primary (contrast allergy). Bifurcated endograft if AUI not feasible. NOT custom fenestrated EVAR. | REBOA Zone 3 (not Zone 1 — suprarenal occlusion causes ischaemia); bifurcated endograft or AUI correctly identified for emergency; CO2/IVUS as primary contrast strategy; goals-of-care for CFS4 patient addressed | REBOA placement zone (Zone 3 = infrarenal); graft-in-graft technique for prior open repair; bilateral femoral cutdown; accelerated IV methylprednisolone for contrast allergy | Custom fenestrated EVAR CONTRAINDICATED in emergency rupture — 6–8 week manufacturing lead time. This is OBJ — any response recommending custom FEVAR for emergency rAAA is clinically unsafe. Classification: OBJ  *Palliative care vs. emergency EVAR in CFS4 82-year-old: formal equipoise. Emergency EVAR mortality in octogenarians with CFS4 is 30–50%. Goals-of-care discussion and advance directive review mandatory before intervention. Both emergency EVAR and palliation are legitimate options.* | IMPROVE trial (NEJM 2014); REBOA in rAAA (Stannard 2011); ESVS Aortic Emergency Guidelines 2023 |  |
| **V-AAA-04** | Type II endoleak with sac growth >5mm at 6 months: intervention indicated. Translumbar embolisation (TLE) with coil + Onyx 18 nidus filling. Primary over transarterial embolisation (TAE). | Type II endoleak correctly classified (IMA + lumbar feeders); sac growth 5mm threshold for intervention stated (ESVS Class I); TLE with coils + Onyx identified; nidus-filling principle vs. feeding-vessel-only embolisation distinguished | Translumbar access technique; nidus localisation by CT-guided puncture; Onyx 18 vs. coils; transcaval alternative if TLE not feasible; post-procedure CT at 3 months | Type II endoleak: intervention not required for stable sac. 5mm growth or >6 months persistence = Class I indication for intervention. Classification: OBJ (threshold)  *TLE vs TAE for Type II endoleak: TLE has superior technical success but similar recanalisation rates (~30%). Acknowledge that recanalisation is common with both techniques and re-intervention may be needed. Alternative: transarterial via SMA→IMA collateral if TLE not feasible.* | ESVS Aortic 2024; Type II endoleak management (Sheehan 2010); Onyx in endovascular surgery |  |
| **V-AAA-05** | 66-year-old female, 42mm AAA: NO intervention — surveillance only. Female-specific 55mm intervention threshold (or 50mm with rapid growth). Annual ultrasound. | Surveillance-only correctly identified as the correct answer; female-specific threshold stated (≥50mm or ≥55mm per society — note ESVS uses 50mm for females vs 55mm for males); growth rate monitoring; statin/ACEI/smoking cessation optimisation | No device; ultrasound surveillance 6-monthly if 40–44mm or annually if stable; CT to characterise anatomy only when intervention threshold approached | OCP: not a contraindication to surveillance but note thrombotic risk; family screening of first-degree relatives. Classification: CJ  *Female-specific rupture risk curve: at 42mm, female rupture risk is ~0.5%/year. PIVOTAL/CAESAR trial data support surveillance. However, female sex = higher rupture risk per cm than males — threshold debate (50mm vs 55mm). Acknowledge this sex-specific uncertainty.* | ESVS Aortic 2024 (female threshold 50mm); PIVOTAL trial; CAESAR trial |  |
| **V-AAA-06** | Acute complicated Type B aortic dissection (right renal malperfusion, right upper limb pulse deficit): TEVAR. Gore CTAG first-line for conformability. Anti-impulse therapy (IV labetalol, SBP 100–120, HR <60). CSF drain for SCI prophylaxis. Concurrent infrarenal AAA 53mm: defer — do not treat simultaneously. | TEVAR for complicated TBAD correctly identified; medical management for uncomplicated TBAD also stated; anti-impulse targets given; CSF drain for SCI; concurrent AAA deferred | TEVAR sizing for acute dissection (10–15% oversizing, NOT 20% as in elective); true lumen IVUS confirmation mandatory; left subclavian management; bird-beaking prevention | Concurrent 53mm AAA: do NOT treat simultaneously with TEVAR for acute dissection — physiological stress, anticoagulation, and haemodynamic instability contraindicate staged same-sitting EVAR. Classification: OBJ  *TEVAR vs optimal medical therapy for complicated TBAD: ADSORB trial (underpowered, n=61); INSTEAD-XL shows TEVAR benefit at 5 years for uncomplicated TBAD. Evidence for complicated TBAD relies on registry data, not RCTs. Acknowledge this and present OMT as alternative if malperfusion resolves.* | ADSORB (Brunkwall 2012); INSTEAD-XL (Nienaber 2013); ESVS Aortic 2024; Gore CTAG IFU |  |
| **V-AAA-07** | Juxtarenal AAA (renal arteries <10mm from neck): standard EVAR anatomically contraindicated. Fenestrated EVAR (FEVAR) with bilateral renal fenestrations. Cook Zenith Fenestrated first-line. Ch-EVAR as contingency. LVEF 45%: cardiac optimisation pre-FEVAR. | Standard EVAR contraindication stated (inadequate neck); FEVAR identified by name; bilateral renal fenestrations; LVEF 45% cardiac optimisation requirement; HbA1c 8.9% pre-optimisation; Ch-EVAR as contingency named | Fenestration alignment (scallop vs fenestration for proximal renal); Advanta V12 or iCast covered bridging stents; FEVAR manufacturing lead time (4–6 weeks); concurrent 53mm AAA staged approach | HbA1c 8.9%: pre-operative glycaemic optimisation recommended for elective FEVAR; LVEF 45% requires cardiology input and optimisation of HF therapy before elective complex EVAR. Classification: CJ  *FEVAR vs Ch-EVAR for juxtarenal: comparable short-term outcomes, FEVAR may offer better long-term branch vessel patency. Acknowledge limited RCT data. FEVAR vs open juxtarenal repair: open has excellent long-term outcomes in experienced centres; FEVAR offers lower perioperative risk in cardiorespiratory co-morbidity.* | GLOBALSTAR registry; FENIX registry; PERICLES (Ch-EVAR); ESVS 2024 |  |
| **V-AAA-08** | Juxtarenal AAA + LVEF 40% + prior CABG: FEVAR with cardiac risk optimisation. Cardiological pre-assessment mandatory. Consider Ch-EVAR if cardiac risk precludes prolonged FEVAR procedure time. | FEVAR identified for juxtarenal anatomy; LVEF 40% cardiac optimisation; prior CABG — antiplatelet/anticoagulation management; Ch-EVAR as alternative if cardiac risk high; endovascular preferred over open due to cardiac comorbidity | Same as V-AAA-07 for anatomy; additionally: CABG antiplatelet management (aspirin continuation); LVEF 40% — target MCS threshold for haemodynamic instability during prolonged FEVAR | LVEF 40% + prior CABG: formal cardiology clearance required before elective FEVAR. Cardiac decompensation during extended FEVAR is a recognised perioperative risk. Classification: OBJ (cardiology clearance); CJ (timing)  *Procedural risk vs rupture risk for 58mm AAA with LVEF 40%: EVAR-1 data excluded patients with LVEF <30%; current registry data shows acceptable outcomes with cardiac optimisation. Acknowledge individualised risk-benefit calculation with cardiac MDT.* | ESVS Aortic 2024; FEVAR with cardiac comorbidity (Antonello); LVEF threshold for EVAR |  |
| **DOMAIN 3 — Chronic Venous Insufficiency (CVI)** | | | | | | |
| **V-CVI-01** | Bilateral GSV ablation: RFA (ClosureFAST) as primary (ESVS Grade 1A). EVLA 1470nm as equivalent alternative. Staged bilateral approach: right first, left deferred 4–6 weeks. EHIT screening duplex at 3–7 days post-procedure. | RFA named as Grade 1A primary; EVLA as equivalent alternative; staged bilateral rationale stated; catheter 2cm from SFJ; EHIT classification system mentioned; 3–7 day surveillance duplex planned | Catheter positioning 2.0cm from SFJ; tumescent anaesthesia safety calculation (35mg/kg max lidocaine with adrenaline); EHIT grading (I–IV); concomitant phlebectomy vs. deferred foam sclerotherapy decision | ABI before prescribing compression stockings mandatory — compression contraindicated if ABPI <0.5. Classification: OBJ (ABI check before compression)  *RFA vs EVLA: CLASS trial shows comparable outcomes at 5 years. No superiority of RFA over EVLA in terms of GSV occlusion or QoL at 5 years. Acknowledge true equipoise between the two thermal modalities. Concomitant phlebectomy vs. staged sclerotherapy: also equipoise (PHLEBECTOMY trial).* | ESVS Venous Guidelines 2022 (Grade 1A RFA); CLASS trial (Brittenden 2014); PHLEBECTOMY trial |  |
| **V-CVI-02** | TASC IIC right CIA 7cm occlusion: covered stent + kissing stent technique (CERAB configuration). Balloon-expandable covered stents (Viabahn VBX or Fluency). DVT history: therapeutic anticoagulation pre-procedure. | Covered stent identified (not bare-metal); kissing stent / CERAB configuration; balloon-expandable for iliac; DVT history: anticoagulation management specified; contralateral CFA access for crossover technique | CERAB kissing stent technique; covered BES sizing (9–10mm CIA); aortic landing zone (12mm); bilateral femoral access; IVUS for stent sizing; post-procedure duplex ABI | Prior DVT: therapeutic anticoagulation must be continued peri-procedurally (not stopped); assess thrombophilia status before intervention. Classification: OBJ (anticoagulation continuity)  *Covered vs bare-metal stent for TASC IIC CIA: COBEST trial shows superior primary patency with covered stents. Acknowledge covered stent is preferred but bare-metal stent is acceptable in some anatomical configurations. Open iliac endarterectomy: alternative with excellent long-term results.* | COBEST trial (Mwipatayi 2011); TASC II classification; ESVS PAD 2024 |  |
| **V-CVI-03** | Pregnancy varicosities: CONSERVATIVE management only. Graduated compression (15–21mmHg). No thermal ablation, no sclerotherapy during pregnancy. Post-partum reassessment at 3 months. | Thermal ablation correctly contraindicated during pregnancy; graduated compression (not surgical stockings alone); walking encouraged; post-partum reassessment planned; patient counselling on natural history (majority regress post-partum) | No catheter intervention; compression sizing; elevation advice; walking programme; no sclerosant (teratogenic risk); post-partum duplex at 3 months | Thermal ablation, foam sclerotherapy, and cyanoacrylate ALL contraindicated in pregnancy. This is OBJ — no endovenous treatment during pregnancy regardless of severity. Classification: OBJ  *Post-partum natural history of pregnancy varicosities: ~30–50% regress spontaneously within 3 months post-partum. Post-partum intervention threshold (C2–C3 residual at 3 months = treat; isolated C1 = monitor). Acknowledge this natural history uncertainty when counselling.* | ESVS Venous 2022; pregnancy-specific venous management guidelines (RCOG) |  |
| **V-CVI-04** | Cyanoacrylate (VenaSeal) allergy: ABSOLUTE CONTRAINDICATION to VenaSeal. Thermal alternative: RFA (ClosureFAST) or EVLA 1470nm. EVRA trial: early ablation superior to deferred for active venous ulcer (C6). MOCA as non-thermal alternative if thermal not tolerated. | VenaSeal explicitly contraindicated due to cyanoacrylate allergy; RFA or EVLA as primary alternatives; EVRA trial cited for early ablation in C6 active ulcer; wound management alongside ablation | GSV sizing for ablation (RFA catheter sizing); EVRA protocol (early GSV ablation within 2 weeks of presentation); wound care (four-layer compression + ablation); MOCA as non-thermal alternative | Cyanoacrylate allergy: absolute contraindication to VenaSeal — anaphylaxis risk. This is OBJ, not clinical judgment. HbA1c 8.1%: wound healing may be impaired; glycaemic optimisation recommended. Classification: OBJ (VenaSeal); CJ (glycaemic)  *RFA vs EVLA for C6 venous ulcer: no head-to-head RCT in active ulcer population specifically. EVRA trial uses EVLA; CLASS trial includes both but excludes active ulcer. Acknowledge this evidence gap for the C6-specific population.* | EVRA trial (Davies 2019 NEJM); ESVS Venous 2022; VenaSeal contraindications |  |
| **V-CVI-05** | 45-year-old female, 3.1cm AAA + family history: NO intervention — surveillance only. Annual CT or ultrasound. Female threshold 50mm. Family pedigree screening (first-degree relatives). Genetic panel if connective tissue features. | Surveillance-only correctly identified; female 50mm threshold stated; annual surveillance interval given; family history → screen first-degree relatives; genetic panel (ACTA2, FBN1, COL3A1, MYH11) if Marfan/EDS features | No device; CT anatomy characterisation; annual ultrasound surveillance; OCP thrombotic risk discussion | OCP: not contraindicated for small AAA but note connective tissue influence (COL3A1 mutations). Family AAA screening mandatory for first-degree relatives. Classification: CJ  *Sex-specific rupture risk at 3.1cm in young female with FAA: annual rupture risk <0.5%. Surveillance anxiety burden vs. rupture risk: formal patient discussion. Role of aortic size index (ASI = diameter/BSA) for small female with large BSA.* | ESVS Aortic 2024; PIVOTAL/CAESAR; FAA pedigree management (Nordon 2010) |  |
| **V-CVI-06** | CLTI + ESRD + heel ulcer: angiosome-directed PT artery revascularisation for heel wound. CO2 primary contrast strategy (residual renal function preservation). Same-day dialysis. Peroneal artery preservation as 'insurance' vessel. | PT artery named as angiosome target for heel (not generic 'tibial' or 'peroneal'); CO2 + ≤80mL isosmolar supplemental if needed; same-day dialysis scheduling; peroneal artery preservation strategy | BTK vessel sizing; retrograde pedal access if antegrade fails; IVUS for calcified vessels; atherectomy for Mönckeberg's calcification; wound care plan | SGLT-2 inhibitors: contraindicated in eGFR <30 (ESRD). Contrast: CO2 as primary in ESRD — iodinated contrast risks worsening residual renal function. Classification: OBJ  *Revascularisation vs palliative wound care for ESRD + contralateral amputee + age 72: extreme comorbidity. Formal palliative care consultation before intervention. Revascularisation in ESRD has 1-year mortality >40% in this profile. Acknowledge this is genuine equipoise between revascularisation and comfort care.* | ESVS CLTI 2024; Angiosome theory (Taylor 1987); ESRD contrast management guidelines |  |
| **V-CVI-07** | Symptomatic 75% ICA stenosis + hostile neck (prior neck irradiation, high bifurcation): CAS + EPD preferred over redo-CEA. Closed-cell stent (Precise Pro Rx) or dual-layer stent (CGuard/Casper) for irregular plaque. DAPT 5 days pre-loading. | CAS identified as correct choice for hostile neck; hostile neck anatomy (prior irradiation/surgery) identified as the CAS-preference driver; EPD mandatory (FilterWire EZ or Mo.Ma for soft plaque); dual-layer or closed-cell stent; DAPT pre-loading 5 days | EPD selection: FilterWire EZ for standard, Mo.Ma proximal EPD for soft unstable plaque; stent cell design (closed-cell for irregular plaque); DAPT protocol; access route (femoral CAS); intraprocedural neurological monitoring | CAS pre-loading: aspirin 100mg + clopidogrel 75mg for minimum 5 days pre-procedure. If clopidogrel-resistant by platelet function testing, consider ticagrelor. Classification: OBJ  *CAS in previously irradiated neck: radiation-induced fibrosis increases CAS technical difficulty (increased stent apposition failure risk). Acknowledge that CAS in hostile neck has higher procedure-specific risk than de novo CAS, even though it is lower than redo-CEA. CREST-2 data limited for hostile neck subgroup.* | CREST trial (Brott 2010); ESVS Carotid 2023; EPD for CAS (Kastrup 2006) |  |
| **V-CVI-08** | CEAP C1 telangiectasias, no reflux, no truncal involvement: microsclerotherapy (polidocanol 0.5–1% liquid) ONLY. No thermal ablation (no truncal reflux). OCP thrombotic risk counselling. Realistic expectations for recurrence. | Microsclerotherapy correctly identified; thermal ablation correctly excluded (no truncal reflux); OCP thrombotic risk discussed; sclerosant concentration specified; realistic recurrence counselling | No catheter; polidocanol vs STS agent choice; no compression requirement after C1 sclerotherapy (evidence ambiguous); post-treatment sun avoidance; haemosiderin staining warning | OCP: thrombotic risk during and after sclerotherapy; consider OCP holiday peri-procedure or thromboprophylaxis discussion. Classification: CJ  *Sclerosant choice for C1 telangiectasias: polidocanol vs STD — no RCT evidence of superiority. Recurrence rates 30–50% at 2 years for both agents. Foam vs liquid for telangiectasias: liquid preferred (foam risks capillary embolisation for small vessels). Acknowledge evidence base for choice.* | ESVS Venous 2022; Polidocanol (Ecklöf); sclerotherapy technique guidelines (EuropeanForum) |  |
| **DOMAIN 4 — Coronary In-Stent Restenosis (ISR)** | | | | | | |
| **V-ISR-01** | Focal LAD ISR: OCT-guided mechanism assessment first. Neointimal hyperplasia (not underexpansion) → DCB (paclitaxel). NOT DES-in-DES as primary for focal ISR with identified mechanism. | OCT mechanism assessment drives device selection (not routine DCB without imaging); neointimal hyperplasia confirmed on OCT → DCB; MLA 2.1mm² in LAD = above functional threshold (2.5mm² = FFR-equivalent); CABG exclusion rationale stated | OCT imaging protocol; DCB sizing (1:1 vessel-to-balloon ratio); NC balloon pre-dilatation before DCB; DAPT continuation (ticagrelor) post-DCB | DAPT: patient must be on DAPT before DCB — check antiplatelet status at time of procedure. De-escalation from ticagrelor to clopidogrel after 1 month acceptable. Classification: OBJ (OCT mandatory before DCB in ISR)  *DCB vs DES-in-DES for focal LAD ISR: RIBS IV (DES-in-DES superior for Mehran I–II at 1 year), ISAR-DESIRE 3 (DCB non-inferior to DES). Genuine equipoise — both are guideline-endorsed. The OCT mechanism determines which is preferred: neointimal → DCB, underexpansion → NC balloon ± DES.* | RIBS IV (Alfonso 2012); ISAR-DESIRE 3 (Byrne 2013); OCT-guided ISR (Gonzalo 2009); ESC Myocardial Revascularisation 2023 |  |
| **V-ISR-02** | Diffuse RCA ISR (Mehran Class III): DEB or new-generation DES (drug switch). EPD mandatory for diffuse ISR (embolic protection). FFR 0.76 = haemodynamically significant. LVEF 48% — MCS threshold for haemodynamic instability. | DEB or new-generation DES both acceptable; EPD explicitly recommended for diffuse ISR (distal embolic protection, e.g. FilterWire EZ); FFR 0.76 < 0.80 = significant; LVEF 48% MCS threshold stated | EPD catheter placement distal to lesion; DEB sizing (1:1); NC balloon pre-dilatation mandatory; Mehran III classification stated; post-procedure FFR confirmation | EPD for diffuse ISR: MANDATORY — distal embolisation from diffuse neointimal tissue is the primary hazard of diffuse ISR PCI. Absence of EPD is a patient safety gap. LVEF 48% with inferior hypokinesia: MCS standby threshold if LVEF <45% or haemodynamic instability. Classification: OBJ (EPD)  *DEB vs DES-in-DES for Mehran III diffuse ISR: RIBS III (DES-in-DES superior for Mehran II–III); PEPCAD III (DEB comparable). Acknowledge this is the most contested ISR decision — both are guideline-acceptable. CABG reassessment mandatory for Mehran III with LVEF <50%.* | RIBS III (Alfonso 2010); PEPCAD III (Kufner 2016); Mehran ISR classification (Mehran 1999); ESC 2023 |  |
| **V-ISR-03** | Focal LCx OM2 ISR (2.5mm vessel): DEB preferred over DES-in-DES for small vessel — avoid additional metal layering. OCT mechanism: neointimal hyperplasia without underexpansion drives DEB. NC balloon pre-dilatation before DEB. | DEB preferred for small vessel (avoids additional metal); OCT mechanism assessment drives DEB selection; NC balloon pre-dilatation before DEB; 2.25–2.5mm DCB sizing | OCT protocol for small vessel; NC balloon pre-dilatation to achieve TIMI 3 before DEB; DEB sizing (2.25mm for 2.5mm reference); ticagrelor DAPT continuation | FFR for 2.5mm vessel: MLA >2.5mm² in LAD = no functional significance, but OM2 territory: lower threshold applies. FFR <0.80 remains the intervention threshold. Classification: OBJ (FFR threshold)  *DEB vs DES for small vessel focal ISR: small vessel ISR evidence base is predominantly from larger vessel trials extrapolated down. Acknowledge the MLA functional threshold in small vessels differs — the evidence for using OCT-MLA thresholds in OM2/2.5mm vessels is limited.* | ESC Myocardial Revascularisation 2023; Small vessel ISR (Siontis 2015); RIBS V (Alfonso 2014) |  |
| **V-ISR-04** | SVG ISR: new-generation DES (Xience/Resolute) over first-generation. EPD MANDATORY for SVG PCI. LVEF 40% declining: CABG feasibility reassessment. MCS threshold if haemodynamic instability during SVG PCI. | EPD explicitly mandated as first priority; new-generation DES over first-generation Taxus; LVEF 40% CABG reassessment; MCS standby if LVEF <35% or vessel serves large territory | EPD selection for SVG (FilterWire EZ; SpiderFX); EPD catheter sizing for SVG diameter (3.5–5mm); DES sizing; post-procedure TIMI flow assessment; no intravascular imaging distal embolisation risk | EPD for SVG PCI: MANDATORY — SAFER trial shows 42% reduction in MACE with EPD. Absence of EPD in SVG PCI is a patient safety gap. Classification: OBJ (EPD in SVG)  *DEB vs DES for SVG ISR: ISAR-CABG (new-gen DES superior to DEB in SVG-ISR). Acknowledge SVG-ISR has worse outcomes than native vessel ISR regardless of strategy. CABG redo feasibility: always reassess with current SYNTAX/EuroSCORE before SVG PCI.* | SAFER trial (Baim 2002 — EPD in SVG); ISAR-CABG (Mehilli 2011); ESC 2023; SYNTAX/EuroSCORE II |  |
| **V-ISR-05** | Focal LAD ISR with stent underexpansion on OCT: NC balloon post-dilatation to OCT reference area (8.1mm² MLA target) FIRST. Then DCB if no underexpansion residual. AERD: aspirin contraindicated — clopidogrel + ticagrelor substitute approach. | Stent underexpansion identified as mechanism; NC balloon sizing to OCT reference area 8.1mm² stated explicitly; DCB after NC balloon post-dilatation; AERD correctly managed (aspirin avoided, clopidogrel alone or + ticagrelor) | NC balloon sizing (1:1 to stent reference area by OCT); pressure inflation to 20–22 atm; post-dilatation before DCB deployment; aspirin avoidance in AERD | AERD (aspirin-exacerbated respiratory disease): aspirin absolutely contraindicated even at low dose. Clopidogrel 75mg as aspirin substitute. If strong P2Y12 needed, ticagrelor + clopidogrel (aspirin-free DAPT). Classification: OBJ  *Post-dilatation alone vs post-dilatation + DCB for underexpansion ISR: when underexpansion is the sole mechanism, NC balloon post-dilatation alone may be sufficient. DCB provides additional anti-proliferative benefit. Acknowledge this specific equipoise — no RCT comparing NC balloon alone vs NC balloon + DCB for underexpansion-mechanism ISR.* | ESC Myocardial Revascularisation 2023; AERD management (Dahlén 2015); OCT-guided PCI (Collet 2021) |  |
| **V-ISR-06** | Left main bifurcation ISR: two-stent technique (DK-CRUSH or culotte). IVUS/OCT guidance mandatory. SYNTAX score and EuroSCORE II for CABG comparison. Heart team decision. New-generation DES. | Two-stent technique named (DK-CRUSH or culotte); IVUS/OCT mandatory for left main; heart team decision documented; SYNTAX score and EuroSCORE II calculated; second-generation DES; P2Y12 potency upgrade (ticagrelor/prasugrel) discussed | IVUS/OCT assessment of LM plaque extension; DK-CRUSH vs. culotte technique selection; MV and SB wire protection; kissing balloon inflation mandatory after two-stent; POT technique | Heart team decision: MANDATORY before LM bifurcation PCI. SYNTAX ≥33: CABG preferred per guidelines. For SYNTAX <22: PCI acceptable. Classification: OBJ (heart team); CJ (SYNTAX threshold application)  *DEB vs DES for LM ISR: RIBS data for LM ISR is limited to small series. Unlike non-LM ISR, evidence specifically for LM ISR remains sparse. Acknowledge this evidence gap explicitly. CABG vs PCI redo for LM ISR: CABG remains the standard for high SYNTAX scores.* | EAPCI/EACTS Left Main Guidelines 2018; DK-CRUSH (Chen 2011); RIBS (Alfonso — LM subgroup); ESC 2023 |  |
| **V-ISR-07** | Frail demented patient (CFS 5), RCA ISR: CONSERVATIVE management PRIMARY. If intervention becomes necessary, DCB (avoids prolonged DAPT from new stent). Goals-of-care documentation mandatory before any procedure. | Conservative management as primary recommendation; DCB named as least-burden option if intervention required (avoids mandatory DAPT prolongation from new DES); goals-of-care with patient and family documented; symptom-guided rather than anatomy-guided management | No device as primary; DCB if needed (1:1 sizing, minimal DAPT burden); no new DES (triple therapy risk with dementia antiplatelet); palliative cardiology consultation | Triple therapy risk: patient on apixaban for AF — adding DAPT creates major bleeding risk in CFS5 demented patient. This is OBJ — triple therapy in CFS5/dementia is a patient safety concern. Classification: OBJ (triple therapy avoidance); CJ (conservative vs. DCB decision)  *Whether any PCI is appropriate for CFS5 severe dementia: formal ethical and clinical question. Goals-of-care discussion should address whether the patient can consent, whether family/advance directive exists, and whether symptom burden justifies risk. Acknowledge this as a patient autonomy and clinical ethics question.* | ESC Heart Failure 2023; Palliative cardiology (Allen 2012); DAPT in frailty; apixaban + DAPT risk data |  |
| **V-ISR-08** | Primary PCI for STEMI post-ROSC (cardiac arrest): immediate angiography. EES (everolimus-eluting stent, Xience/Synergy) for culprit lesion. No routine non-culprit PCI at primary procedure. DAPT 12 months. FH genetic testing. Hypothermia management (TTM2 protocol — normothermia preferred). | EES sized correctly (3.5×32mm); STEMI = immediate PCI even post-ROSC (vs. TOMAHAWK: out-of-hospital cardiac arrest WITHOUT STEMI = selective angiography); thrombus aspiration: selective per TOTAL trial; FH genetic screening; DAPT 12 months | Stent sizing to proximal-distal mean; no routine aspiration (TOTAL/TAPAS); radial access if haemodynamically stable; IABP/Impella only if cardiogenic shock; intracoronary imaging for stent optimisation | Post-ROSC STEMI: immediate angiography regardless of neurological status (if STEMI on ECG). TTM2 trial: targeted temperature management — normothermia (37°C) equivalent to 33°C hypothermia. No routine hypothermia. Classification: OBJ (immediate angiography for STEMI post-ROSC; TTM2 normothermia)  *TTM2 trial (Dankiewicz 2021 NEJM): normothermia equivalent to 33°C — this changes antiplatelet management (hypothermia impairs P2Y12 absorption). Acknowledge TTM2 impact on antiplatelet pharmacokinetics post-arrest. No RCT for optimal P2Y12 agent in post-arrest STEMI.* | TTM2 trial (Dankiewicz 2021); TOTAL trial (Jolly 2015); TOMAHAWK (Thiele 2021); ESC STEMI 2023 |  |
| **DOMAIN 5 — Cerebrovascular / Neuroendovascular (NEURO)** | | | | | | |
| **V-NEURO-01** | PComA 7mm unruptured intracranial aneurysm (UIA), 3.5mm neck: BAC (balloon-assisted coiling) as primary technique. SAC (stent-assisted coiling) as escalation if BAC fails. Flow diverter: NOT indicated for posterior communicating artery saccular aneurysm. PHASES score 4: moderate risk. | BAC as primary (avoids mandatory DAPT of SAC); SAC as escalation, not primary; flow diverter correctly excluded for posterior communicating artery saccular morphology; PHASES 4 calculated; DAPT pre-loading only if SAC chosen (not mandatory for BAC alone) | BAC technique (compliant balloon sized to neck width); coil sizing; DAPT pre-loading schedule if SAC chosen (aspirin 100mg + clopidogrel 75mg × 5 days minimum); PHASES score calculation | DAPT pre-loading: MANDATORY only if stent-assisted coiling chosen. NOT mandatory for balloon-assisted coiling alone. Misapplication of this rule (requiring DAPT for BAC) is a clinical error. Classification: OBJ  *Observation vs. intervention for PHASES 4 PComA 7mm: ISUIA data shows ~2.5% 5-year rupture risk for 7mm PComA. Procedural risk of coiling ~2–3%. This is near-equipoise — observation is a legitimate alternative. Formal patient discussion of 5-year risk vs. procedural risk mandatory.* | ISUIA (Wiebers 2003); PHASES score (Greving 2014); ESVS/EANS UIA Guidelines 2022; PComA aneurysm management |  |
| **V-NEURO-02** | LVO MCA M1 (NIHSS 14, ASPECTS 8): IV alteplase bridging (0.9mg/kg, max 90mg) within 4.5h + thrombectomy. Stent retriever (Solitaire/Trevo) ± ADAPT combination. BP <185/110mmHg before thrombolysis. AF screening post-procedure. | IV tPA bridging if within 4.5h and no contraindications; stent retriever + direct aspiration (ADAPT) combination; ASPECTS 8 = good candidate; BP targets stated; etiology workup (AF screen, echo) planned; DAWN/DEFUSE-3 extended window criteria noted | Proximal guide catheter + intermediate catheter for thrombectomy; stent retriever sizing; BP management; TICI score target (≥2b); post-procedure imaging for haemorrhagic transformation | IV tPA contraindications: anticoagulation, prior ICH, recent surgery, uncontrolled hypertension — check all before administering. BP >185/110mmHg: treat before tPA. Classification: OBJ  *Bridging tPA vs. direct thrombectomy (no tPA): MR CLEAN-NO IV and DIRECT-MT trials show no significant difference in large vessel occlusion. This is now a genuine therapeutic equipoise. European guidelines still recommend bridging tPA if within window, but acknowledge the evidence for direct thrombectomy.* | MR CLEAN-NO IV (LeCouteulx 2021); DIRECT-MT (Yang 2021); DAWN (Nogueira 2018); DEFUSE-3 (Albers 2018); ESC Stroke Guidelines 2021 |  |
| **V-NEURO-03** | Symptomatic 82% ICA stenosis, TIA, hostile neck (prior neck dissection, irradiation): CAS + EPD within 14 days of TIA. FilterWire EZ or Mo.Ma proximal EPD for soft plaque. Closed-cell or dual-layer stent (Precise Pro Rx, CGuard). | CAS for hostile neck correctly identified; urgency within 14 days stated; EPD mandatory; stent design selected based on plaque morphology; DAPT pre-loading 5 days before CAS | Same EPD/stent selection as V-CVI-07; additionally: 14-day intervention window (FASTER/EXPRESS principle); aspirin 300mg + clopidogrel 300mg loading if acute presentation | CAS pre-loading: aspirin + clopidogrel minimum 5 days (or loading dose if acute). Clopidogrel resistance testing if time permits. Classification: OBJ  *CAS vs CEA in symptomatic stenosis >70%: CREST shows CAS has higher peri-procedural stroke in patients >70 years. This patient-hostile neck makes CEA high-risk, but CAS in irradiated neck carries specific technical risks (fibrous tissue, ICA tortuosity). Acknowledge bilateral CAS risks in hostile neck.* | CREST trial (Brott 2010); ESC/ESVS Carotid 2023; FASTER trial (Kennedy 2007) |  |
| **V-NEURO-04** | Ruptured AComA 9mm, wide neck (4.5mm), acute SAH Fisher 3: BAC (balloon-assisted coiling) as primary adjunct. DAPT-free coiling approach. WEB device as alternative (DAPT-free). Flow diverter/SAC CONTRAINDICATED in acute SAH (mandatory DAPT). Nimodipine vasospasm prophylaxis. OCP discontinuation. | BAC as primary (avoids mandatory DAPT); WEB device as alternative (also DAPT-free for wide-neck); SAC/flow diverter correctly excluded for acute SAH; nimodipine 60mg 4-hourly explicitly included; OCP discontinued | BAC technique for wide-neck (4.5mm); WEB sizing (5mm WEB for 4.5mm neck); coil selection for irregular morphology with daughter sac; post-procedure nimodipine; vasospasm monitoring TCD/angiography at day 7 | SAC and flow diverter CONTRAINDICATED in acute SAH: mandatory DAPT in the setting of acute SAH creates catastrophic rebleeding risk. OBJ. Nimodipine: OBJ — mandatory for SAH vasospasm prophylaxis (GRADE A, all guidelines). Classification: OBJ (both)  *Coiling vs clipping for ruptured AComA 9mm irregular: ISAT trial favours coiling for ruptured anterior circulation aneurysms. Post-ISAT data: clipping provides better aneurysm occlusion for wide-neck irregular morphology. Acknowledge that surgical clipping remains a valid alternative, especially for irregular wide-neck AComA.* | ISAT (Molyneux 2002 Lancet); BRAT trial (McDougall 2012); Nimodipine in SAH (Pickard 1989); ESVS Neurovascular 2022 |  |
| **V-NEURO-05** | Fusiform 12mm MCA M1 aneurysm + 3 weeks post-ICH: CANNOT place flow diverter immediately — DAPT contraindicated post-ICH. PRIMARY: surgical EC-IC bypass + aneurysm trapping (avoids DAPT). ALTERNATIVE: deferred PED at 3–6 months with staged antiplatelet re-introduction. PHASES 8 = ~15% 5-year rupture risk justifies intervention.  **★ SENTINEL VIGNETTE: C3=0 if DAPT contraindication absent (M4 Llama scored C3=0 by both raters — only such score in dataset). C6=2 only if formal alternative strategy articulated with evidence limitation acknowledged.** | DAPT contraindication in 3-week post-ICH patient explicitly identified as the central safety issue; EC-IC bypass + trapping named as primary DAPT-free strategy; PED deferred 3–6 months as alternative; staged antiplatelet timing protocol; MDT documentation; PHASES 8 calculated; BP control as immediate priority | EC-IC bypass assessment (STA-MCA bypass anatomy); balloon occlusion test (BOT) before trapping; PED sizing for fusiform morphology; antiplatelet re-introduction protocol (aspirin from week 6, add clopidogrel from month 3); M2 branch preservation assessment | DAPT in 3-week post-ICH patient: ABSOLUTELY CONTRAINDICATED. This is the only objectively verifiable contraindication in the entire dataset — no clinical judgment involved. Any response that does not identify this receives C3=0.  *Classification: OBJ *** SENTINEL ITEM — pre-designated internal validity anchor* | EC-IC bypass + trapping vs deferred PED: no RCT directly comparing these strategies for fusiform MCA aneurysm post-ICH. Evidence comes from case series only. The timing of antiplatelet re-introduction post-ICH (safe window: 4-8 weeks) is based on expert consensus, not RCT data. This specific evidence gap MUST be acknowledged for C6=2. | *PHASES score (Greving 2014); Pipeline for fusiform aneurysms (Nelson 2011); EC-IC bypass (Lawton 2011); Antiplatelet re-introduction post-ICH (Biffi 2019); ESC/ESVS Neurovascular 2022* |
| **V-NEURO-06** | Asymptomatic 72% post-CEA ICA restenosis: CONSERVATIVE management primary (surveillance + BMT). CAS threshold for restenosis = symptomatic OR ≥80% asymptomatic. At 72% asymptomatic, intervention NOT indicated. | Conservative management correctly identified as primary; CAS threshold for post-CEA restenosis ≥80% asymptomatic or any symptomatic; 72% asymptomatic = below intervention threshold; duplex surveillance interval stated; BMT optimisation | No catheter intervention at 72% asymptomatic; 6-monthly duplex surveillance; dual-layer stent selection (CGuard/Casper) if CAS eventually needed (post-CEA fibrous tissue); COPD: local anaesthesia for CAS if intervention threshold reached | CAS pre-loading: not required at this stage (no intervention indicated). If intervention becomes needed at threshold breach, same pre-loading protocol as V-CVI-07. Classification: OBJ (72% asymptomatic = no intervention)  *CREST-2 trial: optimal medical therapy alone equivalent to CAS for asymptomatic ICA stenosis in contemporary medical era. Acknowledge that the evidence for CAS vs. BMT for asymptomatic post-CEA restenosis is limited and increasingly challenged by CREST-2 data.* | CREST-2 (Howard 2023); ESVS Carotid 2023; Post-CEA restenosis management (De Borst 2012) |  |
| **V-NEURO-07** | Borden Type I dAVF (no cortical venous drainage, no varix): OBSERVATION — no intervention indicated. Annual MRI/DSA surveillance. Spontaneous regression rate 10–20%. Conditional Onyx/MMA embolisation if Borden upgrade occurs. | Observation correctly identified; Borden Type I = no haemorrhagic risk stated (0% annual haemorrhage rate); spontaneous regression acknowledged; annual MRI/DSA planned; conditional embolisation strategy for Borden upgrade described; MMA danger zones identified if embolisation considered | No catheter intervention; MRI surveillance protocol; clinical triggers for re-classification (new neurological symptoms, pulsatile tinnitus progression); DSA if MRI suggests progression; Onyx 18 via MMA if embolisation becomes needed | Borden Type I: NO intervention — haemorrhage risk is 0% annually. Intervention for Type I is not guideline-endorsed. MMA embolisation danger zones: petrous branch (facial nerve), meningo-ophthalmic (vision). Classification: OBJ (no intervention for Borden Type I)  *Observation vs. embolisation for Type I dAVF with pulsatile tinnitus: for Type I presenting with debilitating tinnitus, embolisation can be considered as quality-of-life intervention (not haemorrhage prevention). Acknowledge this tinnitus-indication threshold as a legitimate but evidence-limited indication for Type I intervention.* | Borden classification (Borden 1995); ESVS Neurovascular 2022; dAVF natural history (Zipfel 2009); Onyx embolisation for dAVF |  |
| **V-NEURO-08** | Vertebral artery origin stenosis (VAOS) 87% symptomatic, refractory to DAPT: VAST trial recommends BMT-first. Stenting for BMT failure only. If stenting: balloon-expandable stent (BES, Express Vascular SD) for flush V1 ostial lesion. Radial access preferred. | BMT-first (VAST trial) explicitly cited as basis for recommendation; BES for flush V1 ostial (not SE stent — cannot be positioned accurately at ostium); radial access; statin intensification; stenting only for DAPT-failure definition met (symptom recurrence on DAPT after 3 months) | BES sizing for V1 ostium (5×12mm Express Vascular SD or equivalent); radial access; landing zone at ostium fluoroscopy; DAPT 3 months post-stenting; statin to rosuvastatin 40mg | VAST trial (Markus 2023 NEJM): BMT not inferior to stenting for VAOS. BMT-first is now guideline-concordant. Stenting is reserved for BMT failure. Classification: OBJ (VAST trial changes management)  *VAST trial power limitation for high-grade (>80%) symptomatic stenosis subgroup: n=182 total, powered for overall comparison. The subgroup of patients with >80% stenosis on maximal DAPT remains understudied. Acknowledge that the stenting-for-refractory-symptoms decision in this specific subgroup has limited RCT evidence.* | VAST trial (Markus 2023 NEJM); CAVATAS-VA (Coward 2007); ESVS Carotid 2023; Express Vascular SD IFU |  |

# Methodological Note on Gold Standard Preparation

The consensus expert answers documented in Table S5 were prepared by the clinical authors (R.D., N.A.) based on current society guidelines in effect at the time of vignette development (January–March 2026). They represent the clinical positions against which rubric criteria C1, C2, C3, and C6 were applied during scoring.

## Criteria for 'Objectively Verifiable' vs 'Clinical Judgment' Classification

C3 items classified as OBJ (objectively verifiable) meet all three of the following: (1) the correct answer is directly stated in a named guideline document or established pharmacological fact; (2) reasonable clinicians with appropriate training would not differ on the correct answer; (3) the item can be verified by an independent reviewer without clinical specialty training. Items classified as CJ (clinical judgment) involve legitimate clinical equipoise where reasonable specialists may differ.

## Sentinel Vignette Pre-Registration

V-NEURO-05 was designated as the internal validity anchor before any model was queried. The selection criterion was: the vignette must contain at least one C3 item that is OBJ (objectively verifiable without clinical opinion) AND that requires C6 Reflection (because the correct answer is not a single device but a strategy tree requiring formal uncertainty acknowledgement). V-NEURO-05 satisfies both criteria: DAPT-in-post-ICH is OBJ, and the correct management is a strategy tree, not a device name.

## Guideline Currency

All guideline citations refer to the most current versions available at time of vignette development. ESC guidelines updated 2023–2024 were used throughout. ESVS guidelines (Aortic 2024, PAD 2024, CLTI 2024, Venous 2022, Carotid 2023, Neurovascular 2022) were the primary references for peripheral vascular and cerebrovascular domains. ACC/AHA guidelines were referenced for coronary ISR (ESC Myocardial Revascularisation 2023).

*Supplementary Table S5 | Gold Standard Consensus Expert Answers | Dinc R, Ardic N | INVAMED / Med-International UK | May 2026*
