# Supplementary material for: Systematic Scoring Differences in Large Language Model–Based Clinical Benchmarking Reveal Evaluator-Dependent Divergence: Controlled Evaluation Study
Source: JMIR AI. 2026 Oct 26;5:e103962. doi: 10.2196/103962 (PMC13705216; doi:10.2196/103962)
Supplement: Supplementary file 3 [file ai-v5-e103962-s002.docx]

**SUPPLEMENTARY FILE S2**

**Clinical Vignettes — Complete Set (40 Vignettes)**

**When AI Grades AI: Systematic Scoring Differences in LLM-Based Clinical Benchmarking Reveal Evaluator-Dependent Divergence**

*Dinc R, Ardic N | INVAMED / Med-International UK | May 2026*

| **Document role** | Supplementary File S2 — companion to main manuscript |
| --- | --- |
| **Total vignettes** | 40 vignettes (8 per domain × 5 domains) |
| **Domains** | PAD (V-PAD-01–08) \| AAA (V-AAA-01–08) \| CVI (V-CVI-01–08) \| ISR (V-ISR-01–08) \| NEURO (V-NEURO-01–08) |
| **Models evaluated** | M1: GPT-4o \| M2: Claude Sonnet 4.6 \| M3: Gemini 1.5 Pro \| M4: Llama-3.3-70B-Instruct-Turbo |
| **Raters** | Rater 1: Claude Sonnet 4.6 \| Rater 2: GPT-4o (both independent; identities intentionally disclosed) |
| **Sentinel vignette** | **★ V-NEURO-05 — Pre-designated internal validity anchor. Contains objectively verifiable contraindication (DAPT post-ICH).** |
| **Gold-standard answers** | Consensus expert answers prepared prior to model querying; locked before evaluation. Full gold-standard answers documented in Supplementary Table S5. |

*Vignettes were submitted verbatim to all four models in a single session per model via each platform's standard chat interface. No system prompting, few-shot examples, or retrieval augmentation was applied. ★ marks V-NEURO-05, the pre-designated sentinel vignette.*

# Clinical Vignettes — Complete Set

**Domain: Peripheral Artery Disease (PAD) — Vignettes V-PAD-01 to V-PAD-08**

| **Vignette 1 of 8 \| V-PAD-01** |  |
| --- | --- |
| **Patient** | 68-year-old male. Hypertension (on amlodipine 10 mg/day), type 2 diabetes mellitus (metformin 1,000 mg twice daily, HbA1c 8.2%), dyslipidaemia (rosuvastatin 20 mg/day), current smoker (40 pack-years). eGFR 62 mL/min/1.73m². |
| **Presentation** | Lifestyle-limiting intermittent claudication of the right calf for 9 months (Fontaine Stage IIb). Maximum pain-free walking distance: 80 metres on level ground. No rest pain. No tissue loss. |
| **Investigations** | ABI right 0.54, left 0.81. Duplex ultrasound: long-segment (18 cm) occlusion of the right superficial femoral artery (SFA) from its origin to the adductor canal. Popliteal artery patent. Runoff vessels (anterior tibial, posterior tibial, peroneal) all patent. CT angiography confirms TASC II Class C lesion. |
| **Medications** | Amlodipine 10 mg/day, metformin 1,000 mg twice daily, rosuvastatin 20 mg/day. No antiplatelet therapy currently prescribed. |
| **Task** | What is the most appropriate endovascular device strategy for this patient? Provide a step-by-step clinical rationale including: (1) indication for intervention, (2) device selection, (3) adjunctive pharmacotherapy, and (4) post-procedural follow-up plan. |

| **Vignette 2 of 8 \| V-PAD-02** |  |
| --- | --- |
| **Patient** | 72-year-old female. Type 2 diabetes (insulin-dependent, HbA1c 9.1%), end-stage renal disease (eGFR 14 mL/min/1.73m², on haemodialysis three times per week), hypertension, prior left below-knee amputation 3 years ago (contralateral limb ischaemia). BMI 24.1 kg/m². |
| **Presentation** | Chronic limb-threatening ischaemia (CLTI) of the right lower limb. Ischaemic rest pain for 6 weeks, non-healing heel ulcer (2 cm x 1.5 cm, Wagner Grade 2). Fontaine Stage IV. ABI right 0.31. Tissue loss threatening limb viability. |
| **Investigations** | CT angiography: severe atherosclerotic disease. Right superficial femoral artery (SFA) patent but severely diseased. Popliteal artery 60% stenosis. Tibial vessels: anterior tibial artery occluded; posterior tibial artery patent with 70% stenosis at origin; peroneal artery patent throughout. Wound swab: polymicrobial colonisation, no deep infection on MRI. |
| **Medications** | Insulin (basal-bolus regimen), amlodipine 10 mg/day, aspirin 100 mg/day. No anticoagulation. No clopidogrel. |
| **Task** | What is the most appropriate endovascular device strategy for this patient? Provide step-by-step rationale covering: (1) indication and urgency classification, (2) access and device selection for tibial revascularisation, (3) specific considerations given renal failure on haemodialysis, and (4) wound management and follow-up plan. |

| **Vignette 3 of 8 \| V-PAD-03** |  |
| --- | --- |
| **Patient** | 55-year-old male. Lifelong smoker (50 pack-years), currently smoking. No diabetes. Hypertension (bisoprolol 5 mg/day, ramipril 10 mg/day). Hypercholesterolaemia (atorvastatin 40 mg/day). eGFR 78 mL/min/1.73m². Occupation: postman, requires sustained walking. |
| **Presentation** | Bilateral claudication, right worse than left. Right calf claudication at 120 metres (Fontaine IIb). Left calf at 300 metres. Functional impairment significantly affecting employment. ABI right 0.61, left 0.74. No rest pain, no tissue loss. |
| **Investigations** | Duplex ultrasound and MR angiography: bilateral SFA disease. Right SFA: 5 cm occlusion at mid-thigh (TASC II Class B). Left SFA: 3 cm high-grade stenosis (80%) at the adductor canal. Popliteal arteries and tibial vessels patent bilaterally. No aorto-iliac disease. |
| **Medications** | Bisoprolol 5 mg/day, ramipril 10 mg/day, atorvastatin 40 mg/day. No antiplatelet therapy. |
| **Task** | What is the most appropriate management strategy? Provide rationale for: (1) whether intervention is indicated, (2) approach to the right SFA occlusion, (3) approach to the left SFA stenosis, and (4) pharmacological optimisation including antiplatelet strategy. |

| **Vignette 4 of 8 \| V-PAD-04** |  |
| --- | --- |
| **Patient** | 63-year-old male. Known peripheral artery disease with previous right SFA stent (nitinol self-expanding, 150 mm) placed 2 years ago. Type 2 diabetes (metformin 2 g/day, sitagliptin 100 mg/day, HbA1c 7.4%), dyslipidaemia. BMI 31. eGFR 59 mL/min/1.73m². |
| **Presentation** | Recurrent right calf claudication at 80 metres for 3 months (previously asymptomatic after stenting). ABI right 0.52 (was 0.78 post-stenting). No rest pain, no tissue loss. |
| **Investigations** | Duplex ultrasound: in-stent restenosis (ISR), diffuse pattern involving the entire 150 mm stent length, peak systolic velocity ratio 4.2. CT angiography confirms diffuse neointimal hyperplasia within the stent; no stent fracture identified. Outflow (popliteal, tibial) vessels patent. |
| **Medications** | Metformin 2 g/day, sitagliptin 100 mg/day, aspirin 100 mg/day, clopidogrel 75 mg/day (post-stent DAPT continued long-term), rosuvastatin 20 mg/day. |
| **Task** | What is the most appropriate endovascular strategy for managing this SFA in-stent restenosis? Provide rationale for: (1) ISR classification and mechanism, (2) device selection, (3) specific considerations given prior stent and diabetes, and (4) post-procedural management and follow-up. |

| **Vignette 5 of 8 \| V-PAD-05** |  |
| --- | --- |
| **Patient** | 48-year-old female. Non-smoker. No diabetes, no hypertension. BMI 22.3 kg/m². Occupation: marathon runner. No cardiovascular risk factors other than family history of premature PAD. eGFR 94 mL/min/1.73m². |
| **Presentation** | Progressive bilateral thigh and buttock claudication for 18 months, worse on right. Absent femoral pulses bilaterally. ABI right 0.44, left 0.51. Symptoms begin within 100 metres, limiting all exercise including professional running activity. |
| **Investigations** | CT angiography: severe bilateral aorto-iliac occlusive disease (AIOD). Right common iliac artery (CIA) occlusion from aortic bifurcation to external iliac origin. Left CIA high-grade stenosis (85%). Aortic bifurcation involved (TASC II Class D). No distal SFA disease. Kidneys normal. |
| **Medications** | No regular medications. No antiplatelet therapy. |
| **Task** | What is the most appropriate management strategy for this young patient with TASC II Class D AIOD? Provide rationale for: (1) open vs. endovascular approach decision, (2) if endovascular — device and technique selection, (3) specific considerations for a young active patient, and (4) post-procedural follow-up. |

| **Vignette 6 of 8 \| V-PAD-06** |  |
| --- | --- |
| **Patient** | 79-year-old male. Frail (Clinical Frailty Scale score 5 — mildly frail). COPD (FEV1 52%, on inhaled triple therapy). Atrial fibrillation on rivaroxaban 20 mg/day. Hypertension, type 2 diabetes (HbA1c 8.6%), previous stroke 4 years ago (no residual deficit). BMI 18.9 kg/m². eGFR 38 mL/min/1.73m². |
| **Presentation** | Ischaemic rest pain right foot for 2 weeks. ABI right 0.28. No tissue loss currently. Fontaine Stage III. |
| **Investigations** | Duplex ultrasound: right SFA total occlusion (12 cm), popliteal artery patent, two-vessel tibial runoff (posterior tibial and peroneal arteries). CT angiography confirms findings. No cardiac source of embolism identified on echocardiography (pre-existing AF, LV EF 50%). |
| **Medications** | Rivaroxaban 20 mg/day (for AF), metformin 500 mg/day, gliclazide 60 mg/day, bisoprolol 2.5 mg/day, perindopril 4 mg/day, atorvastatin 40 mg/day. |
| **Task** | What is the most appropriate endovascular strategy for this frail, high-risk patient? Provide rationale for: (1) risk stratification and treatment decision framework, (2) anticoagulation management perioperatively, (3) device selection, and (4) goals of care discussion. |

| **Vignette 7 of 8 \| V-PAD-07** |  |
| --- | --- |
| **Patient** | 61-year-old male. Known PAD with previous right femoro-popliteal bypass (autologous great saphenous vein, performed 5 years ago). Hypertension, ex-smoker (quit 2 years ago), dyslipidaemia. eGFR 71 mL/min/1.73m². No diabetes. BMI 26. |
| **Presentation** | Recurrent right calf claudication at 150 metres for 4 months. ABI right 0.57 (was 0.82 post-bypass). Prior bypass was in situ saphenous vein graft. |
| **Investigations** | Duplex ultrasound: vein graft patent but with focal high-grade stenosis (PSV ratio 4.8) at the distal anastomosis (vein-popliteal junction). No intrinsic graft disease. Outflow popliteal and tibial vessels patent. |
| **Medications** | Aspirin 100 mg/day, clopidogrel 75 mg/day, rosuvastatin 40 mg/day, perindopril 8 mg/day, amlodipine 5 mg/day. |
| **Task** | What is the most appropriate strategy for managing this graft stenosis? Provide rationale for: (1) treatment indication and urgency, (2) endovascular vs. open approach, (3) device selection if endovascular, and (4) post-procedural surveillance. |

| **Vignette 8 of 8 \| V-PAD-08** |  |
| --- | --- |
| **Patient** | 58-year-old male. Recent diagnosis of iliofemoral deep vein thrombosis (DVT) 6 weeks ago, currently on rivaroxaban 20 mg twice daily (treatment dose). No prior DVT/PE. Factor V Leiden heterozygous identified on thrombophilia screen. No diabetes, no hypertension. Non-smoker. BMI 28.4 kg/m². eGFR 82 mL/min/1.73m². |
| **Presentation** | Significant post-thrombotic syndrome (PTS) symptoms: severe left leg oedema, pain, heaviness, and venous claudication at 200 metres. CEAP C4b. Villalta PTS score 16 (severe). Profoundly limiting daily function. DVT diagnosed on duplex at initial presentation. |
| **Investigations** | CT venography: residual acute thrombus in left iliac vein. Left common iliac vein compressed by right common iliac artery at the L5 level (May-Thurner anatomy confirmed). Left external iliac vein partially recanalised. Inguinal region and femoral veins patent. |
| **Medications** | Rivaroxaban 20 mg twice daily (treatment dose, 6 weeks into planned 6-month course). No antiplatelet therapy. |
| **Task** | What is the most appropriate management strategy? Provide rationale for: (1) the role of catheter-directed thrombolysis (CDT) at this time point, (2) the role of iliac vein stenting for May-Thurner syndrome, (3) anticoagulation strategy, and (4) long-term management of Factor V Leiden. |

**Domain: Abdominal Aortic Aneurysm (AAA) — Vignettes V-AAA-01 to V-AAA-08**

| **Vignette 1 of 8 \| V-AAA-01** |  |
| --- | --- |
| **Patient** | 74-year-old male. Ex-smoker (quit 5 years ago, 35 pack-years). Well-controlled hypertension (perindopril 8 mg/day), no diabetes. BMI 27.4 kg/m². Mild COPD (FEV1 68% predicted). Cardiac history: previous percutaneous coronary intervention (PCI) for single-vessel disease 6 years ago, asymptomatic since. eGFR 71 mL/min/1.73m². |
| **Presentation** | Incidental finding of AAA on abdominal ultrasound performed during surveillance follow-up. No abdominal or back pain. Haemodynamically stable. |
| **Investigations** | CT angiography: infrarenal AAA, maximum diameter 58 mm. Aortic neck length 32 mm, neck angulation 22°, neck diameter 24 mm. Bilateral common iliac arteries 14 mm (no iliac aneurysm). No significant neck thrombus. Anatomy assessed as suitable for standard EVAR. American Society of Anaesthesiologists (ASA) class II. |
| **Medications** | Perindopril 8 mg/day, aspirin 100 mg/day, atorvastatin 40 mg/day. |
| **Task** | What is the most appropriate management strategy for this patient? Provide a step-by-step rationale covering: (1) indication for intervention, (2) open versus endovascular repair decision, (3) device/graft type selection, and (4) post-procedural surveillance protocol. |

| **Vignette 2 of 8 \| V-AAA-02** |  |
| --- | --- |
| **Patient** | 69-year-old male. Known AAA under surveillance for 3 years. Hypertension (lisinopril 10 mg/day), ex-smoker (quit 10 years ago), dyslipidaemia (atorvastatin 40 mg/day), no diabetes. eGFR 54 mL/min/1.73m². Cardiac history: mild aortic stenosis (valve area 1.4 cm², asymptomatic). BMI 29.2 kg/m². |
| **Presentation** | Routine AAA surveillance appointment. No symptoms — incidental finding at latest ultrasound. Haemodynamically stable. |
| **Investigations** | CT angiography: infrarenal AAA maximum diameter 62 mm (grew from 55 mm 12 months ago — rate 7 mm/year). Aortic neck length 18 mm, neck angulation 48 degrees, neck diameter 26 mm. Bilateral iliac access vessels: right CIA 9 mm, left CIA 8 mm — bilateral small access vessels. Neck contains moderate circumferential thrombus. Renal arteries not involved. |
| **Medications** | Lisinopril 10 mg/day, atorvastatin 40 mg/day, aspirin 100 mg/day. |
| **Task** | What is the most appropriate management strategy? Provide rationale for: (1) indication and urgency given growth rate, (2) EVAR feasibility assessment with specific anatomical challenges, (3) device-specific considerations, and (4) open surgical alternative. |

| **Vignette 3 of 8 \| V-AAA-03** |  |
| --- | --- |
| **Patient** | 82-year-old male. Frail (CFS 4 — vulnerable). Ruptured AAA presentation. Brought by ambulance. BP 80/50 mmHg on arrival, HR 118 bpm. GCS 14/15. History of previous open AAA repair 12 years ago (bifurcated Dacron graft). |
| **Presentation** | Sudden-onset severe abdominal and back pain, 2 hours duration. Pulsatile abdominal mass not palpable (previous repair). Known allergy to iodinated contrast (documented anaphylaxis requiring adrenaline). |
| **Investigations** | Emergency CT (non-contrast first): retroperitoneal haematoma, disruption of prior synthetic graft, para-aortic haematoma. Previous Dacron graft limbs visible. Contained rupture — patient relatively haemodynamically responsive. Iliac anatomy: right CIA aneurysmal (26 mm), left CIA 14 mm. |
| **Medications** | Aspirin 75 mg/day, ramipril 5 mg/day. No anticoagulation. No prior documentation of contrast allergy management protocol. |
| **Task** | What is the emergency management strategy? Provide rationale for: (1) immediate resuscitation approach, (2) contrast allergy management for emergency imaging, (3) endovascular vs. open repair decision in this specific context, and (4) device considerations. |

| **Vignette 4 of 8 \| V-AAA-04** |  |
| --- | --- |
| **Patient** | 71-year-old male. Routine EVAR surveillance CT performed 3 years post-EVAR (infrarenal bifurcated endograft, Medtronic Endurant). Asymptomatic. Hypertension, dyslipidaemia, ex-smoker. eGFR 67 mL/min/1.73m². BMI 27. |
| **Presentation** | Asymptomatic. Referred from surveillance clinic after CT demonstrated sac growth. |
| **Investigations** | CT angiography: AAA sac diameter 61 mm (was 52 mm at 1-year post-EVAR — i.e., 9 mm growth over 2 years). Endograft structurally intact; no stent fracture. Type II endoleak identified: inferior mesenteric artery (IMA) and lumbar arteries visible as blush within the sac on delayed-phase CT. No type I or III endoleak. No device migration. |
| **Medications** | Aspirin 100 mg/day, amlodipine 10 mg/day, rosuvastatin 20 mg/day. |
| **Task** | What is the most appropriate management strategy for this type II endoleak with sac growth? Provide rationale for: (1) risk classification of this endoleak, (2) treatment indication, (3) treatment options and preferred approach, and (4) follow-up protocol. |

| **Vignette 5 of 8 \| V-AAA-05** |  |
| --- | --- |
| **Patient** | 66-year-old female. Non-smoker. Hypertension (losartan 100 mg/day), well-controlled type 2 diabetes (HbA1c 6.9%, metformin 1 g twice daily). eGFR 72 mL/min/1.73m². BMI 31.4 kg/m². Incidentally detected AAA on renal ultrasound. |
| **Presentation** | Asymptomatic. No abdominal symptoms. Referred from nephrology for vascular assessment. |
| **Investigations** | CT angiography: infrarenal AAA maximum diameter 42 mm. Neck length 28 mm, neck angle 14 degrees, no significant neck thrombus. Both renal arteries arising 12 mm above aneurysm origin. Bilateral iliac arteries normal (right CIA 10 mm, left CIA 11 mm). No concurrent iliac aneurysm. No other aortic pathology. |
| **Medications** | Losartan 100 mg/day, metformin 1 g twice daily, aspirin 75 mg/day, atorvastatin 20 mg/day. |
| **Task** | What is the most appropriate management strategy? Provide rationale for: (1) treatment threshold decision, (2) recommended surveillance protocol, (3) best medical therapy optimisation, and (4) specific considerations for a female patient. |

| **Vignette 6 of 8 \| V-AAA-06** |  |
| --- | --- |
| **Patient** | 67-year-old male. Known AAA (53 mm, under 6-monthly surveillance). Admitted with acute severe chest and interscapular pain radiating to the back, 2 hours duration. BP right arm 165/90, left arm 142/85 mmHg (14 mmHg difference). Pulse deficit: right radial pulse weaker than left. No prior aortic intervention. |
| **Presentation** | Suspected acute aortic syndrome. Haemodynamically stable on arrival. No focal neurological deficit. Troponin: mildly elevated (1.2 ng/L — high-sensitivity, above 99th percentile). |
| **Investigations** | CT aortography (urgent): Type B aortic dissection (Stanford B). Intimal tear at the descending thoracic aorta, 2 cm distal to the left subclavian artery. False lumen patent and perfusing the right renal artery. True lumen compressed but all visceral vessels arise from the true lumen. Separate infrarenal AAA 53 mm, not involved in dissection. Descending thoracic aorta maximum diameter 44 mm at site of dissection. |
| **Medications** | Amlodipine 10 mg/day, bisoprolol 5 mg/day, atorvastatin 40 mg/day, aspirin 75 mg/day. |
| **Task** | What is the most appropriate management strategy for this acute Type B aortic dissection? Provide rationale for: (1) initial medical management, (2) indication for TEVAR vs. medical management, (3) management of the concurrent infrarenal AAA, and (4) long-term surveillance. |

| **Vignette 7 of 8 \| V-AAA-07** |  |
| --- | --- |
| **Patient** | 74-year-old male. Referred for opinion on complex AAA anatomy. Type 2 diabetes (HbA1c 7.8%), hypertension, ex-smoker. eGFR 61 mL/min/1.73m². BMI 28. Cardiac: previous CABG 8 years ago, LVEF 45%. |
| **Presentation** | Asymptomatic AAA detected incidentally. No abdominal symptoms. |
| **Investigations** | CT angiography: juxtarenal AAA, maximum diameter 58 mm. Aneurysm extends to within 4 mm of the right renal artery and 2 mm of the left renal artery — insufficient neck for standard infrarenal EVAR (inadequate proximal seal zone). Both renal arteries widely patent. Bilateral iliac access adequate (right CIA 12 mm, left CIA 11 mm). No visceral vessel involvement. |
| **Medications** | Metformin 1 g twice daily, ramipril 10 mg/day, aspirin 100 mg/day, bisoprolol 5 mg/day, atorvastatin 40 mg/day. |
| **Task** | What is the most appropriate management strategy for this juxtarenal AAA? Provide rationale for: (1) treatment indication, (2) why standard EVAR is insufficient, (3) endovascular alternatives, and (4) open surgical option. |

| **Vignette 8 of 8 \| V-AAA-08** |  |
| --- | --- |
| **Patient** | 45-year-old female. No cardiovascular risk factors. Non-smoker, non-diabetic, normotensive. BMI 21.2 kg/m². Referred after family history revealed two first-degree relatives with AAA (father operated at 62, paternal uncle ruptured AAA at 58). No previous abdominal imaging. |
| **Presentation** | Asymptomatic. Attending for AAA screening given family history. Abdominal aorta not palpable on examination. No abdominal symptoms. |
| **Investigations** | Abdominal ultrasound: aortic diameter 3.1 cm at infrarenal level — upper limit of normal (AAA defined as >3.0 cm). No other pathology identified. Genetic testing not yet performed. |
| **Medications** | No regular medications. Oral contraceptive pill (ethinylestradiol/levonorgestrel). |
| **Task** | What is the appropriate management for this patient? Provide rationale for: (1) whether 3.1 cm constitutes an AAA in a female patient, (2) surveillance protocol, (3) genetic investigation, and (4) specific advice regarding the oral contraceptive pill. |

**Domain: Chronic Venous Insufficiency (CVI) — Vignettes V-CVI-01 to V-CVI-08**

| **Vignette 1 of 8 \| V-CVI-01** |  |
| --- | --- |
| **Patient** | 52-year-old female. BMI 31.2 kg/m². No significant past medical history. Non-smoker. Office worker, prolonged standing 6–8 hours/day. No prior history of deep vein thrombosis (DVT). No anticoagulant or antiplatelet therapy. |
| **Presentation** | Progressive bilateral lower limb varicosities and leg heaviness for 4 years, worse on the left. C2S, Ep, As, Pr classification (CEAP). Duplex ultrasound confirms: left great saphenous vein (GSV) reflux from saphenofemoral junction (SFJ) to knee, GSV diameter at thigh 7.2 mm. Right GSV reflux limited to above-knee segment, diameter 5.8 mm. No deep venous reflux or obstruction bilaterally. |
| **Investigations** | Full-length duplex ultrasound of bilateral lower limb venous system completed (as above). Coagulation screen: normal. No contraindication to thermal ablation identified. |
| **Medications** | None regular. Currently using class II compression stockings with partial symptomatic benefit. |
| **Task** | What is the most appropriate endovascular ablation strategy for this patient? Provide rationale covering: (1) treatment indication and timing, (2) device selection and approach (left GSV), (3) management of the right GSV, and (4) post-procedural care and follow-up. |

| **Vignette 2 of 8 \| V-CVI-02** |  |
| --- | --- |
| **Patient** | 44-year-old male. BMI 34.6 kg/m². Previous DVT of the right leg 5 years ago, treated with 6 months of anticoagulation, now off therapy. No known thrombophilia. Office worker. Non-smoker. |
| **Presentation** | Progressive right leg symptoms: varicosities, oedema, skin discolouration (lipodermatosclerosis), and a healed venous ulcer (C5, healed active ulcer, CEAP). Duplex ultrasound: right great saphenous vein (GSV) reflux with 4.2-second reflux duration at SFJ; right small saphenous vein (SSV) reflux with perforator incompetence at mid-calf. Left GSV competent. |
| **Investigations** | Full bilateral venous duplex: as above. No evidence of residual deep vein thrombosis. Deep veins patent and competent bilaterally. Coagulation screen: normal. No absolute contraindication to thermal ablation identified. |
| **Medications** | No regular medications. No anticoagulation or antiplatelet therapy. |
| **Task** | What is the most appropriate management strategy? Provide rationale for: (1) treatment indication and CEAP classification, (2) management of the right GSV, (3) management of the right SSV with perforator incompetence, and (4) post-procedural compression and follow-up. |

| **Vignette 3 of 8 \| V-CVI-03** |  |
| --- | --- |
| **Patient** | 38-year-old female. 28 weeks pregnant (second pregnancy). No prior venous disease. No prior DVT. BMI 26.8 kg/m² pre-pregnancy. Non-smoker. |
| **Presentation** | New bilateral lower limb varicosities, more prominent on the left, developing during the current pregnancy. Left GSV clinically prominent from groin to knee. Significant leg heaviness and aching, worse at end of day. CEAP C2S. No skin changes, no ulceration. |
| **Investigations** | Duplex ultrasound: bilateral GSV reflux. Left GSV diameter 7.8 mm, reflux from SFJ to below-knee. Right GSV diameter 5.2 mm, reflux above-knee only. No DVT. No deep venous reflux. |
| **Medications** | Pregnancy vitamins (folic acid, vitamin D). No antiplatelet or anticoagulation. No vasoactive medications. |
| **Task** | What is the appropriate management strategy during pregnancy and in the post-partum period? Provide rationale for: (1) treatment approach during active pregnancy, (2) post-partum reassessment, (3) timing and modality of definitive treatment, and (4) thromboprophylaxis considerations. |

| **Vignette 4 of 8 \| V-CVI-04** |  |
| --- | --- |
| **Patient** | 67-year-old male. History of bilateral lower limb varicosities for 20 years, poorly managed. Hypertension, type 2 diabetes (HbA1c 8.1%), BMI 36.2 kg/m². Ex-smoker. eGFR 64 mL/min/1.73m². Known cyanoacrylate allergy (documented reaction to surgical skin adhesive containing acrylate compound). |
| **Presentation** | Active right medial malleolus venous ulcer (CEAP C6A), 4 cm x 3 cm, 14 weeks duration, not healing despite compression dressings. Duplex ultrasound: right GSV reflux 5.6 mm diameter, left GSV competent. |
| **Investigations** | Duplex ultrasound: right GSV reflux 5.6 mm diameter, left GSV competent. ABI right measurement required before applying compression. See clinical presentation for full details. |
| **Medications** | Metformin 1 g twice daily, gliclazide 80 mg twice daily, amlodipine 10 mg/day, aspirin 75 mg/day. No anticoagulation. |
| **Task** | What is the most appropriate management strategy? Provide rationale for: (1) ulcer classification and urgency, (2) device selection given the documented allergy, (3) adjuncts to aid ulcer healing, and (4) long-term prevention. |

| **Vignette 5 of 8 \| V-CVI-05** |  |
| --- | --- |
| **Patient** | 51-year-old male. Ex-rugby player, sedentary now. BMI 29.1 kg/m². No prior DVT, no anticoagulation. No significant comorbidities. Non-smoker. eGFR 88 mL/min/1.73m². |
| **Presentation** | Left leg discomfort, mild varicosities, and recurring superficial thrombophlebitis (three episodes in 2 years) in left anterior thigh varicosities. CEAP C2. No skin changes, no ulceration. Duplex confirms superficial varicosities arising from an incompetent anterior accessory great saphenous vein (AAGSV). GSV itself competent at SFJ. SSV competent. |
| **Investigations** | Duplex: incompetent left AAGSV with reflux, diameter 6.1 mm at the thigh. Left GSV competent with negative tourniquet test. No deep venous reflux. No DVT. |
| **Medications** | No regular medications. |
| **Task** | What is the most appropriate treatment strategy? Provide rationale for: (1) indication for intervention given recurrent thrombophlebitis, (2) whether to treat the AAGSV or the GSV, (3) technique selection for AAGSV ablation, and (4) management of thrombophlebitis episodes. |

| **Vignette 6 of 8 \| V-CVI-06** |  |
| --- | --- |
| **Patient** | 29-year-old female. BMI 22.4 kg/m². No prior DVT, no comorbidities. Non-smoker. Takes no regular medications. Competitive swimmer. |
| **Presentation** | Bilateral lower limb telangiectasias and reticular veins, predominantly medial thighs and calves bilaterally. No varicosities. No symptoms other than cosmetic concern. CEAP C1. Bilateral GSV competent on duplex. No reflux in any named truncal vein. |
| **Investigations** | Duplex ultrasound: no truncal reflux identified. Telangiectasias and reticular veins only — confirmed isolated CEAP C1 disease. No deep venous pathology. |
| **Medications** | No regular medications. Combined oral contraceptive pill (ethinylestradiol 30 mcg/levonorgestrel 150 mcg). |
| **Task** | What is the most appropriate management strategy? Provide rationale for: (1) appropriateness of intervention for C1 disease, (2) treatment modality selection, (3) specific considerations for this patient including the OCP, and (4) realistic outcome expectations. |

| **Vignette 7 of 8 \| V-CVI-07** |  |
| --- | --- |
| **Patient** | 58-year-old male. Morbidly obese (BMI 44.2 kg/m²). Type 2 diabetes (HbA1c 9.3%, insulin-dependent), hypertension, OSA (on CPAP). eGFR 58 mL/min/1.73m². History of bilateral hip replacements. Prior DVT left leg 8 years ago. |
| **Presentation** | Bilateral chronic venous ulcers: left medial malleolus (8 cm x 5 cm, 18 months active), right medial malleolus (3 cm x 2 cm, 6 months active). CEAP C6A bilateral. ABI bilateral 0.92. Duplex: bilateral GSV reflux, left GSV 8.4 mm, right GSV 6.7 mm. Deep veins: left — patent but post-thrombotic changes with partial reflux; right — fully competent. |
| **Investigations** | Duplex: bilateral GSV reflux, left GSV 8.4 mm, right GSV 6.7 mm. Deep veins: left — post-thrombotic changes with partial reflux; right — fully competent. ABI bilateral 0.92. See clinical presentation for full details. |
| **Medications** | Insulin (basal-bolus), metformin 2 g/day, amlodipine 10 mg/day, bisoprolol 5 mg/day, rosuvastatin 20 mg/day. No anticoagulation currently. |
| **Task** | What is the management strategy for this complex bilateral venous ulcer patient? Provide rationale for: (1) priority of treatment (which leg first and why), (2) device selection considering the technical challenges, (3) HbA1c and medical optimisation, and (4) wound management strategy. |

| **Vignette 8 of 8 \| V-CVI-08** |  |
| --- | --- |
| **Patient** | 34-year-old male. Tall, slim (BMI 20.1 kg/m², height 193 cm). No comorbidities. Non-smoker. Referred from urology after incidental finding during varicocoele assessment. |
| **Presentation** | Bilateral lower limb varicosities identified incidentally. Mild aching. CEAP C2S. Duplex: bilateral GSV reflux. Left GSV diameter 8.2 mm with reflux from SFJ to below-knee. Right GSV 4.8 mm with reflux above-knee only. Additionally, duplex demonstrates marked left-sided pelvic venous congestion with dilated left ovarian-equivalent (gonadal) vein and left renal vein compression between the aorta and superior mesenteric artery (nutcracker anatomy). |
| **Investigations** | Duplex ultrasound: left GSV incompetent AAGSV with reflux, diameter 6.1 mm at thigh. Left GSV competent. No deep venous reflux. No DVT. See clinical presentation for full details. |
| **Medications** | No regular medications. |
| **Task** | What is the most appropriate management strategy? Provide rationale for: (1) significance of the nutcracker finding, (2) appropriate investigation before any treatment, (3) management of the pelvic venous pathology, and (4) management of the lower limb varicosities. |

**Domain: Coronary In-Stent Restenosis (ISR) — Vignettes V-ISR-01 to V-ISR-08**

| **Vignette 1 of 8 \| V-ISR-01** |  |
| --- | --- |
| **Patient** | 61-year-old male. Known ischaemic heart disease. Previous PCI to proximal left anterior descending artery (LAD) with drug-eluting stent (DES, everolimus-eluting, 3.0 × 28 mm) implanted 18 months ago for stable angina. Hypertension, well-controlled. Non-diabetic. Non-smoker. LVEF 52% on most recent echocardiogram. Currently on dual antiplatelet therapy (aspirin 100 mg/day + ticagrelor 90 mg twice daily) and rosuvastatin 20 mg/day. |
| **Presentation** | Recurrent exertional chest pain (CCS class II) for 6 weeks. Exercise stress test: significant ST depression in anterior leads at 7 METs. No rest pain. No troponin elevation. |
| **Investigations** | Coronary angiography: focal in-stent restenosis at the proximal LAD stent (50–60% stenosis by visual estimate, confirmed by intravascular imaging: MLA 2.1 mm² on OCT). No other significant de novo disease. TIMI 3 flow throughout. |
| **Medications** | Aspirin 100 mg/day, ticagrelor 90 mg twice daily, rosuvastatin 20 mg/day, perindopril 5 mg/day, bisoprolol 5 mg/day. |
| **Task** | What is the most appropriate endovascular device strategy for managing this patient's in-stent restenosis? Provide rationale for: (1) mechanism assessment of ISR, (2) device selection, (3) rationale over alternative strategies (repeat stenting, CABG), and (4) post-intervention antiplatelet management. |

| **Vignette 2 of 8 \| V-ISR-02** |  |
| --- | --- |
| **Patient** | 74-year-old female. Previous acute STEMI 3 years ago treated with primary PCI to the right coronary artery (RCA) with a 3.5 x 38 mm everolimus-eluting stent (EES). Hypertension, dyslipidaemia, non-diabetic. LVEF 48% (mildly impaired, inferior wall hypokinesia). eGFR 56 mL/min/1.73m². Currently on DAPT (aspirin + clopidogrel). |
| **Presentation** | Recurrent angina, CCS class III, for 8 weeks. Stress echocardiography: new inferior wall stress-induced ischaemia, positive at low workload (4 METs). No rest symptoms, no troponin elevation. |
| **Investigations** | Coronary angiography: diffuse in-stent restenosis (ISR) throughout the entire RCA stent (Mehran Class III, diffuse). TIMI 3 flow. FFR measurement: 0.76 (haemodynamically significant, below 0.80 threshold). No significant de novo disease in other vessels. No new wall motion abnormalities on echocardiography at rest. |
| **Medications** | Aspirin 100 mg/day, clopidogrel 75 mg/day, bisoprolol 5 mg/day, ramipril 5 mg/day, atorvastatin 80 mg/day. |
| **Task** | What is the most appropriate treatment strategy for this diffuse RCA ISR? Provide rationale for: (1) FFR-confirmed significance and treatment indication, (2) device selection given diffuse ISR pattern, (3) CABG consideration, and (4) antiplatelet management. |

| **Vignette 3 of 8 \| V-ISR-03** |  |
| --- | --- |
| **Patient** | 52-year-old male. Previous PCI to left circumflex artery (LCx, second obtuse marginal branch) with a 2.5 x 20 mm sirolimus-eluting stent 9 months ago for unstable angina. Diabetic (HbA1c 7.1%, on metformin), non-smoker, hypertension. LVEF 62%. eGFR 74 mL/min/1.73m². |
| **Presentation** | Recurrent chest pain on moderate exertion (CCS class II) for 6 weeks. Exercise tolerance test: 1 mm lateral ST depression at 7 METs. No elevated troponin. |
| **Investigations** | Coronary angiography: focal ISR at the mid-stent segment of the LCx OM2 stent (50% angiographic stenosis). OCT: maximum lumen area 2.3 mm² at the site of maximum stenosis; neointimal tissue present, no stent underexpansion, no stent fracture. No neoatherosclerosis. De novo disease: mild 40% proximal LAD stenosis, not haemodynamically significant. |
| **Medications** | Metformin 1 g twice daily, aspirin 100 mg/day, ticagrelor 90 mg twice daily, amlodipine 5 mg/day, atorvastatin 40 mg/day. |
| **Task** | Provide a device selection rationale for this focal LCx ISR, including: (1) OCT-guided mechanism assessment, (2) optimal device for a 2.5 mm vessel, (3) management of the antiplatelet regimen, and (4) risk factor optimisation. |

| **Vignette 4 of 8 \| V-ISR-04** |  |
| --- | --- |
| **Patient** | 65-year-old male. Complex cardiac history: previous three-vessel CABG 15 years ago (LIMA to LAD, SVG to RCA, SVG to OM). Residual native disease. 4 years ago: PCI to the SVG to RCA with a 4.0 x 32 mm paclitaxel-eluting stent (generation 1 DES). Hypertension, dyslipidaemia, ex-smoker, non-diabetic. LVEF 40%. eGFR 52 mL/min/1.73m². |
| **Presentation** | Progressive dyspnoea on exertion over 3 months. No chest pain. BNP elevated at 340 pg/mL. Troponin: negative. Echo shows worsened inferior and lateral wall function compared with 2 years ago (LVEF was 50%). |
| **Investigations** | Coronary angiography: SVG to RCA stent: 90% diffuse ISR throughout. Native RCA: occluded (as per previous CABG indication). LIMA to LAD: patent and haemodynamically normal. SVG to OM: patent, no ISR. All other native vessels: occluded or severely diseased, non-bypassable. |
| **Medications** | Aspirin 100 mg/day, clopidogrel 75 mg/day, bisoprolol 7.5 mg/day, ramipril 10 mg/day, furosemide 40 mg/day, atorvastatin 80 mg/day. |
| **Task** | What is the most appropriate management strategy for this SVG ISR in a post-CABG patient? Provide rationale for: (1) treatment decision given LVEF and SVG anatomy, (2) PCI approach and device selection, (3) embolic protection, and (4) surgical alternative. |

| **Vignette 5 of 8 \| V-ISR-05** |  |
| --- | --- |
| **Patient** | 48-year-old male. Previous PCI to proximal LAD for stable angina with a 3.5 x 28 mm everolimus-eluting stent 6 months ago. Non-diabetic, non-smoker. Hypertension (amlodipine 5 mg/day). LVEF 62%. Known NSAID allergy (aspirin-exacerbated respiratory disease — AERD: aspirin causes bronchospasm). eGFR 88 mL/min/1.73m². |
| **Presentation** | Recurrent CCS class II angina since 4 months post-stenting. Stress MRI: inducible anteroseptal perfusion defect at moderate workload. |
| **Investigations** | Coronary angiography: focal proximal LAD ISR (70% stenosis, Mehran Class I — focal). IVUS: stent underexpansion at the ISR site (minimum stent area 5.2 mm² vs. distal reference 8.1 mm²). No neoatherosclerosis. TIMI 3 flow. |
| **Medications** | Clopidogrel 75 mg/day (substituted for aspirin due to AERD), ticagrelor 90 mg twice daily, amlodipine 5 mg/day, atorvastatin 20 mg/day. |
| **Task** | Provide a management strategy for this aspirin-intolerant patient with focal LAD ISR caused by stent underexpansion. Provide rationale for: (1) mechanism-specific treatment, (2) antiplatelet strategy without aspirin, (3) post-procedural statin intensification, and (4) long-term monitoring. |

| **Vignette 6 of 8 \| V-ISR-06** |  |
| --- | --- |
| **Patient** | 69-year-old female. Previous PCI to left main (LM) coronary artery 2 years ago with a 4.5 x 12 mm everolimus-eluting stent (distal LM + proximal LAD provisional bifurcation technique). Diabetes (HbA1c 8.4%, oral hypoglycaemics), hypertension, dyslipidaemia. LVEF 55%. eGFR 68 mL/min/1.73m². |
| **Presentation** | Deteriorating exertional dyspnoea and angina (CCS class III) for 6 weeks. Emergency admission. Troponin: mildly elevated (3x ULN, stable, no acute rise-fall pattern). ECG: no new changes. |
| **Investigations** | Urgent coronary angiography: left main ISR at the distal LM bifurcation, 80% stenosis involving the LM-LAD junction and ostium of the left circumflex (LCx). Mehran Class III (diffuse, involving bifurcation). TIMI 2 flow in the LAD territory. IVUS: adequate original stent expansion, no underexpansion. OCT: diffuse neointimal hyperplasia, no neoatherosclerosis. |
| **Medications** | Aspirin 100 mg/day, clopidogrel 75 mg/day, gliclazide 160 mg/day, empagliflozin 10 mg/day, ramipril 10 mg/day, atorvastatin 80 mg/day, bisoprolol 5 mg/day. |
| **Task** | What is the management strategy for this left main ISR with bifurcation involvement? Provide rationale for: (1) urgency and haemodynamic support consideration, (2) device selection for LM bifurcation ISR, (3) CABG versus PCI decision, and (4) antiplatelet optimisation post-procedure. |

| **Vignette 7 of 8 \| V-ISR-07** |  |
| --- | --- |
| **Patient** | 77-year-old male. Nursing home resident. Moderate dementia (MMSE 18/30). Diabetes, hypertension, atrial fibrillation (on apixaban 5 mg twice daily). LVEF 35%. eGFR 44 mL/min/1.73m². Moderate frailty (CFS 5). |
| **Presentation** | Transferred from nursing home with subacute presentation of reduced exercise tolerance and mild confusion over 3 days. No chest pain. Troponin: 2x ULN, stable. Repeat ECG: AF with inferolateral ST depression, rate 90 bpm. |
| **Investigations** | Coronary angiography performed after discussion with family and surrogate decision-maker: focal RCA ISR (80% stenosis) at previous stent (placed 3 years ago). Additional: significant diffuse LAD disease (70% mid-LAD) and obtuse marginal 60% stenosis. Global LVEF 35% (previously 40% 18 months ago). |
| **Medications** | Apixaban 5 mg twice daily, metformin 500 mg/day, gliclazide 40 mg/day, bisoprolol 2.5 mg/day, ramipril 5 mg/day, atorvastatin 20 mg/day. |
| **Task** | What is the most appropriate management strategy for this frail, demented patient with RCA ISR? Provide rationale for: (1) goals of care framework, (2) whether revascularisation is appropriate, (3) anticoagulation management if PCI is performed, and (4) medical optimisation. |

| **Vignette 8 of 8 \| V-ISR-08** |  |
| --- | --- |
| **Patient** | 42-year-old male. No cardiac history. Marathon runner. Non-diabetic, non-smoker, normotensive. BMI 23.2 kg/m². Family history: father died of MI at 54. eGFR 96 mL/min/1.73m². LDL 4.8 mmol/L (untreated). |
| **Presentation** | Sudden collapse during marathon race. Resuscitated by bystanders (3 minutes CPR). ROSC achieved before ambulance arrival. On arrival: ST elevation in V1-V4, haemodynamically stable, GCS 14. No prior cardiac investigations. |
| **Investigations** | Emergency coronary angiography: 100% acute thrombotic occlusion of the proximal LAD (culprit — STEMI). No prior stent visible. Additional finding: mild 30-40% lesions in the RCA and LCx. LVEF on bedside echo post-ROSC: 40% (anterior wall akinesia). Post-primary PCI with 3.5 x 32 mm everolimus-eluting stent to proximal LAD: TIMI 3 flow restored, residual stenosis <10%. |
| **Medications** | Aspirin 300 mg loading given pre-procedure, ticagrelor 180 mg loading. No prior medications. |
| **Task** | This vignette covers post-STEMI management rather than ISR — but provides a deliberate test of whether the model correctly identifies that this is NOT an ISR scenario. The model should: (1) recognise this is a primary PCI scenario, not ISR, (2) outline correct post-primary PCI management, (3) address secondary prevention including LDL and family history, and (4) address return-to-sport considerations. |

**Domain: Cerebrovascular / Neuroendovascular (NEURO) — Vignettes V-NEURO-01 to V-NEURO-08**

| **Vignette 1 of 8 \| V-NEURO-01** |  |
| --- | --- |
| **Patient** | 58-year-old female. Hypertension (amlodipine 5 mg/day), no diabetes, non-smoker. No prior neurological events. No anticoagulation. BMI 24.1 kg/m². Functionally independent (mRS 0). |
| **Presentation** | Incidental discovery of an unruptured intracranial aneurysm on MRI performed for investigation of headache. No subarachnoid haemorrhage. Neurological examination: normal. |
| **Investigations** | MRI/MRA and DSA: saccular aneurysm arising from the right internal carotid artery at the posterior communicating artery (PComA) junction. Maximum dome diameter 7 mm. Dome-to-neck ratio 1.8. Neck width 3.5 mm. Aspect ratio 1.6. PHASES score: 4. No daughter sac. No prior subarachnoid haemorrhage. |
| **Medications** | Amlodipine 5 mg/day. No antiplatelet therapy. |
| **Task** | What is the most appropriate management strategy for this patient's unruptured intracranial aneurysm? Provide rationale for: (1) risk stratification and treatment decision, (2) endovascular versus surgical approach, (3) device selection if endovascular treatment is chosen, and (4) follow-up protocol. |

| **Vignette 2 of 8 \| V-NEURO-02** |  |
| --- | --- |
| **Patient** | 67-year-old male. 2 hours ago: sudden-onset left-sided weakness (arm > leg), facial droop, and dysphasia. NIHSS 14 on arrival. CT head: no haemorrhage. CTA head and neck: proximal M1 MCA occlusion on the right, no carotid stenosis. ASPECTS score 8. Last known well: 90 minutes ago. |
| **Presentation** | Acute ischaemic stroke — large vessel occlusion (LVO). Within thrombolysis window and within mechanical thrombectomy window. BP 172/94 mmHg on arrival. No anticoagulation. No prior stroke. No contraindications to thrombolysis identified on initial screen. |
| **Investigations** | CT perfusion: ischaemic core 18 mL (DWI equivalent), penumbra 110 mL — significant salvageable tissue (mismatch ratio >6). CTA: right M1 MCA occlusion, good collaterals (ASPECTS 8, good collateral grade). |
| **Medications** | Aspirin 75 mg/day (only). No anticoagulation, no DOAC. |
| **Task** | What is the most appropriate reperfusion strategy? Provide rationale for: (1) IV alteplase decision, (2) mechanical thrombectomy device and technique selection, (3) blood pressure management, and (4) post-procedure antiplatelet management. |

| **Vignette 3 of 8 \| V-NEURO-03** |  |
| --- | --- |
| **Patient** | 72-year-old male. Recent TIA (amaurosis fugax right eye, fully resolved within 20 minutes, 10 days ago). Hypertension, dyslipidaemia, non-diabetic, ex-smoker. BMI 26.4 kg/m². eGFR 74 mL/min/1.73m². Currently on aspirin 100 mg/day and clopidogrel 75 mg/day (commenced after TIA). |
| **Presentation** | Referred urgently for vascular assessment following TIA. Clinically: right carotid bruit on auscultation. NIHSS 0. |
| **Investigations** | Carotid duplex: right internal carotid artery (ICA) stenosis 82% (NASCET criteria). Left ICA stenosis 35%. MRI brain: no acute infarction, small old lacunar infarcts (not carotid territory). Cardiac monitoring: no AF detected on 7-day Holter. Echocardiography: no structural source of embolism. LDL 3.2 mmol/L. |
| **Medications** | Aspirin 100 mg/day, clopidogrel 75 mg/day, amlodipine 10 mg/day, atorvastatin 40 mg/day. |
| **Task** | What is the most appropriate management strategy for this symptomatic high-grade carotid stenosis? Provide rationale for: (1) indication and timing for carotid intervention, (2) CEA versus CAS decision, (3) perioperative medical management, and (4) long-term secondary prevention. |

| **Vignette 4 of 8 \| V-NEURO-04** |  |
| --- | --- |
| **Patient** | 34-year-old female. No prior neurological history. No cardiovascular risk factors. Non-smoker, non-diabetic, normotensive. BMI 22.4 kg/m². On combined oral contraceptive pill (ethinylestradiol/desogestrel). No family history of aneurysm. |
| **Presentation** | Sudden catastrophic headache ('thunderclap headache'), worst of life, onset 4 hours ago. GCS on arrival 13/15 (confused). No focal neurological deficit. Meningism on examination. BP 148/92 mmHg. |
| **Investigations** | CT head: diffuse subarachnoid haemorrhage (SAH), Fisher Grade 3 (thick clot in basal cisterns), modified Fisher Grade 3. CT angiography: anterior communicating artery (AComA) aneurysm, 9 mm dome, irregular morphology (daughter sac), wide neck 4.5 mm, dome-to-neck ratio 2.0. No other aneurysm. |
| **Medications** | Combined OCP. No anticoagulation. |
| **Task** | What is the emergency management and treatment strategy for this ruptured AComA aneurysm? Provide rationale for: (1) immediate resuscitation and medical management, (2) endovascular versus surgical treatment decision, (3) device selection for endovascular treatment, and (4) management of the OCP. |

| **Vignette 5 of 8 \| V-NEURO-05 ★ SENTINEL VIGNETTE — Pre-designated internal validity anchor** |  |
| --- | --- |
| **Patient** | 55-year-old male. No prior neurological history. Hypertension (amlodipine 5 mg/day, ramipril 10 mg/day), non-diabetic, ex-smoker. BMI 27.2 kg/m². eGFR 81 mL/min/1.73m². Admitted 3 weeks ago with right basal ganglia hypertensive intracerebral haemorrhage (ICH), managed conservatively. Good neurological recovery (mRS 1 on discharge). Now returning for outpatient follow-up. |
| **Presentation** | Asymptomatic at outpatient follow-up. MRI brain: ICH resolved, hemosiderin staining at the site. Incidental finding on MRA: left middle cerebral artery (MCA) fusiform aneurysm, 12 mm, irregular morphology, arising at the M2 bifurcation. |
| **Investigations** | DSA: left M2 MCA fusiform aneurysm, 12 mm diameter, involving the bifurcation. Morphology: fusiform, irregular, involving a 2 cm segment of M2, with two M3 branches arising from the aneurysm dome. No daughter sacs. PHASES score: 8 (estimated 5-year rupture risk approximately 15%). |
| **Medications** | Amlodipine 5 mg/day, ramipril 10 mg/day. No antiplatelet therapy (held post-ICH). |
| **Task** | What is the management strategy for this incidentally discovered MCA fusiform aneurysm in a patient with recent ICH? Provide rationale for: (1) risk stratification, (2) treatment decision, (3) device selection for endovascular or surgical treatment, and (4) timing considerations given recent ICH. |

| **Vignette 6 of 8 \| V-NEURO-06** |  |
| --- | --- |
| **Patient** | 61-year-old male. Known COPD (FEV1 60%), hypertension, dyslipidaemia. Non-diabetic. eGFR 72 mL/min/1.73m². Previous right CEA for symptomatic carotid stenosis 4 years ago. Currently on aspirin 100 mg/day, atorvastatin 40 mg/day. |
| **Presentation** | Asymptomatic. Carotid duplex surveillance at 3 years post-CEA: right ICA restenosis detected. No new neurological symptoms. NIHSS 0. |
| **Investigations** | Carotid duplex and CTA: right ICA restenosis 72% (NASCET criteria) at the prior endarterectomy site. Left ICA 40% stenosis. No new cerebral infarction on MRI. No AF on recent ECG. |
| **Medications** | Aspirin 100 mg/day, atorvastatin 40 mg/day, amlodipine 10 mg/day, tiotropium inhaler, salmeterol/fluticasone inhaler. |
| **Task** | What is the management strategy for this post-CEA restenosis? Provide rationale for: (1) whether intervention is indicated for asymptomatic restenosis, (2) carotid stenting versus redo-CEA, (3) embolic protection strategy, and (4) antiplatelet management. |

| **Vignette 7 of 8 \| V-NEURO-07** |  |
| --- | --- |
| **Patient** | 49-year-old male. No cardiovascular risk factors. Non-smoker, non-diabetic, normotensive. BMI 23.4 kg/m². No medications. Referred from neurology after incidental MRI finding. |
| **Presentation** | Incidental finding of left frontal dural arteriovenous fistula (dAVF) on contrast MRI performed for evaluation of headache. No neurological deficit. No history of haemorrhage. No tinnitus or other symptoms of dAVF. |
| **Investigations** | DSA: left frontal dAVF, Borden Type I (meningeal arterial supply, draining to meningeal veins, no cortical venous reflux). Feeding arteries: middle meningeal artery branches bilaterally. No cortical venous drainage, no venous varix. No other vascular malformation. |
| **Medications** | No medications. |
| **Task** | What is the appropriate management strategy for this Borden Type I dAVF? Provide rationale for: (1) risk stratification based on Borden classification, (2) treatment versus observation decision, (3) if treatment: modality selection, and (4) follow-up protocol. |

| **Vignette 8 of 8 \| V-NEURO-08** |  |
| --- | --- |
| **Patient** | 71-year-old female. Hypertension (ramipril 10 mg/day, indapamide 2.5 mg/day), dyslipidaemia (rosuvastatin 20 mg/day), non-diabetic, non-smoker. BMI 25.8 kg/m². eGFR 64 mL/min/1.73m². On aspirin 100 mg/day. History: vertebrobasilar TIA 6 months ago — transient diplopia and ataxia, fully resolved. |
| **Presentation** | Outpatient follow-up after vertebrobasilar TIA. Asymptomatic at present. MRA brain and neck performed after TIA: high-grade right vertebral artery origin stenosis (85%, V1 segment) on initial MRA — confirmed on CTA. Left vertebral artery dominant and patent throughout. No posterior circulation infarction on MRI at time of TIA. |
| **Investigations** | DSA (selective vertebral arteriography): right vertebral artery origin (V1) high-grade stenosis 87% (NASCET-equivalent). Left VA dominant, fully patent. No significant basilar artery disease. Posterior circulation perfusion: maintained predominantly via left VA. |
| **Medications** | Aspirin 100 mg/day, clopidogrel 75 mg/day (added after TIA), ramipril 10 mg/day, indapamide 2.5 mg/day, rosuvastatin 20 mg/day. |
| **Task** | What is the appropriate management strategy for this symptomatic vertebral artery origin stenosis? Provide rationale for: (1) medical vs. endovascular treatment decision, (2) if endovascular — device selection, (3) risks specific to vertebral artery origin stenting, and (4) long-term secondary prevention. |

*Supplementary File S2 | Clinical Vignettes (40) | When AI Grades AI | Dinc R, Ardic N | INVAMED / Med-International UK | May 2026*
