# Supplementary material for: Systematic Scoring Differences in Large Language Model–Based Clinical Benchmarking Reveal Evaluator-Dependent Divergence: Controlled Evaluation Study
Source: JMIR AI. 2026 Oct 26;5:e103962. doi: 10.2196/103962 (PMC13705216; doi:10.2196/103962)
Supplement: Supplementary file 4 [file ai-v5-e103962-s003.docx]

**SUPPLEMENTARY FILE S1**

**C6 Scoring Anchors, Sentinel Vignette, and Mis-Assignment Logs**

When AI Grades AI: Systematic Scoring Differences in LLM-Based Clinical Benchmarking Reveal Evaluator-Dependent Divergence

*Dinc R, Ardic N | INVAMED / Med-International UK | May 2026*

This file contains four tables:

- Table S1.1 — C6 PARM Reflection Scoring Anchors: C6=2, C6=1, C6=0 with operational definitions and dataset examples
- Table S1.2 — Scoring Decision Guide
- Table S1.3 — V-NEURO-05 Sentinel Vignette: Full text, correct management, and cross-model scoring
- Table S1.4 — M4 Llama 3.3 70B Vignette Mis-Assignment Log (11 vignettes)
- Table S1.5 — M3 Gemini 1.5 Pro Vignette Mis-Assignment Log (15 vignettes)

*The prespecified C6 anchors in Table S1.1 were finalised before any model was evaluated and applied identically by both AI raters. These anchors are referenced in Section 2.3 of the main manuscript.*

**Table S1.1.** C6 PARM Reflection Scoring Anchors — Operational Definitions

| **Score** | **Operational definition** | **Example from dataset** |
| --- | --- | --- |
| **C6 = 2 Full Reflection (Complete)** | ALL THREE elements must be present as a structured deliberation: (a) Explicit acknowledgement of clinical uncertainty specific to this patient — not generic hedging (b) Formal articulation of at least one named alternative management strategy, with clinical rationale, for when the primary strategy is contraindicated or fails (c) Explicit acknowledgement of evidence limitations — stated separately from the recommendation itself | *V-NEURO-05, M2 (Claude Sonnet 4.6): (a) 'The DAPT requirement of PED creates an irresolvable tension in a patient 3 weeks post-ICH.' (b) Two alternatives: EC-IC bypass + trapping; deferred PED at 3–6 months with antiplatelet protocol. (c) 'Evidence for flow diversion timing post-ICH is limited to case series; no RCT data available.' → Only response achieving C6=2 in the full dataset.* |
| **C6 = 1 Partial Reflection** | At least ONE of elements (a), (b), or (c) present — but NOT all three: • Uncertainty acknowledged vaguely rather than patient-specifically • Alternative mentioned but not formally argued • Evidence limitation noted within the recommendation, not as a distinct deliberation | *M1 GPT-4o on V-NEURO-05 under Rater 2 (GPT-4o): DAPT conflict identified but no formal alternative strategy presented → C6=1 under Rater 2. Under Rater 1 (Claude Sonnet 4.6), full marks awarded (C6=2) per original scoring report. M2 Claude on V-PAD-01: Notes paclitaxel safety signal (evidence limitation) but embedded in recommendation.* |
| **C6 = 0 No Reflection (Absent)** | NONE of elements (a), (b), or (c) present in any form. Generic closing phrases such as 'further research is needed' or 'consult your physician' in isolation do NOT qualify for C6=1. Requirements must be traceable to specific statements in the response. | *All 40 M4 Llama 3.3 70B responses under Rater 1: complete absence of Reflection across all domains. M4 on V-NEURO-05 under both raters: flow diverter recommended without identifying DAPT contraindication or alternatives.* |

**Table S1.2.** Scoring Decision Guide for C6

| **Question to ask when scoring C6** | **If YES →** | **If NO →** |
| --- | --- | --- |
| Does the response explicitly acknowledge uncertainty specific to this patient/case? | Element (a) present | Element (a) absent — check (b) and (c) |
| Does the response formally name ≥1 alternative strategy with clinical rationale for when primary fails or is contraindicated? | Element (b) present | Element (b) absent |
| Does the response acknowledge evidence limitations separately from the recommendation? | Element (c) present | Element (c) absent |
| Are ALL THREE elements (a), (b), AND (c) present as a structured deliberation? | **C6 = 2** | → Next question |
| Is at least ONE of (a), (b), or (c) present in any form? | **C6 = 1** | **C6 = 0** |

**Table S1.3.** V-NEURO-05 Sentinel Vignette — Full Text, Correct Management, and Cross-Model Scoring

*V-NEURO-05 was designated a priori as the internal validity anchor before any model was evaluated. It contains an objectively verifiable contraindication whose correct recognition does not depend on clinical opinion.*

**Vignette text (submitted identically to all four models):**

A 58-year-old male presents with an incidentally discovered fusiform aneurysm of the right middle cerebral artery (MCA) M1 segment, measuring 12mm in maximum diameter. The aneurysm has a fusiform morphology with no distinct neck. MRI was performed as part of a work-up for a transient ischaemic attack (TIA), at which point a 7mm left basal ganglia intracerebral haemorrhage (ICH) was also identified. The ICH occurred 3 weeks ago; the patient was managed conservatively and is currently neurologically intact. Current medications: amlodipine 5mg, atorvastatin 40mg. No antiplatelet therapy. NIHSS 0. PHASES score: 8. What is your recommended management strategy for the right MCA fusiform aneurysm, and what device or treatment approach do you recommend?

| **Element** | **Correct clinical answer** |
| --- | --- |
| **Primary device** | Flow diverter (Pipeline Embolization Device or FRED) — standard for fusiform MCA aneurysms without clippable neck. |
| **Critical contraindication** | DAPT CONTRAINDICATED. Flow diverters require mandatory DAPT (aspirin + clopidogrel, 3–6 months minimum). DAPT is contraindicated in a patient with ICH 3 weeks prior. This contraindication makes immediate flow diverter placement clinically unsafe — this is the objectively verifiable item that does not depend on clinical opinion. |
| **C3=2 requires** | Identify DAPT requirement of flow diverter AND identify recent ICH as contraindication to DAPT. C3=0 if neither is identified. |
| **C6=2 requires** | (a) Acknowledge DAPT-in-post-ICH creates irresolvable tension; (b) Present ≥1 formal alternative (EC-IC bypass + trapping; or staged PED at 3–6 months with antiplatelet protocol); (c) Acknowledge limited evidence for antiplatelet re-introduction timing post-ICH. |

**Cross-model C3 and C6 scores on V-NEURO-05:**

| **Model** | **C3 Rater 1** | **C3 Rater 2** | **C6 Rater 1** | **C6 Rater 2** | **Clinical note** |
| --- | --- | --- | --- | --- | --- |
| **M1 GPT-4o** | 2 | 1 | 2 | 1 | Full marks — antiplatelet/post-ICH conflict correctly identified as central issue, with formal alternative strategy and evidence limitation acknowledged. Original scoring rationale: "None — full marks." |
| **M2 Claude Sonnet 4.6** | **2** | **2** | **2** | **2** | Only response achieving C6=2 in full dataset. Both raters agreed. |
| **M3 Gemini 1.5 Pro** | 1 | 1 | 1 | 1 | DAPT timing gap identified; bridge management absent |
| **M4 Llama 3.3 70B** | **0** | **0** | **0** | **0** | C3=0: DAPT contraindication completely absent. Only C3=0 on an objectively verifiable item in entire dataset. Both raters agreed independently. |

*C3=0 for M4 agreed by both raters independently — the only criterion score of 0 on an objectively verifiable item in the full 4-model × 40-vignette × 2-rater dataset.*

**Table S1.4.** M4 Llama 3.3 70B Vignette Mis-Assignment Log (11 of 40 vignettes, 27.5%)

*Llama 3.3 70B mis-assigned responses to 11 of 40 vignettes (27.5%), consistently defaulting to SFA PAD content. Rater 1 (reasoning-quality approach) awarded partial credit; Rater 2 (context-fidelity approach) awarded near-zero. Domains: AAA ×4, CVI ×4, ISR ×1, NEURO ×1.*

| **Vignette** | **Expected content** | **Actual content returned** | **Impact on scoring** |
| --- | --- | --- | --- |
| **V-AAA-04** | 66-year-old female, 42mm AAA — surveillance only | *SFA PAD claudication content (DCB for femoropopliteal lesion)* | C1/C2/C4=1; C5=2; C6=0 |
| **V-AAA-05** | 74-year-old male, 58mm AAA, mild COPD, prior PCI — EVAR | *SFA PAD claudication content* | C1/C2/C4=1; C5=2; C6=0 |
| **V-AAA-06** | 66-year-old female, 42mm AAA — surveillance only | *Type B aortic dissection content* | C1/C2/C4=1; C5=2; C6=0 |
| **V-AAA-08** | 74-year-old male, juxtarenal AAA — FEVAR | *SFA PAD claudication content* | C1/C2/C4=1; C5=2; C6=0 |
| **V-CVI-02** | 63-year-old male, TASC IIC right CIA 7cm occlusion | *Venous reflux (GSV/SSV) content* | C1/C2/C4=1; C5=2; C6=0 |
| **V-CVI-05** | 45-year-old female, 3.1cm AAA, family history | *AAGSV thrombophlebitis content* | C1/C2/C4=1; C5=2; C6=0 |
| **V-CVI-06** | 72-year-old female, CLTI, ESRD, heel ulcer | *CEAP C1 telangiectasias content* | C1/C2/C4=1; C5=2; C6=0 |
| **V-CVI-07** | 71-year-old male, 75% ICA stenosis, hostile neck — CAS + EPD | *Bilateral PTS / May-Thurner venous content* | C1/C2/C4=1; C5=2; C6=0 |
| **V-CVI-08** | 29-year-old female, CEAP C1 — microsclerotherapy | *Pelvic venous congestion content* | C1/C2/C4=1; C5=2; C6=0 |
| **V-ISR-07** | Frail demented patient, CFS 5, RCA ISR — conservative | *SFA PAD claudication content* | C1/C2/C4=1; C5=2; C6=0 |
| **V-NEURO-07** | Borden Type I dAVF — observation only | *SFA PAD claudication content* | C1/C2/C4=1; C5=2; C6=0 |

*Rater 1 (reasoning-quality): C1=C2=C4=1, C5=2, C3=1, C6=0. Rater 2 (context-fidelity): C1=C2=C5=0, C3=1, C4=1, C6=0. Total: 11/40 (27.5%). Domains: AAA ×4, CVI ×4, ISR ×1, NEURO ×1.*

**Table S1.5.** M3 Gemini 1.5 Pro Vignette Mis-Assignment Log (15 of 40 vignettes, 37.5%)

*Gemini 1.5 Pro mis-assigned responses to 15 of 40 vignettes (37.5%) under Rater 2's context-fidelity approach. Rater 1 awarded partial credit (reasoning-quality approach). The 15 mis-assigned vignettes are the primary driver of the 158-point inter-rater gap (Rater 1: 431/480; Rater 2: 273/480). Domains: AAA ×3, CVI ×6, ISR ×5, NEURO ×1.*

| **Vignette** | **Expected content** | **Content returned by Gemini** | **Rater 2 scores (context-fidelity)** |
| --- | --- | --- | --- |
| **V-AAA-04** | 71-year-old male, asymptomatic, 3 years post-EVAR surveillance CT with sac growth — Type II endoleak management | *Cross-contaminated AAA content; correct sac-growth/endoleak management absent* | C1=0, C2=0, C3=1, C4=1, C5=0, C6=0 \| Total: 2/12 |
| **V-AAA-05** | 66-year-old female, incidentally detected 42mm AAA on renal US — surveillance threshold and conservative management | *Wrong-domain content; AAA surveillance management context absent* | C1=0, C2=0, C3=0, C4=1, C5=0, C6=0 \| Total: 1/12 |
| **V-AAA-06** | 67-year-old male, known 53mm AAA, acute chest/back pain, BP differential, pulse deficit — acute aortic syndrome (Type B dissection) | *Generic AAA content returned; acute aortic syndrome / dissection context absent* | C1=0, C2=0, C3=1, C4=1, C5=0, C6=0 \| Total: 2/12 |
| **V-CVI-02** | 44-year-old male, previous right leg DVT 5 years ago, now off anticoagulation — chronic venous insufficiency / GSV reflux assessment | *Venous reflux content returned but for wrong patient profile; cross-contamination from adjacent vignette* | C1=0, C2=0, C3=0, C4=1, C5=0, C6=0 \| Total: 1/12 |
| **V-CVI-03** | 38-year-old female, 28 weeks pregnant, varicose veins — conservative venous management during pregnancy | *Wrong-domain content; pregnancy varicosity conservative management absent* | C1=0, C2=0, C3=0, C4=1, C5=0, C6=0 \| Total: 1/12 |
| **V-CVI-05** | 51-year-old male, ex-rugby player, sedentary, BMI 29, no prior DVT — CVI / venous insufficiency assessment | *Wrong-domain content; CVI scenario for this patient absent* | C1=0, C2=0, C3=0, C4=1, C5=0, C6=0 \| Total: 1/12 |
| **V-CVI-06** | 29-year-old female, competitive swimmer, BMI 22, no comorbidities — CVI / venous insufficiency assessment | *Wrong-domain content; venous assessment for this patient absent* | C1=0, C2=0, C3=0, C4=1, C5=0, C6=0 \| Total: 1/12 |
| **V-CVI-07** | 58-year-old male, morbidly obese (BMI 44), insulin-dependent diabetes, prior DVT, bilateral hip replacements — complex CVI management | *Wrong-domain content; complex CVI/obesity/DVT scenario absent* | C1=0, C2=0, C3=0, C4=1, C5=0, C6=0 \| Total: 1/12 |
| **V-CVI-08** | 34-year-old male, tall/slim (BMI 20, 193cm), incidental finding during varicocoele assessment — nutcracker syndrome / left renal vein compression | *Wrong-domain content; nutcracker/varicocoele venous context absent* | C1=0, C2=0, C3=0, C4=1, C5=0, C6=0 \| Total: 1/12 |
| **V-ISR-02** | 74-year-old female, previous RCA STEMI with primary PCI (EES), LVEF 48%, inferior hypokinesia — RCA in-stent restenosis management | *Wrong-domain content; RCA ISR clinical scenario absent* | C1=0, C2=0, C3=0, C4=1, C5=0, C6=0 \| Total: 1/12 |
| **V-ISR-03** | 52-year-old male, previous LCx OM2 PCI (sirolimus-eluting stent), HbA1c 7.1%, LVEF 62% — LCx OM2 ISR management | *Wrong-domain content; LCx OM2 ISR context absent* | C1=0, C2=0, C3=0, C4=1, C5=0, C6=0 \| Total: 1/12 |
| **V-ISR-05** | 48-year-old male, previous proximal LAD PCI (EES), AERD (aspirin causes bronchospasm), LVEF 62% — LAD ISR with AERD management | *Wrong-domain content; LAD ISR + AERD scenario absent* | C1=0, C2=0, C3=0, C4=1, C5=0, C6=0 \| Total: 1/12 |
| **V-ISR-06** | 69-year-old female, previous LM PCI (provisional bifurcation), diabetes HbA1c 8.4%, LVEF 55% — left main ISR management | *Left main bifurcation ISR context partially missing from response* | C1=0, C2=0, C3=1, C4=1, C5=0, C6=0 \| Total: 2/12 |
| **V-ISR-08** | 42-year-old male, marathon runner, no cardiac history, family history MI at 54, LDL 4.8 mmol/L — STEMI post-ROSC primary PCI | *Wrong-domain content; STEMI post-ROSC clinical scenario absent* | C1=0, C2=0, C3=0, C4=1, C5=0, C6=0 \| Total: 1/12 |
| **V-NEURO-08** | 71-year-old female, vertebrobasilar TIA 6 months ago, on aspirin, hypertension, dyslipidaemia — vertebral artery origin stenosis management | *Wrong-domain content; vertebral artery stenosis context absent* | C1=0, C2=0, C3=0, C4=1, C5=0, C6=0 \| Total: 1/12 |
| SUMMARY: 15/40 (37.5%) mis-assigned. Rater 2 scores C1=C2=C5=0 for all mis-assigned vignettes (context-fidelity approach). Rater 1 awards C1=1, C2=1–2, C5=2 (reasoning-quality approach). Mean Rater 2 score for mis-assigned vignettes: 1.3/12 vs Rater 1 mean: ~10/12. This difference (≈8.7 points × 15 vignettes = 130 points) plus criterion-threshold differences on correctly assigned vignettes accounts for the 158-point gap. | | | |

*Mis-assignment defined as C1=0 AND C2=0 under Rater 2 (context-fidelity). Identical C6 anchors were provided to both raters (Table S1.1); the divergence reflects rater-specific criterion application, not differing anchor definitions.*

*Supplementary File S1 | When AI Grades AI | Dinc R, Ardic N | INVAMED / Med-International UK | 2026*
