# Supplementary material for: Systematic Scoring Differences in Large Language Model–Based Clinical Benchmarking Reveal Evaluator-Dependent Divergence: Controlled Evaluation Study
Source: JMIR AI. 2026 Oct 26;5:e103962. doi: 10.2196/103962 (PMC13705216; doi:10.2196/103962)
Supplement: Supplementary file 5 [file ai-v5-e103962-s004.docx]

**SUPPLEMENTARY FILE S3A**

**Scoring Report — M1: GPT-4o**

When AI Grades AI: Systematic Scoring Differences in LLM-Based Clinical Benchmarking Reveal Evaluator-Dependent Divergence

*Dinc R, Ardic N | INVAMED / Med-International UK | May 2026*

| **Model evaluated** | **M1 — GPT-4o (OpenAI; accessed via chatgpt.com, May 2026)** |
| --- | --- |
| **Rater 1** | Claude Sonnet 4.6 (also evaluated as M2) \| Score: 455/480 (95%) |
| **Rater 2** | **GPT-4o (also evaluated as M1) \| Score: 409/480 (85%)** |
| **Rubric** | C1–C6, 0–2 per criterion; maximum 12 per vignette; maximum 480 total |
| **Inter-rater delta (R2−R1)** | **-46 points** |

# 1. Criterion-Level Scores

| **Criterion** | **Rater 1 (Claude) Score** | **Rater 1 %** | **Rater 2 (GPT-4o) Score** | **Rater 2 %** |
| --- | --- | --- | --- | --- |
| C1 — Guideline concordance | **80/80** | 100% | **72/80** | 90% |
| C2 — Anatomical appropriateness | **79/80** | 99% | **71/80** | 89% |
| C3 — Contraindication recognition | **77/80** | 96% | **66/80** | 83% |
| C4 — Reasoning quality | **80/80** | 100% | **77/80** | 96% |
| C5 — Hallucination/error rate | **79/80** | 99% | **63/80** | 79% |
| **C6 — PARM Reflection** | **60/80** | 75% | **60/80** | 75% |
| **TOTAL** | **455/480** | 95% | **409/480** | 85% |

*Amber shading ≥75%; green ≥90%. † C6 (PARM Reflection) highlighted: the criterion showing the most notable evaluator-dependent variation across raters.*

# 2. Domain-Level Scores

| **Domain** | **R1 Score** | **R1 %** | **R2 Score** | **R2 %** | **R1+R2 Total** | **%** | **Dominant pattern (R2)** |
| --- | --- | --- | --- | --- | --- | --- | --- |
| **PAD** | *—* | *—* | 89/96 | 93% | *—* | *—* | *Excellent device selection and pharmacotherapy integration* |
| **AAA** | *—* | *—* | 83/96 | 86% | *—* | *—* | *Strong EVAR/FEVAR/TEVAR judgement with nuanced surveillance logic* |
| **CVI** | *—* | *—* | 79/96 | 82% | *—* | *—* | *Consistent reflux-targeting and compression integration* |
| **ISR** | *—* | *—* | 77/96 | 80% | *—* | *—* | *Strong intracoronary reasoning and imaging-guided management* |
| **NEURO** | *—* | *—* | 81/96 | 84% | *—* | *—* | *Excellent acute neurovascular sequencing and thrombectomy logic* |
| **TOTAL** | **455/480** | 95% | **409/480** | 85% | — | — |  |

*R1 domain-level breakdown not retained at individual criterion level for Rater 1. R2 = Rater 2 (GPT-4o) verified domain scores from original scoring records.*

# 3. Vignette Score Matrix — Rater 2 (GPT-4o)

*Per-vignette scores assigned by Rater 2 (GPT-4o) across all 40 vignettes and 6 criteria. The Rater 2 vignette-level criterion scores shown below were reconstructed from the verified aggregate criterion totals because the original per-vignette Rater 2 criterion breakdown for M1 GPT-4o was not retained. The reconstruction procedure and its limitations are documented in Supplementary File S4.*

| **Vignette** | **C1** | **C2** | **C3** | **C4** | **C5** | **C6** | **Total / Key gap** |
| --- | --- | --- | --- | --- | --- | --- | --- |
| V-PAD-01 | 2 | 2 | 2 | 2 | 2 | 2 | 12/12 (100%) |
| V-PAD-02 | 2 | 2 | 2 | 2 | 2 | 2 | 12/12 (100%) |
| V-PAD-03 | 2 | 2 | 2 | 2 | 2 | 2 | 12/12 (100%) |
| V-PAD-04 | 2 | 2 | 2 | 2 | 2 | 2 | 12/12 (100%) |
| V-PAD-05 | 2 | 2 | 2 | 2 | 2 | 1 | 11/12 (92%) |
| V-PAD-06 | 2 | 2 | 2 | 2 | 2 | 2 | 12/12 (100%) |
| V-PAD-07 | 2 | 2 | 2 | 2 | 2 | 2 | 12/12 (100%) |
| V-PAD-08 | 2 | 2 | 2 | 2 | 2 | 2 | 12/12 (100%) |
| V-AAA-01 | 2 | 2 | 2 | 2 | 2 | 2 | 12/12 (100%) |
| V-AAA-02 | 2 | 2 | 2 | 2 | 2 | 1 | 11/12 (92%) |
| V-AAA-03 | 2 | 2 | 2 | 2 | 2 | 2 | 12/12 (100%) |
| V-AAA-04 | 2 | 2 | 2 | 2 | 2 | 2 | 12/12 (100%) |
| V-AAA-05 | 2 | 2 | 2 | 2 | 2 | 2 | 12/12 (100%) |
| V-AAA-06 | 2 | 2 | 2 | 2 | 2 | 1 | 11/12 (92%) |
| V-AAA-07 | 2 | 2 | 1 | 2 | 2 | 2 | 11/12 (92%) |
| V-AAA-08 | 2 | 2 | 1 | 2 | 2 | 1 | 10/12 (83%) |
| V-CVI-01 | 2 | 2 | 2 | 2 | 2 | 2 | 12/12 (100%) |
| V-CVI-02 | 2 | 2 | 2 | 2 | 2 | 2 | 12/12 (100%) |
| V-CVI-03 | 2 | 2 | 2 | 2 | 2 | 2 | 12/12 (100%) |
| V-CVI-04 | 2 | 2 | 2 | 2 | 2 | 2 | 12/12 (100%) |
| V-CVI-05 | 2 | 2 | 2 | 2 | 2 | 2 | 12/12 (100%) |
| V-CVI-06 | 2 | 1 | 2 | 2 | 2 | 1 | 10/12 (83%) |
| V-CVI-07 | 2 | 2 | 2 | 2 | 1 | 1 | 10/12 (83%) |
| V-CVI-08 | 1 | 1 | 1 | 2 | 1 | 1 | 7/12 (58%) |
| V-ISR-01 | 2 | 2 | 2 | 2 | 2 | 2 | 12/12 (100%) |
| V-ISR-02 | 2 | 2 | 1 | 2 | 2 | 1 | 10/12 (83%) |
| V-ISR-03 | 2 | 2 | 2 | 2 | 2 | 2 | 12/12 (100%) |
| V-ISR-04 | 2 | 2 | 2 | 2 | 2 | 2 | 12/12 (100%) |
| V-ISR-05 | 2 | 2 | 1 | 2 | 1 | 1 | 9/12 (75%) |
| V-ISR-06 | 2 | 2 | 2 | 2 | 2 | 1 | 11/12 (92%) |
| V-ISR-07 | 2 | 2 | 2 | 2 | 2 | 2 | 12/12 (100%) |
| V-ISR-08 | 2 | 2 | 1 | 2 | 1 | 1 | 9/12 (75%) |
| V-NEURO-01 | 2 | 2 | 2 | 2 | 2 | 2 | 12/12 (100%) |
| V-NEURO-02 | 2 | 2 | 2 | 2 | 2 | 2 | 12/12 (100%) |
| V-NEURO-03 | 2 | 2 | 2 | 2 | 2 | 2 | 12/12 (100%) |
| V-NEURO-04 | 2 | 2 | 2 | 2 | 2 | 2 | 12/12 (100%) |
| **V-NEURO-05 ★** | 1 | 1 | **1** | 1 | 1 | 1 | 6/12 (50%) |
| V-NEURO-06 | 2 | 2 | 1 | 2 | 2 | 1 | 10/12 (83%) |
| V-NEURO-07 | 2 | 2 | 2 | 2 | 2 | 2 | 12/12 (100%) |
| V-NEURO-08 | 2 | 2 | 1 | 2 | 1 | 1 | 9/12 (75%) |
| **PAD Subtotal (Rater 2): 95/96 (99%) \| C1:16 C2:16 C3:16 C4:16 C5:16 C6:15** | | | | | | | |
| **AAA Subtotal (Rater 2): 91/96 (95%) \| C1:16 C2:16 C3:14 C4:16 C5:16 C6:13** | | | | | | | |
| **CVI Subtotal (Rater 2): 87/96 (91%) \| C1:15 C2:14 C3:15 C4:16 C5:14 C6:13** | | | | | | | |
| **ISR Subtotal (Rater 2): 87/96 (91%) \| C1:16 C2:16 C3:13 C4:16 C5:14 C6:12** | | | | | | | |
| **NEURO Subtotal (Rater 2): 84/96 (88%) \| C1:15 C2:15 C3:12 C4:15 C5:14 C6:13** | | | | | | | |
| **GRAND TOTAL (Rater 2): 409/480 (85%)** | | | | | | | |

*★ V-NEURO-05: pre-designated sentinel vignette. C6=2 was achieved only by M2 on V-NEURO-05 under both raters. Rater 2 = GPT-4o (context-fidelity scoring approach).*

*Supplementary File S3A | M1 GPT-4o Scoring Report | When AI Grades AI | Dinc R, Ardic N | 2026*
