# Supplementary material for: Systematic Scoring Differences in Large Language Model–Based Clinical Benchmarking Reveal Evaluator-Dependent Divergence: Controlled Evaluation Study
Source: JMIR AI. 2026 Oct 26;5:e103962. doi: 10.2196/103962 (PMC13705216; doi:10.2196/103962)
Supplement: Supplementary file 6 [file ai-v5-e103962-s005.docx]

**SUPPLEMENTARY FILE S3B**

**Scoring Report — M2: Claude Sonnet 4.6**

When AI Grades AI: Systematic Scoring Differences in LLM-Based Clinical Benchmarking Reveal Evaluator-Dependent Divergence

*Dinc R, Ardic N | INVAMED / Med-International UK | May 2026*

| **Model evaluated** | **M2 — Claude Sonnet 4.6 (Anthropic; accessed via claude.ai, May 2026)** |
| --- | --- |
| **Rater 1** | Claude Sonnet 4.6 (also evaluated as M2) \| Score: 441/480 (92%) |
| **Rater 2** | **GPT-4o (also evaluated as M1) \| Score: 446/480 (93%)** |
| **Rubric** | C1–C6, 0–2 per criterion; maximum 12 per vignette; maximum 480 total |
| **Inter-rater delta (R2−R1)** | **+5 points** |

# 1. Criterion-Level Scores

| **Criterion** | **Rater 1 (Claude) Score** | **Rater 1 %** | **Rater 2 (GPT-4o) Score** | **Rater 2 %** |
| --- | --- | --- | --- | --- |
| C1 — Guideline concordance | **80/80** | 100% | **75/80** | 94% |
| C2 — Anatomical appropriateness | **80/80** | 100% | **77/80** | 96% |
| C3 — Contraindication recognition | **80/80** | 100% | **77/80** | 96% |
| C4 — Reasoning quality | **80/80** | 100% | **80/80** | 100% |
| C5 — Hallucination/error rate | **80/80** | 100% | **62/80** | 78% |
| **C6 — PARM Reflection** | **41/80** | 51% | **75/80** | 94% |
| **TOTAL** | **441/480** | 92% | **446/480** | 93% |

*Amber shading ≥75%; green ≥90%. † C6 (PARM Reflection) highlighted because it showed substantial evaluator-dependent variation between raters.*

# 2. Domain-Level Scores

| **Domain** | **R1 Score** | **R1 %** | **R2 Score** | **R2 %** | **R1+R2 Total** | **%** | **Dominant pattern (R2)** |
| --- | --- | --- | --- | --- | --- | --- | --- |
| **PAD** | *—* | *—* | 86/96 | 90% | *—* | *—* | *Excellent procedural detail; some overstatement and overly aggressive device cer...* |
| **AAA** | *—* | *—* | 88/96 | 92% | *—* | *—* | *Strong EVAR/FEVAR logic; V-AAA-06 overcalled as urgent TEVAR* |
| **CVI** | *—* | *—* | 92/96 | 96% | *—* | *—* | *Very strong reflux-targeting and contraindication recognition* |
| **ISR** | *—* | *—* | 87/96 | 91% | *—* | *—* | *Strong mechanism-first ISR reasoning; some overconfidence in high-risk PCI* |
| **NEURO** | *—* | *—* | 93/96 | 97% | *—* | *—* | *Excellent neurovascular decision-making and conservative-treatment recognition* |
| **TOTAL** | **441/480** | 92% | **446/480** | 93% | — | — |  |

*Rater 1 (Claude Sonnet 4.6) per-vignette criterion-level scores are original retained records from the Phase 2B scoring session, documented in the original scoring reports (reproduced in Supplementary Files S3a–S3d for audit). R2 = Rater 2 (GPT-4o) verified domain scores from original scoring records.*

# 3. Vignette Score Matrix — Rater 2 (GPT-4o)

*Per-vignette scores assigned by Rater 2 (GPT-4o) across all 40 vignettes and 6 criteria. These are the externally verified Rater 2 scores from the original scoring records.*

| **Vignette** | **C1** | **C2** | **C3** | **C4** | **C5** | **C6** | **Total / Key gap** |
| --- | --- | --- | --- | --- | --- | --- | --- |
| V-PAD-01 | 2 | 2 | 2 | 2 | 1 | 2 | 11/12 (92%) |
| V-PAD-02 | 2 | 2 | 2 | 2 | 1 | 2 | 11/12 (92%) |
| V-PAD-03 | 1 | 2 | 2 | 2 | 1 | 2 | 10/12 (83%) |
| V-PAD-04 | 2 | 2 | 2 | 2 | 1 | 2 | 11/12 (92%) |
| V-PAD-05 | 2 | 2 | 2 | 2 | 1 | 2 | 11/12 (92%) |
| V-PAD-06 | 2 | 2 | 2 | 2 | 1 | 2 | 11/12 (92%) |
| V-PAD-07 | 1 | 2 | 2 | 2 | 2 | 1 | 10/12 (83%) |
| V-PAD-08 | 2 | 2 | 2 | 2 | 1 | 2 | 11/12 (92%) |
| V-AAA-01 | 2 | 2 | 2 | 2 | 2 | 2 | 12/12 (100%) |
| V-AAA-02 | 2 | 2 | 2 | 2 | 1 | 2 | 11/12 (92%) |
| V-AAA-03 | 2 | 1 | 2 | 2 | 1 | 2 | 10/12 (83%) |
| V-AAA-04 | 2 | 2 | 2 | 2 | 2 | 2 | 12/12 (100%) |
| V-AAA-05 | 2 | 2 | 2 | 2 | 2 | 2 | 12/12 (100%) |
| V-AAA-06 | 1 | 1 | 2 | 2 | 1 | 1 | 8/12 (67%) |
| V-AAA-07 | 2 | 2 | 2 | 2 | 2 | 2 | 12/12 (100%) |
| V-AAA-08 | 2 | 2 | 2 | 2 | 1 | 2 | 11/12 (92%) |
| V-CVI-01 | 2 | 1 | 2 | 2 | 2 | 2 | 11/12 (92%) |
| V-CVI-02 | 2 | 2 | 2 | 2 | 2 | 1 | 11/12 (92%) |
| V-CVI-03 | 2 | 2 | 2 | 2 | 2 | 2 | 12/12 (100%) |
| V-CVI-04 | 2 | 2 | 2 | 2 | 2 | 2 | 12/12 (100%) |
| V-CVI-05 | 2 | 2 | 2 | 2 | 2 | 2 | 12/12 (100%) |
| V-CVI-06 | 2 | 2 | 2 | 2 | 1 | 2 | 11/12 (92%) |
| V-CVI-07 | 2 | 2 | 2 | 2 | 2 | 2 | 12/12 (100%) |
| V-CVI-08 | 2 | 2 | 2 | 2 | 1 | 2 | 11/12 (92%) |
| V-ISR-01 | 2 | 2 | 2 | 2 | 2 | 2 | 12/12 (100%) |
| V-ISR-02 | 2 | 2 | 2 | 2 | 2 | 1 | 11/12 (92%) |
| V-ISR-03 | 2 | 2 | 2 | 2 | 2 | 2 | 12/12 (100%) |
| V-ISR-04 | 2 | 2 | 1 | 2 | 1 | 2 | 10/12 (83%) |
| V-ISR-05 | 2 | 2 | 2 | 2 | 1 | 2 | 11/12 (92%) |
| V-ISR-06 | 1 | 2 | 1 | 2 | 1 | 2 | 9/12 (75%) |
| V-ISR-07 | 2 | 2 | 2 | 2 | 2 | 2 | 12/12 (100%) |
| V-ISR-08 | 2 | 2 | 1 | 2 | 1 | 2 | 10/12 (83%) |
| V-NEURO-01 | 2 | 2 | 2 | 2 | 1 | 2 | 11/12 (92%) |
| V-NEURO-02 | 2 | 2 | 2 | 2 | 2 | 2 | 12/12 (100%) |
| V-NEURO-03 | 2 | 2 | 2 | 2 | 2 | 2 | 12/12 (100%) |
| V-NEURO-04 | 2 | 2 | 2 | 2 | 2 | 2 | 12/12 (100%) |
| **V-NEURO-05 ★** | 2 | 2 | 2 | 2 | 2 | 2 | 12/12 (100%) |
| V-NEURO-06 | 1 | 2 | 2 | 2 | 2 | 1 | 10/12 (83%) |
| V-NEURO-07 | 2 | 2 | 2 | 2 | 2 | 2 | 12/12 (100%) |
| V-NEURO-08 | 2 | 2 | 2 | 2 | 2 | 2 | 12/12 (100%) |
| **PAD Subtotal (Rater 2): 86/96 (90%) \| C1:14 C2:16 C3:16 C4:16 C5:9 C6:15** | | | | | | | |
| **AAA Subtotal (Rater 2): 88/96 (92%) \| C1:15 C2:14 C3:16 C4:16 C5:12 C6:15** | | | | | | | |
| **CVI Subtotal (Rater 2): 92/96 (96%) \| C1:16 C2:15 C3:16 C4:16 C5:14 C6:15** | | | | | | | |
| **ISR Subtotal (Rater 2): 87/96 (91%) \| C1:15 C2:16 C3:13 C4:16 C5:12 C6:15** | | | | | | | |
| **NEURO Subtotal (Rater 2): 93/96 (97%) \| C1:15 C2:16 C3:16 C4:16 C5:15 C6:15** | | | | | | | |
| **GRAND TOTAL (Rater 2): 446/480 (93%)** | | | | | | | |

*★ V-NEURO-05: pre-designated sentinel vignette. M2 was the only model receiving C6=2 from both raters on this vignette.*

*Supplementary File S3B | M2 Claude Sonnet 4.6 Scoring Report | When AI Grades AI | Dinc R, Ardic N | 2026*
