# Supplementary material for: Systematic Scoring Differences in Large Language Model–Based Clinical Benchmarking Reveal Evaluator-Dependent Divergence: Controlled Evaluation Study
Source: JMIR AI. 2026 Oct 26;5:e103962. doi: 10.2196/103962 (PMC13705216; doi:10.2196/103962)
Supplement: Supplementary file 7 [file ai-v5-e103962-s006.docx]

**SUPPLEMENTARY FILE S3C**

**Scoring Report — M3: Gemini 1.5 Pro**

When AI Grades AI: Systematic Scoring Differences in LLM-Based Clinical Benchmarking Reveal Evaluator-Dependent Divergence

*Dinc R, Ardic N | INVAMED / Med-International UK | May 2026*

| **Model evaluated** | **M3 — Gemini 1.5 Pro (Google DeepMind; accessed via gemini.google.com, May 2026)** |
| --- | --- |
| **Rater 1** | Claude Sonnet 4.6 (also evaluated as M2) \| Score: 431/480 (90%) |
| **Rater 2** | **GPT-4o (also evaluated as M1) \| Score: 273/480 (57%)** |
| **Rubric** | C1–C6, 0–2 per criterion; maximum 12 per vignette; maximum 480 total |
| **Inter-rater delta (R2−R1)** | **-158 points** |

# 1. Criterion-Level Scores

| **Criterion** | **Rater 1 (Claude) Score** | **Rater 1 %** | **Rater 2 (GPT-4o) Score** | **Rater 2 %** |
| --- | --- | --- | --- | --- |
| C1 — Guideline concordance | **78/80** | 98% | **42/80** | 53% |
| C2 — Anatomical appropriateness | **79/80** | 99% | **41/80** | 51% |
| C3 — Contraindication recognition | **78/80** | 98% | **45/80** | 56% |
| C4 — Reasoning quality | **78/80** | 98% | **64/80** | 80% |
| C5 — Hallucination/error rate | **79/80** | 99% | **44/80** | 55% |
| **C6 — PARM Reflection** | **39/80** | 49% | **37/80** | 46% |
| **TOTAL** | **431/480** | 90% | **273/480** | 57% |

*Amber shading ≥75%; green ≥90%. † C6 (PARM Reflection) highlighted: the criterion showing the most notable evaluator-dependent variation across raters.*

# 2. Domain-Level Scores

| **Domain** | **R1 Score** | **R1 %** | **R2 Score** | **R2 %** | **R1+R2 Total** | **%** | **Dominant pattern (R2)** |
| --- | --- | --- | --- | --- | --- | --- | --- |
| **PAD** | *—* | *—* | 78/96 | 81% | *—* | *—* | *Mostly clinically relevant; several device-selection overstatements* |
| **AAA** | *—* | *—* | 59/96 | 61% | *—* | *—* | *Several vignette-ID/context mismatches* |
| **CVI** | *—* | *—* | 28/96 | 29% | *—* | *—* | *Major domain contamination with PAD/AAA/neuro cases* |
| **ISR** | *—* | *—* | 38/96 | 40% | *—* | *—* | *Multiple non-ISR answers under ISR IDs* |
| **NEURO** | *—* | *—* | 70/96 | 73% | *—* | *—* | *Strong acute stroke/SAH/carotid answers; one major mismatch* |
| **TOTAL** | **431/480** | 90% | **273/480** | 57% | — | — |  |

*R1 domain-level breakdown not retained at individual criterion level for Rater 1. R2 = Rater 2 (GPT-4o) verified domain scores from original scoring records.*

# 3. Vignette Score Matrix — Rater 2 (GPT-4o)

*Per-vignette scores assigned by Rater 2 (GPT-4o) across all 40 vignettes and 6 criteria. These are the externally verified Rater 2 scores from the original scoring records.*

| **Vignette** | **C1** | **C2** | **C3** | **C4** | **C5** | **C6** | **Total / Key gap** |
| --- | --- | --- | --- | --- | --- | --- | --- |
| V-PAD-01 | 1 | 1 | 2 | 2 | 2 | 1 | 9/12 (75%) |
| V-PAD-02 | 2 | 2 | 2 | 2 | 2 | 2 | 12/12 (100%) |
| V-PAD-03 | 2 | 2 | 1 | 2 | 2 | 1 | 10/12 (83%) |
| V-PAD-04 | 2 | 2 | 2 | 2 | 1 | 1 | 10/12 (83%) |
| V-PAD-05 | 2 | 2 | 2 | 2 | 1 | 2 | 11/12 (92%) |
| V-PAD-06 | 1 | 1 | 2 | 2 | 1 | 2 | 9/12 (75%) |
| V-PAD-07 | 1 | 1 | 2 | 2 | 2 | 1 | 9/12 (75%) |
| V-PAD-08 | 1 | 1 | 1 | 2 | 2 | 1 | 8/12 (67%) |
| V-AAA-01 | 2 | 2 | 2 | 2 | 1 | 1 | 10/12 (83%) |
| V-AAA-02 | 1 | 1 | 2 | 2 | 2 | 2 | 10/12 (83%) |
| V-AAA-03 | 2 | 2 | 2 | 2 | 2 | 2 | 12/12 (100%) |
| V-AAA-04 | **0** | **0** | 1 | 1 | **0** | **0** | **2/12 (17%)** |
| V-AAA-05 | **0** | **0** | **0** | 1 | **0** | **0** | **1/12 (8%)** |
| V-AAA-06 | **0** | **0** | 1 | 1 | **0** | **0** | **2/12 (17%)** |
| V-AAA-07 | 1 | 1 | 2 | 2 | 2 | 2 | 10/12 (83%) |
| V-AAA-08 | 2 | 2 | 2 | 2 | 2 | 2 | 12/12 (100%) |
| V-CVI-01 | 2 | 2 | 2 | 2 | 2 | 1 | 11/12 (92%) |
| V-CVI-02 | **0** | **0** | **0** | 1 | **0** | **0** | **1/12 (8%)** |
| V-CVI-03 | **0** | **0** | **0** | 1 | **0** | **0** | **1/12 (8%)** |
| V-CVI-04 | 2 | 2 | 2 | 2 | 2 | 1 | 11/12 (92%) |
| V-CVI-05 | **0** | **0** | **0** | 1 | **0** | **0** | **1/12 (8%)** |
| V-CVI-06 | **0** | **0** | **0** | 1 | **0** | **0** | **1/12 (8%)** |
| V-CVI-07 | **0** | **0** | **0** | 1 | **0** | **0** | **1/12 (8%)** |
| V-CVI-08 | **0** | **0** | **0** | 1 | **0** | **0** | **1/12 (8%)** |
| V-ISR-01 | 2 | 2 | 2 | 2 | 2 | 2 | 12/12 (100%) |
| V-ISR-02 | **0** | **0** | **0** | 1 | **0** | **0** | **1/12 (8%)** |
| V-ISR-03 | **0** | **0** | **0** | 1 | **0** | **0** | **1/12 (8%)** |
| V-ISR-04 | 1 | 1 | 1 | 2 | 2 | 1 | 8/12 (67%) |
| V-ISR-05 | **0** | **0** | **0** | 1 | **0** | **0** | **1/12 (8%)** |
| V-ISR-06 | **0** | **0** | 1 | 1 | **0** | **0** | **2/12 (17%)** |
| V-ISR-07 | 2 | 2 | 2 | 2 | 2 | 2 | 12/12 (100%) |
| V-ISR-08 | **0** | **0** | **0** | 1 | **0** | **0** | **1/12 (8%)** |
| V-NEURO-01 | 2 | 2 | 1 | 2 | 1 | 1 | 9/12 (75%) |
| V-NEURO-02 | 2 | 2 | 2 | 2 | 2 | 2 | 12/12 (100%) |
| V-NEURO-03 | 2 | 2 | 2 | 2 | 2 | 2 | 12/12 (100%) |
| V-NEURO-04 | 2 | 2 | 2 | 2 | 2 | 2 | 12/12 (100%) |
| **V-NEURO-05 ★** | 1 | **0** | **0** | 1 | 1 | 1 | **4/12 (33%)** |
| V-NEURO-06 | 2 | 2 | 1 | 2 | 2 | 1 | 10/12 (83%) |
| V-NEURO-07 | 2 | 2 | 2 | 2 | 2 | 1 | 11/12 (92%) |
| V-NEURO-08 | **0** | **0** | **0** | 1 | **0** | **0** | **1/12 (8%)** |
| **PAD Subtotal (Rater 2): 78/96 (81%) \| C1:12 C2:12 C3:14 C4:16 C5:13 C6:11** | | | | | | | |
| **AAA Subtotal (Rater 2): 59/96 (61%) \| C1:8 C2:8 C3:12 C4:13 C5:9 C6:9** | | | | | | | |
| **CVI Subtotal (Rater 2): 28/96 (29%) \| C1:4 C2:4 C3:4 C4:10 C5:4 C6:2** | | | | | | | |
| **ISR Subtotal (Rater 2): 38/96 (40%) \| C1:5 C2:5 C3:6 C4:11 C5:6 C6:5** | | | | | | | |
| **NEURO Subtotal (Rater 2): 70/96 (73%) \| C1:13 C2:12 C3:10 C4:14 C5:12 C6:10** | | | | | | | |
| **GRAND TOTAL (Rater 2): 273/480 (57%)** | | | | | | | |

*★ V-NEURO-05: pre-designated sentinel vignette. C6=2 was achieved only by M2 on V-NEURO-05 under both raters. Rater 2 = GPT-4o (context-fidelity scoring approach).*

*Supplementary File S3C | M3 Gemini 1.5 Pro Scoring Report | When AI Grades AI | Dinc R, Ardic N | 2026*
